# A green strategy for the synthesis of sulfone derivatives of *p*-methylaminophenol: Kinetic evaluation and antibacterial susceptibility

**DOI:** 10.1038/s41598-017-04581-0

**Published:** 2017-06-30

**Authors:** Davood Nematollahi, Sadegh Khazalpour, Mina Ranjbar, Shima Momeni

**Affiliations:** Faculty of Chemistry, Bu-Ali Sina University, Hamedan, 65178-38683 Iran

## Abstract

This is one of the few examples in which the diverse products have been synthesized just by changing the applied potential. The synthesis of sulfonyl derivatives of *p*-methylaminophenol were carried out by reaction of the electrogenerated *p*-methylquinoneimine with sulfinic acids. Various types of mono (MSP), bis (BSP) and tris (TSP) sulfonyl *p*-methyl aminophenols were obtained by changing the electrode potential, in one pot under green conditions. The mono sulfonyl-*p*-(methylamino)phenol derivatives (MSP) were assessed for their ***in vitro*** antibacterial activity against the gram positive (*Staphylococcus aureus*) and gram negative (*Escherichia coli*) strains. It was found that the tested compounds were more active against *Staphylococcus aureus* than *Escherichia coli*. We also found that the antimicrobial activity of MSP derivatives to vary in the order MSP_4_ (R = CH_3_) > MSP_1_ (R = *p*-tolyl) ≈ MSP_2_ (R = phenyl) > MSP_3_ (R = *p*-ClC_6_H_4_). Moreover, the observed homogeneous rate constants (*k*_obs_) of the reaction of *p*-methyl quinoneimine with sulfinic acids were estimated in various pH values, based on the *EC* and *ECEC* mechanisms, by comparing the simulated cyclic voltammograms with the experimental ones.

## Introduction

The control of selectivity is one of the important challenges in organic syntheses^1–6^. To overcome to this problem, a number of organic and metal catalyst systems has been examined^1–6^. However, they have the disadvantages of safety problems and heavy metal pollution. Organic electrochemical synthesis provides a powerful strategy for the synthesis of organic compounds in both laboratory and industry scale^7–13^. In this method, the electrons are considered as clean reagents, so that this method can be considered as a green technique. Another important feature of this method, which is used in this work, is its selectivity towards synthesis of products. This unique feature arises from the fact that, the different active intermediates can be provided just by changing the electrode potential^7–13^.

*p*-Aminophenol is commercially significant as a versatile intermediate in the manufacture of chemical dye, drugs such as acetaminophen, photographic developer and anticorrosion agents^14–17^. In particular, it is known that some aminophenol derivatives have antiviral activity against flu A and simple herpes^18^. It is also known that, the aminophenols containing a sulphone group in addition to activity against flu A and simple herpes have excellent activity against the HIV infection^19^. On the other hand, it is found that, diphenylsulfone compounds exhibited antibacterial activity^20^. For example, 4,4-diaminodiphenylsulfone (dapsone) is a bacteriostatic drug that inhibits dihydrofolic acid synthesis by competition with *para*-aminobenzoic acid^21^. The oxidation of sulfides or sulfoxides using peracids or hydrogen peroxide, addition reactions to alkenes and alkynes, Friedel–Crafts-type sulfonylation of arenes in the presence of a Lewis or Brønsted acid catalyst, and alkylation of sulfinate salts, are four traditional methods for the synthesis of sulfones^22^. The excess oxidizing agent, high temperatures, harsh reaction conditions, low regioselectivity, the need for stoichiometric amounts of the catalyst, the generation of hazardous waste and formation of a mixture of isomers are the main disadvantages of these methods^23–27^.

We assume that the compounds containing both aminophenol and diphenylsulfone moieties, may show promising biological activities. So, the synthesis of these type of compounds by a simple method without the above discussed disadvantages using a green and controllable strategy is the main object of this paper.

## Results and Discussion

**Figure 1:**
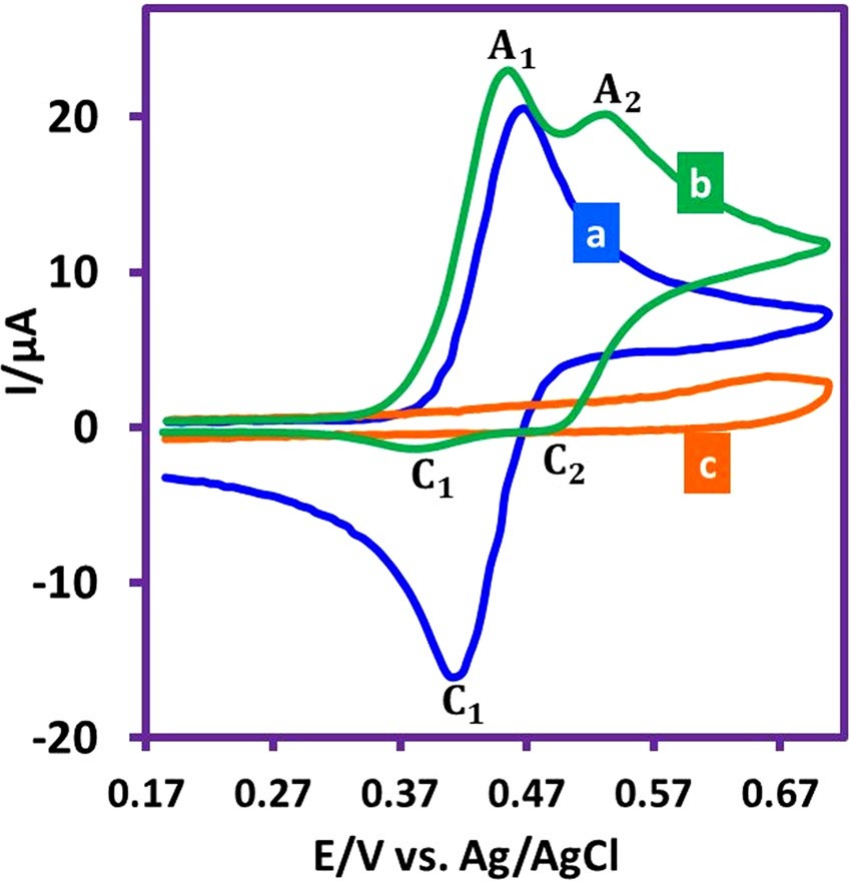
Cyclic voltammograms of: (a) **MAP** (1.0 mM), (b) **MAP** (1.0 mM) + **BS** (1.0 mM) and (c) **BS** (1.0 mM) in aqueous phosphate buffer (*c* = 0.2 M, pH = 2.0). Working electrode: glassy carbon electrode. Scan rate 100 mV s^−1^. Temperature = 25 ± 1 °C.

Figure 1a shows the cyclic voltammogram (CV) of *p*-methylaminophenol (**MAP**) in aqueous phosphate buffer (*c* = 0.2 M, pH = 2.0). It includes a well-defined anodic (A_1_)/cathodic (C_1_) peak couple at *E*_1/2_ = 0.44 V versus Ag/AgCl. These peaks (A_1_ and C_1_) are attributed to two-electron oxidation of **MAP** to *p*-methylquinoneimine (**MQI**) and vice versa, respectively. Under these conditions, *I*_pC1_/*I*_pA1_ is near to one, which can be regarded as a criterion for the consistency of electrogenerated **MQI** under the experimental conditions.

Figure 1b shows the CV of **MAP** in the presence of benzensulfinic acid (**BS**) under the same conditions as Fig. 1a. Comparison of CVs 1a and 1b shows three important differences: (i) the *I*_pC1_/*I*_pA1_ is near to one in Fig. 1a, but is about 0.06 in Fig. 1b, (ii) contrary to Fig. 1b shows two couples of peaks (A_1_/C_1_ and A_2_/C_2_), (iii) *E*_pA1_ in Fig. 1b is less positive than that of Fig. 1a. In addition, our data show that two factors: (i) potential scan rate and (ii) **BS** concentration are effective in the peak current ratio (*I*_pC1_/*I*_pA1_) of **MAP** in the presence of **BS**, so that it decreases with increasing **BS** concentration and decreasing the potential scan rate.

All of these results are in agreement with the participation of electrogenerated **MQI** in the Michael addition reaction with **BS** ^28–34^. Fig. 1c is the cyclic voltammogram of **BS** which indicates that in the studied potential range, **BS** is not electroactive.

**Figure 2:**
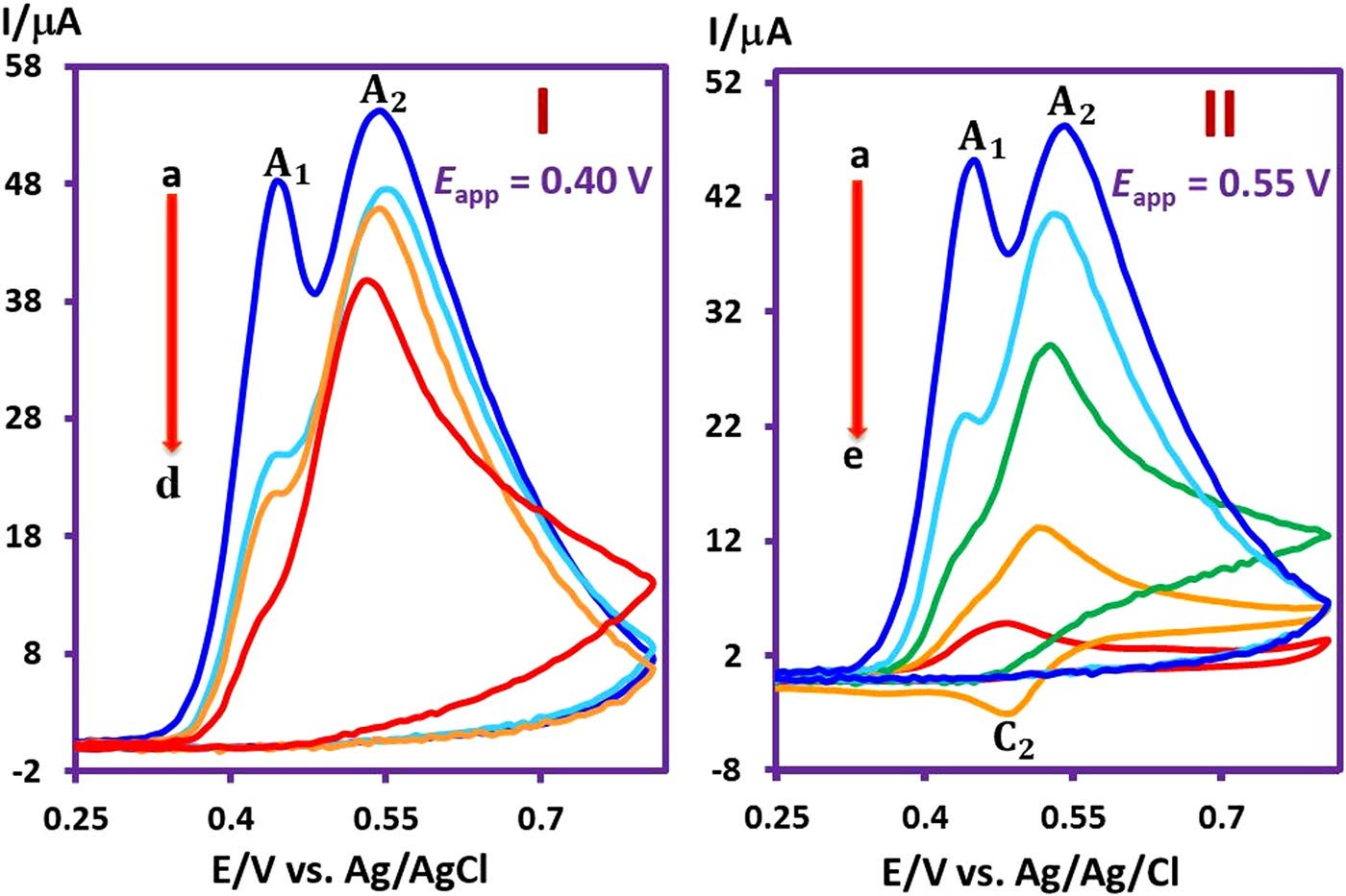
Cyclic voltammograms of **MAP** (0.25 mmol) in the presence of **BS** (0.5 mmol) during CPC at 0.40 (part I) and 0.55 (part II) V *vs*. Ag/AgCl. Part I: the consumed charge is (a) 0, (b) 16, (c) 32 and (d) 47 C. Part II: the consumed charge is (a) 0, (b) 40, (c) 70, (d) 90 and (e) 110 C. Other conditions are as same as Fig. 1.

Controlled-potential coulometry at *E*_app_ < *E*_pA1_ (*E*_app_ = 0.40 V vs. Ag/AgCl) was used to determine the number of the electrons transferred (*n*) for the anodic oxidation of **MAP** in the presence of **BS** (Fig. 2I). The cyclic voltammetry during the progress of CPC indicates two significant changes: (i) decreasing *I*_pA1_ and (ii) increasing *I*_pA2_. The *I*_pA1_ reaches zero when the charge consumption was about 2e^−^ (*n* = 1.95) per molecule of **MAP**.

**Figure 3:**
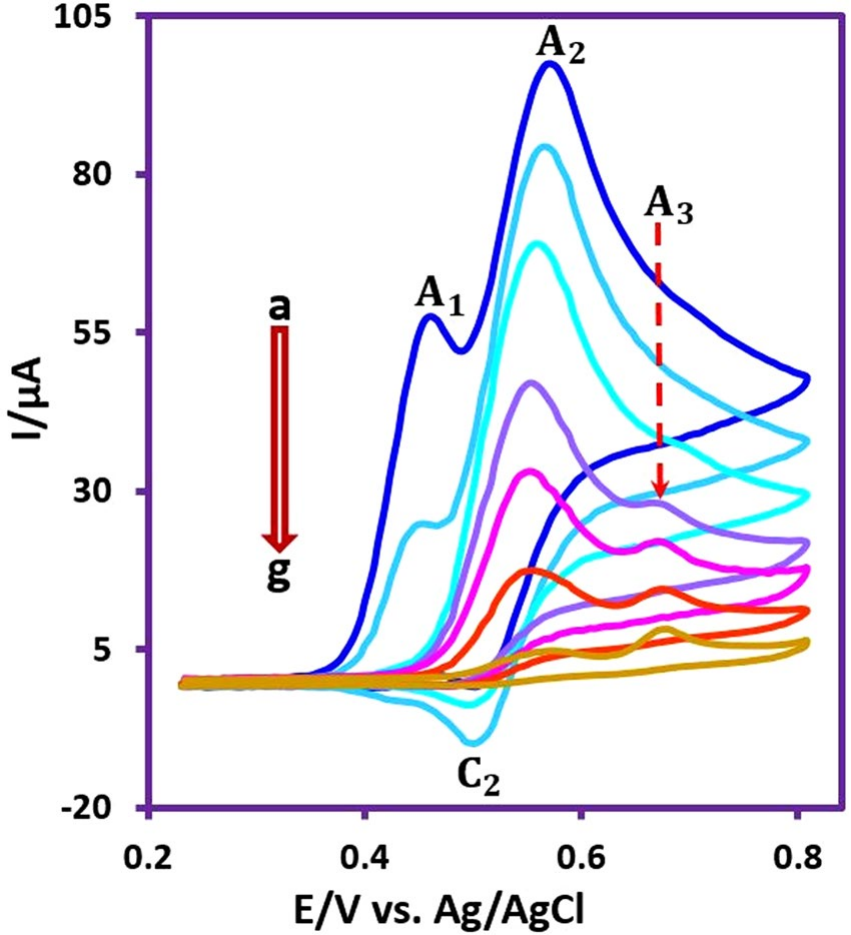
Cyclic voltammograms of **MAP** (0.25 mmol) in the presence of **MS** (0.75 mmol) during CPC at 0.55 V vs. Ag/AgCl, after consumption of (a) 0, (b) 35, (c) 75, (d) 105, (e) 120, (f) 135 and (g) 151 C. Other conditions are as same as Fig. 1.

The CPC was also performed at *E*_app_ > *E*_pA2_ (*E*_app_ = 0.55 V vs. Ag/AgCl) (Fig. 2II). In comparison with CPC at *E*_app_ = 0.40, it shows two significant differences: first, the number of electrons is increased from two to four electrons (*n* = 4.5). Second, both anodic peaks have been removed. Another CPC was performed in the same solvent/electrolyte system containing **MAP** (0.25 mmol) in the presence of methanesulfinic acid (**MS**) (0.75 mmol) at *E*_app_ = 0.55 V vs. Ag/AgCl) (Fig. 3). Contrary to Fig. 2, during the progress of CPC, a new anodic peak (A_3_) appeared at more positive potentials than the main wave. Under these conditions, all anodic and cathodic peaks disappear at the end of CPC after consumption of 151 C (*n = *6.3) of electricity. The following evidences along with the spectroscopic data of the final products were used to propose the following mechanism for the electrochemical oxidation of **MAP** at different applied potentials in the presence of sulfone nucleophiles.

**Figure 4:**
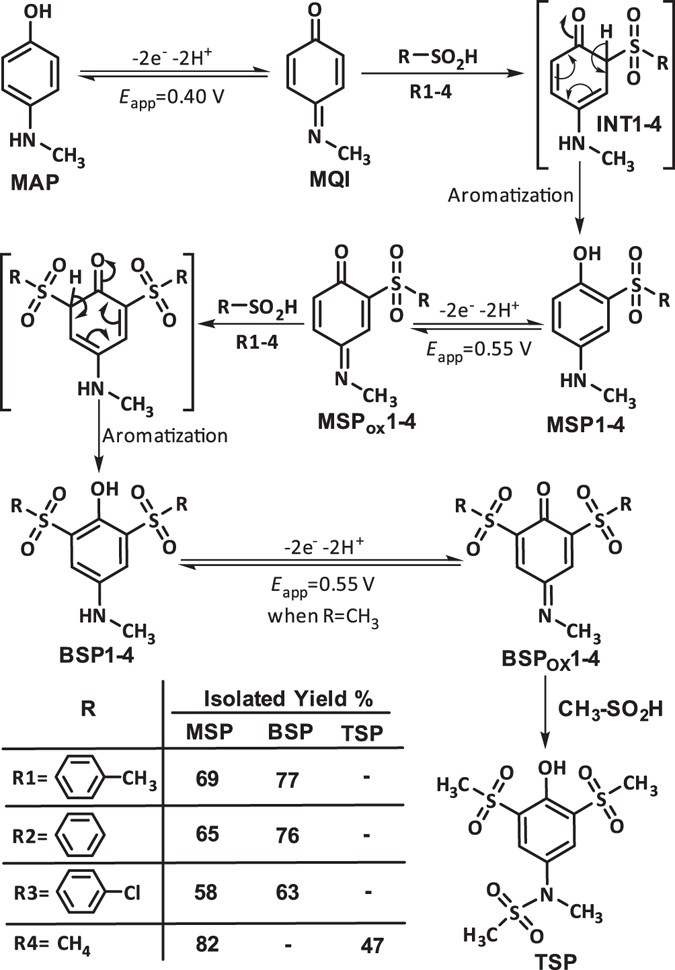
Electrochemical oxidation pathway of **MPA** in the presence of sulfone compounds.

**Figure 5:**
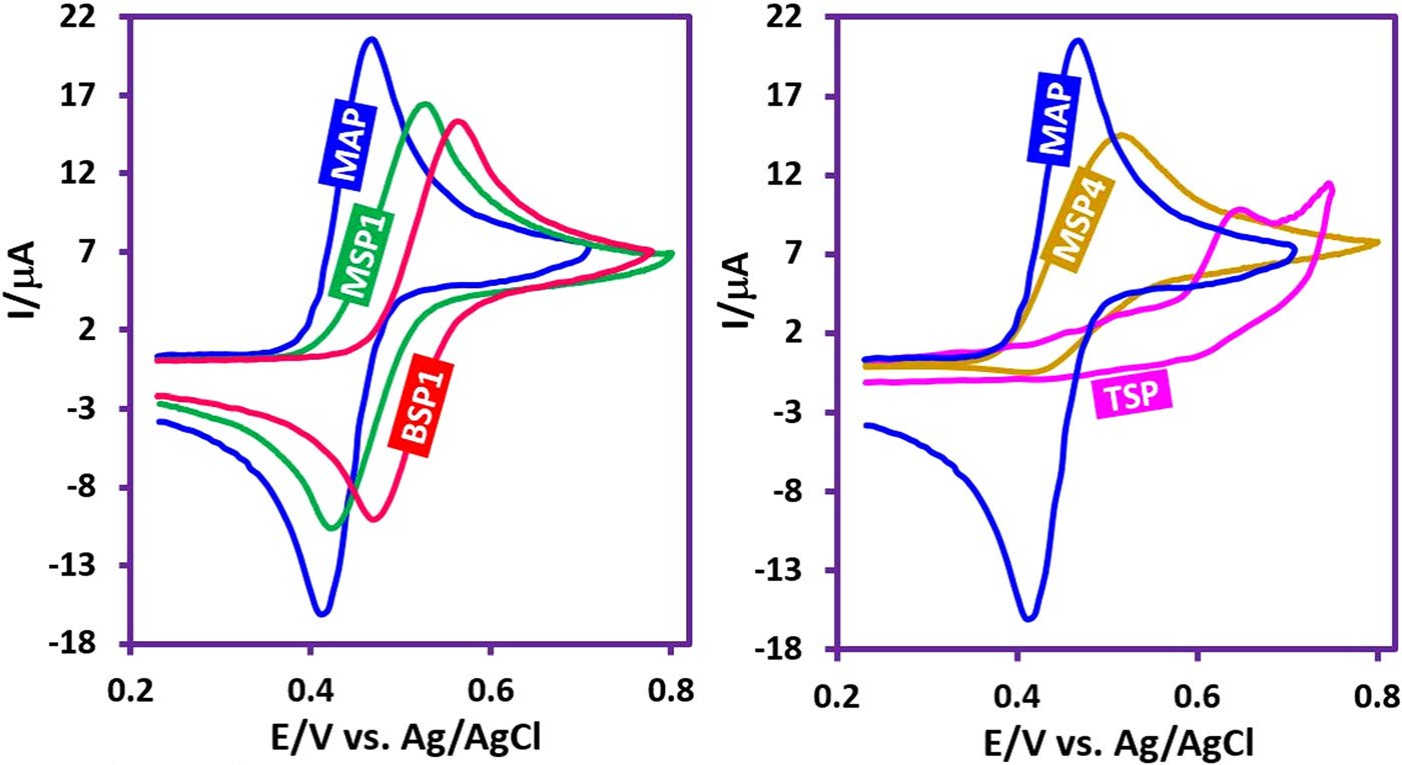
Cyclic voltammograms of: **MAP** (1.0 mM), **MSP1** (0.7 mM), **BSP1** (0.5 mM), **MSP4** (0.5 mM) and **TSP** (0.25 mM). Solvent for **MAP**: aqueous phosphate buffer, *c* = 0.2 M, pH = 2.0. Solvent for **MSP1**, **BSP1**, **MSP4** and **TSP**: aqueous phosphate buffer (*c* = 0.2 M, pH = 2.0)/ethanol (80/20, v/v). Working electrode: glassy carbon electrode. Scan rate 100 mV s^−1^. Temperature = 25 ± 1 °C.

As indicated in Fig. 4, when the applied potential is 0.40 V vs. Ag/AgCl, the nucleophilic attack of the sulfone compounds on the electrogenerated **MQI** would result in **INT1-4** which undergoes aromatization to afford the mono sulfonyl-*p*-(methylamino)phenol derivatives (**MSP1–4**) as the final products. **MSP1–4** is structurally a *p*-methylaminophenol derivative, however, because of the presence of an electron-withdrawing sulfonyl group in its structure, its oxidation is more difficult than the oxidation of **MAP** (Fig. 5) and thus, its oxidation at the anode is avoided.

With increasing the applied potential to 0.55 V vs. Ag/AgCl, the oxidation of **MSP1–4** becomes possible and **MSP**_**ox**_ **1-4** is formed. The addition of sulfone nucleophile to **MSP**_**ox**_ **1-4** followed by aromatization, converts **MSP**_**ox**_ **1-4** into bis sulfonyl-*p*-(methylamino)phenol derivatives (**BSP1-4**). Since the oxidation of **BSP1-4** is more difficult than the oxidation of **MSP1–4** and **MAP** (Fig. 5), its oxidation was stopped due to the presence of the two electron-withdrawing sulfonyl groups as well as the insolubility of **BSP1-4** in the electrolysis medium. A remarkable finding in this study is related to the role of methanesulfinic acid (**MS**) as a nucleophile. Despite several attempts to synthesize **BSP4**, no bis sulfonyl derivative has been isolated and unexpectedly a tris sulfonyl compound, **TSP**, which is a new sulphonamide molecule was obtained via the oxidation of **BSP4** and attack of **MS** to electrogenerated **BSP**_**ox**_ **4**. The oxidation of **BSP4** at 0.55 V vs. Ag/AgCl, can be related to the lower electron-withdrawing ability of the methyl-sulfony group compared with aryl-sulfony groups that causes the oxidation potentials of bis and tris sulfonyl compounds do not have any significant differences. In addition, higher nucleophilicity and lower steric effect of **MS** compared with aryl-sulfone nucleophiles and more solubility of the products containing **MS**, can also be effective in the observed behavior. According to Fig. 4, the anodic peaks A_1_, A_2_ and A_3_ pertain to the oxidation of **MAP**, **MSP** and **BSP** to the **MQI**, **MSP**_**ox**_ and **BSP**_**ox**_, respectively.

**MQI** is a bis-Michael acceptor (*ortho* and *meta* of the phenolic group) and can be attacked by the sulfone nucleophiles from two sites to yield two isomers (for example, 4-(methylamino)-*m*-(methylsulfonyl)phenol and 4-(methylamino)-*o*-(methylsulfonyl) phenoltypes). However, the comparison of simulated ^1^H NMR^35^ for the possible compounds and experimental ^1^H NMR of the final product (See SI), confirms synthesis of *ortho* derivative. The protonation of the amino group in **MAP** at pH = 2.0, makes the *ortho* position in **MQI** more reactive than *meta* position for the addition reaction.

**Figure 6:**
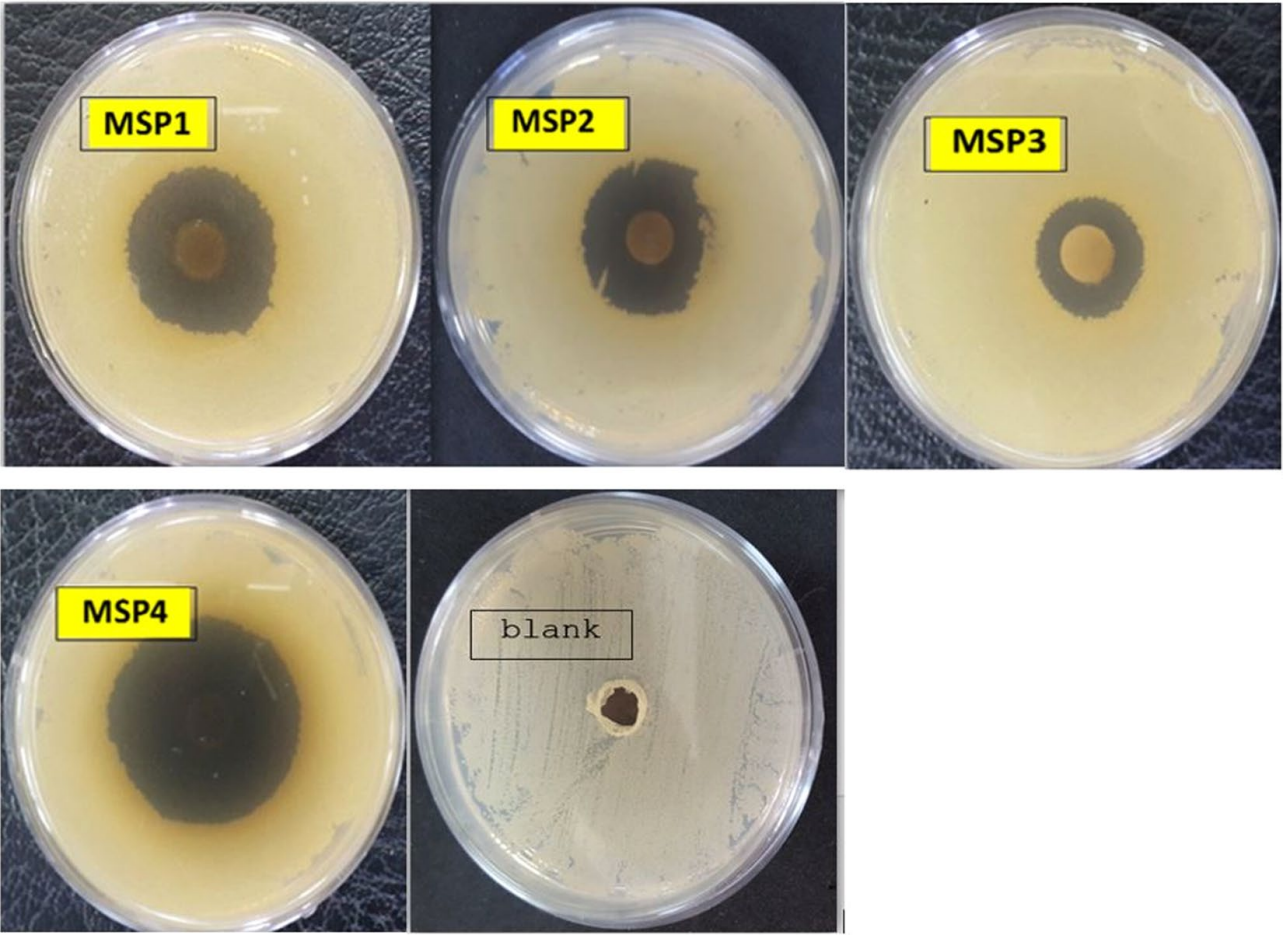
Inhibition zone diameters (mm) obtained of *Staphylococcus aureus* in disc diffusion test for **MSP1–4** (5 mg) and solvent.

The compounds **MSP1–4** were tested for the antibacterial activity against the Gram positive (*Staphylococcus aureus*) (Fig. 6) and Gram negative (*Escherichia coli*) strains. The results indicated that *Staphylococcus aureus* was more sensitive to **MSP1–4** than *Escherichia coli* (See SI). The existence of an exterior membrane along with a collection of resisting pumps against drugs in the negative gram bacteria, makes a very effective barrier from this group of bacteria against antibacterial growth and operation. We also found that the antimicrobial activity of **MSP** derivatives to vary in the order **MSP**_**4**_ (R=CH_3_) > **MSP**_**1**_ (R=*p*-tolyl) > **MSP**_**2**_ (R=phenyl) > **MSP**_**3**_ (R=*p*-ClC_6_H_4_).

**Figure 7:**
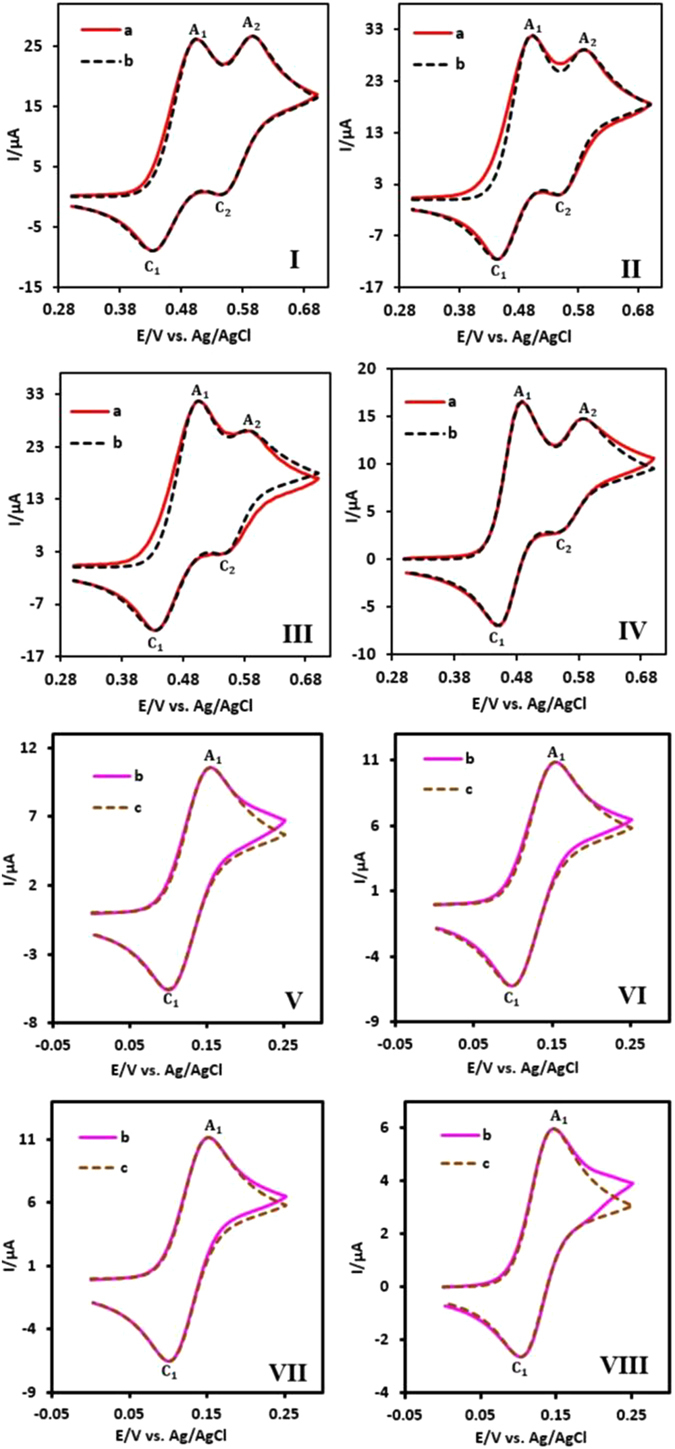
Experimental (a) and simulated (b) cyclic voltammograms of **MAP** (1 mM) in the presence of (**I** and **V**) *p*-toluenesulfinic acid (0.5 mM), (**II** and **VI**) benzensulfinic acid (0.5 mM), (**III** and **VII**) *p*-chlorosulfinic acid (0.5 mM) and (**IV** and **VIII**) methanesulfinic acid (0.5 mM) at glassy carbon electrode. In **I**–**IV**; solvent, aqueous HClO_4_ (0.1 M) and scan rate: 80 mV/s. In **V**–**VIII**, solvent, aqueous phosphate buffer (pH = 6.0, *c* = 0.2 M) and scan rate: 10 mV/s. Temperature = 25 ± 1 °C.

**Figure 8:**
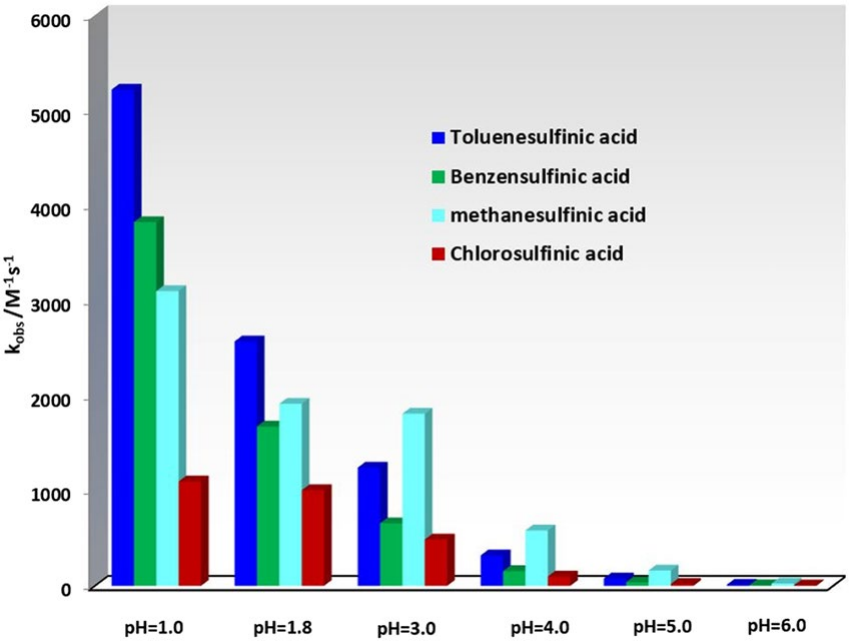
Observed homogeneous rate constants (*k*_obs_) of the reaction of **MQI** with the sulfone nucleophiles at different pH values.

The observed homogeneous rate constants (*k*_obs_) of the reaction of **MQI** with the sulfinic nucleophiles was studied based on *ECEC* (Fig. 7, parts I–IV) and *EC* (Fig. 7, parts V–VIII) mechanisms, by computer simulation of the experimental cyclic voltammograms. Our data shows that, *k*_obs_ is strongly dependent to pH so that, it increases with decreasing pH value. The protonation of the nitrogen atom in **MQI**, makes it more reactive toward the addition reaction and is responsible for increasing *k*_obs_. In addition, the results displays that, *k*_obs_ is also dependent directly to the electron donating ability of the nucleophile, so that, it varies in the order methanesulfinic acid > *p*-toluenesulfinic acid > banzenesulfinic acid > *p*-chlorosulfinic acid (Fig. 8).

## Conclusions

In summary, the electrochemical synthesis of the title compounds has two advantages over conventional methods. Firstly, in this method, a variety of products could be formed just by changing the applied potential. In this work, the synthesis of some mono and bis (or tris) sulfonyl compounds were carried out by electrochemical oxidation of **MAP** in the presence of arylsulfinic acids only with changing the applied potential from 0.40 to 0.55 V vs. Ag/AgCl. Secondly, this method proceeds in a single step, in ambient conditions, without dealing with strong acids/base, organic solvent and catalysis, under green and mild conditions with high atom economy.

## Materials and Methods

### Apparatus and Reagents

Cyclic voltammetry, controlled-potential coulometry and preparative electrolysis were performed using a Zahner pp201 potentiostat/galvanostat. Macro-scale electrolysis and controlled-potential coulometry were carried out with a three electrode system, using a Behpajooh C 2056 potentiostat equipped with a digital coulometer. The working electrode used in the voltammetry experiments was a glassy carbon disc (1.8 mm^2^ area) and platinum wire was used as counter electrode. The working electrode used in controlled-potential coulometry and synthesis was an assembly of three ordinary soft carbon plates (20 mm length, 10 mm width and 40 mm height), and large stainless steel cylinder (25 cm^2^ area) constituted the counter electrode. The working electrode potentials were measured versus Ag/AgCl (all electrodes from AZAR electrode). The electrochemical synthesis was performed under controlled-potential condition in a simple cell equipped with a magnetic stirrer. More details are described in our previous paper^36^.

*p*-Methylaminophenol (**MAP**), *p*-toluenesulfinic acid, benzensulfinic acid, *p*-chlorosulfinic acid, methanesulfinic acid, phosphoric acid and ethanol were obtained from commercial sources. These chemicals were used without further purification. The glassy carbon electrode was polished using alumina slurry (from Iran Alumina Co.).

### Electroorganic Synthesis of MSP1–4

An aqueous phosphate buffer solution (*c* = 0.2 M, pH = 2.0) (*ca*. 100 mL), containing **MAP** (0.25 mmol) and sulfone nucleophiles (*p*-toluenesulfinic acid, benzensulfinic acid, *p*-chlorosulfinic acid, methanesulfinic acid), (0.25 mmol), was electrolyzed at 0.40 V vs. Ag/AgCl, at 25 °C. The electrolysis was terminated when the decay of the current became more than 95%. At the end of electrolysis, the cell was placed in a refrigerator overnight. The precipitated solid was collected by filtration, washed copiously with distilled water. The products (**MSP1–4**) were characterized by their physical and spectroscopic data.

### Electroorganic Synthesis of BSP1-3

The synthesis of **BSP1–3** derivatives were carried out under the same conditions as described for **MAP1-4** in a solution containing **MAP** (0.25 mmol) and sulfone nucleophiles (*p*-toluenesulfinic acid, benzensulfinic acid, *p*-chlorosulfinic acid), (0.5 mmol), at 0.55 V. At the end of electrolysis, the cell was placed in a refrigerator overnight. The precipitated solid was collected by filtration, washed copiously with distilled water.

### Electroorganic Synthesis of TSP

The synthesis of **TSP** was carried out under the same conditions as described for **MAP1-4** in a solution containing **MAP** (0.25 mmol) and methanesulfinic acid (0.75 mmol), at 0.55 V vs. Ag/AgCl, at 25 °C. At the end of electrolysis, the cell was placed in a refrigerator overnight. The precipitated solid was collected by filtration, washed copiously with distilled water.

### 4-(Methylamino)-2-tosylphenol (MSP1)

Isolated yield 69%; mp: 210–212 °C (dec.). ^1^H NMR (400 MHz, DMSO-*d*_6_): *δ* 2.38 (s, 3H, methyl), 2.66 (s, 3H, methyl), 6.68 (m, 2H, aromatic), 7.07 (d, *J* = 2.4 Hz, 1H, aromatic), 7.37 (d, *J* = 8 Hz, 2H, aromatic), 7.76 (d, *J* = 8 Hz, 2H, aromatic), 9.53 (s, 1H, OH); ^13^C NMR (100 MHz, DMSO-*d*_6_): *δ* 21.5 (C-1), 30.8 (C-10), 110.3 (C-8), 118.8 (C-5), 119.9 (C-8), 126.8 (C-7), 128.2 (C-4), 129.7 (C-3), 139.3 (C-11), 143.3 (C-6,C-9), 143.8 (C-12); FT-IR (KBr): 3487, 3416 medium, O-H), 3091 (weak C-H), 1615 (medium N-H), 1492 (medium C=C), 1316, 1289 (weak S=O), 1141, 1087 (strong, S=O), 960, 818, 710, 656, 533 cm^−1^; MS (EI, 70 eV): m/z (relative intensity): 277 (M, 38), 65 (100), 93 (90), 78 (74), 51 (29).
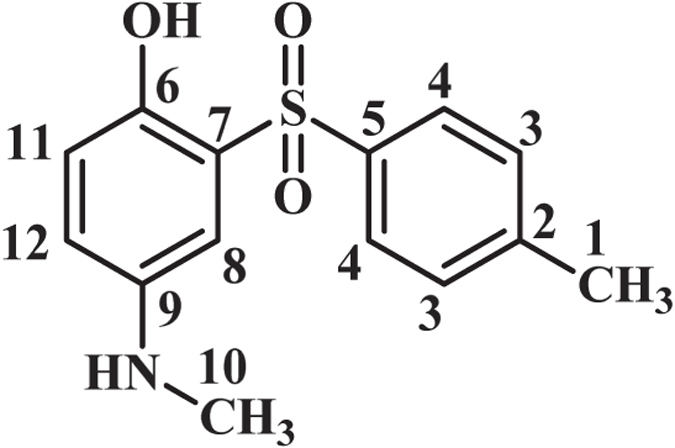


**4-(Methylamino)-2-(phenylsulfonyl)phenol (MSP2):** Isolated yield 65%; mp = 201-203 °C (dec.). ^1^H NMR (400 MHz, DMSO-*d*_6_): *δ* 2.66 (s, 3H, methyl), 6.74 (m, 2H, aromatic), 7.08 (d, *J* = 2.4 Hz, 1H, aromatic), 7.58 (t, *J* = 7.6 Hz, 2H, aromatic), 7.66 (t, *J* = 7.4 1H, aromatic), 7.89 (d, *J* = 7.6 Hz, 2H, aromatic), 5.79 (s, 1H, NH); ^13^C NMR (100 MHz, DMSO-*d*_6_): *δ* 30.8 (C-9), 110.3 (C-8), 118.9 (C-11), 120.1 (C-10), 126.5 (C-6), 128.1 (C-4), 129.2 (C-3), 133.4 (C-1), 142.1 (C-8), 143.3 (C-4), 146.5 (C-5); FT-IR (KBr): 3494 (medium, O-H), 3098 (weak C-H), 1615 (medium N-H), 1489 (medium C=C), 1315, 1289 (weak S=O), 1144, 1087 (strong, S=O), 954, 872, 733.41, 596, 510 cm^−1^; MS (EI, 70 eV): m/z (relative intensity): 263 (M, 100), 93 (54), 78 (36), 51 (29), 122 (16).
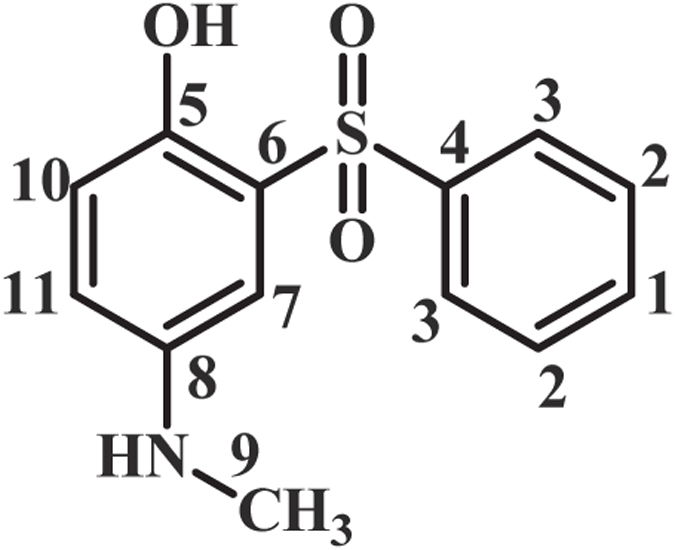


**2-((4-Chlorophenyl)sulfonyl)-4-(methylamino)phenol (MSP3):** Isolated yield 58%; mp = 139-141 °C (dec.). ^1^H NMR (400 MHz, DMSO-*d*_6_): *δ* 2.77 (s, 3H, methyl), 6.87 (m, 2H, aromatic), 7.19 (d, *J* = 2.4 Hz, 1H, aromatic), 7.76 (t, *J* = 8.8 Hz, 2H, aromatic), 8.00 (t, *J* = 8.4 1H, aromatic), 7.88 (d, *J* = 7.6 Hz, 2H, aromatic); ^13^C NMR (100 MHz, DMSO-*d*_6_): *δ* 30.4 (C-9), 109.7 (C-7), 118.4 (C-10), 120.0 (C-11), 126.5 (C-6), 128.9 (C-3), 129.7 (C-2), 137.9 (C-1), 140.4 (C-4), 142.7 (C-8), 146.2 (C-5); FT-IR (KBr): 3487 (medium, O-H), 3087 (weak C-H), 1613 (medium N-H), 1474 (medium C=C), 1321 (strong S=O), 1152, 1089 (strong, S=O), 933, 822, 754, 596, 483 cm^−1^; MS (EI, 70 eV): m/z (relative intensity): 297.3 (M,100), 93 (90), 159 (36), 41 (29), 229 (5).
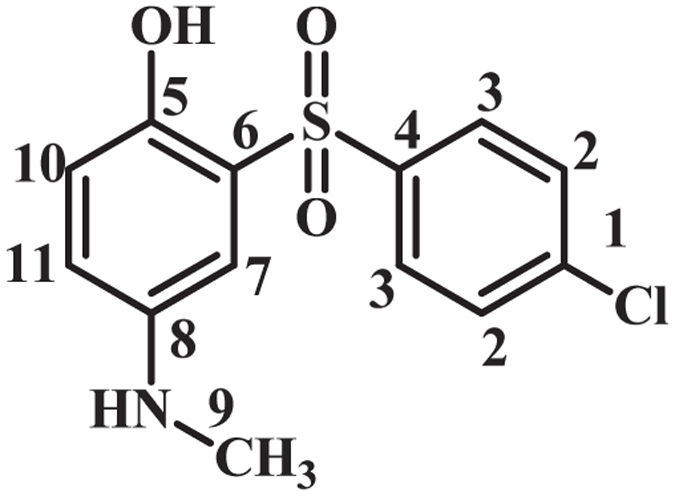


**4-(Methylamino)-2-(methylsulfonyl)phenol (MSP4):** Isolated yield 82%; mp = 187–189 °C (dec.). ^1^H NMR (400 MHz, DMSO-*d*_6_): *δ* 2.71 (s, 3H, methyl), 3.28 (s, 3H, methyl), 6.83 (m, 1H, aromatic), 6.94 (d, *J* = 8.8 Hz, 1H, aromatic), 6.97 (d, *J* = 2.8 Hz, 2H, aromatic; ^13^C NMR (100 MHz, DMSO-*d*_6_): *δ* 30.3 (C-6), 42. 3 (C-1), 109.3 (C-4), 118.2 (C-7), 118.8 (C-8), 126.5 (C-3), 142.8 (C-2), 145.8 (C-5); FT-IR (KBr): 3428, 3256 (medium, O-H), 3006 (weak C-H), 1610 (medium N-H), 1489 (medium C=C), 1302 (strong S=O), 1133, 1083 (strong, S=O), 960, 819, 749, 568, 538 cm^−1^; MS (EI, 70 eV): m/z (relative intensity): 201 (M, 43), 122 (100), 92 (52), 80 (45), 108 (23).
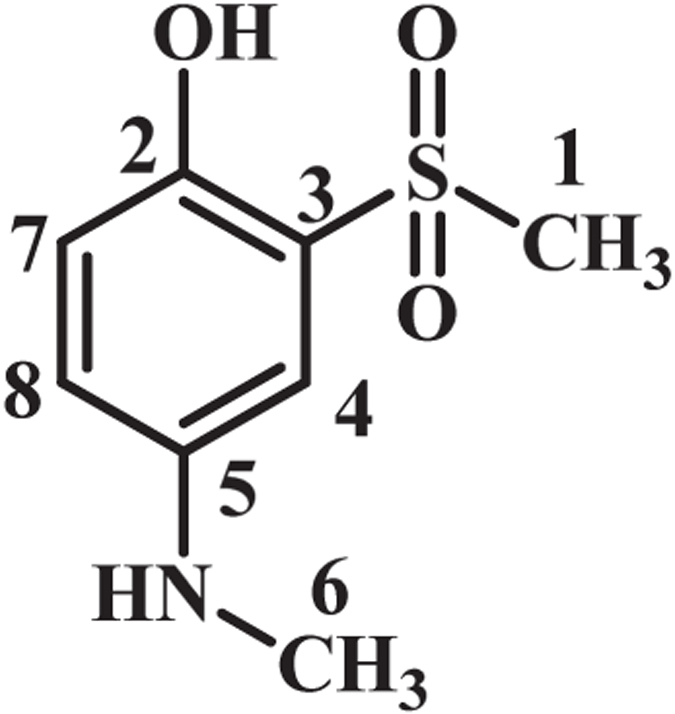


**4-(Methylamino)-2,6-ditosylphenol (BSP1):** Isolated yield: 77%; mp = 185–187 °C (dec.). ^1^H NMR (400 MHz, CDCl_3_): *δ* 2.36 (s, 6H, methyl), 2.75 (s, 3H, methyl) 7.19 (s, 2H, aromatic), 7.23 (d, *J* = 8 Hz, 4H, aromatic), 7.74 (d, *J* = 8.4 Hz, 4H, aromatic), 9.06 (s, 1H, OH); ^13^C NMR (100 MHz, CDCl_3_): *δ* 21.7 (C-1), 31.0 (C-10), 117.8 (C-8), 127.9 (C-4), 128.6 (C-7), 129.8 (C-3), 137.5 (C-5), 142.7 (C-6), 144.6 (C-9), 145.0 (C-2); FT-IR (KBr): 3550, 3470 (medium, O-H), 2924 (weak C-H), 1617 (medium C=C), 1503 (medium C=C), 1317, 1288 (weak S=O), 1138 (strong S=O), 1083, 807, 656, 571, 526 cm^−1^; MS (EI, 70 eV): m/z (relative intensity): 431 (M^.^, 100), 182 (61), 91 (38), 139 (28), 65 (25), 211(14).
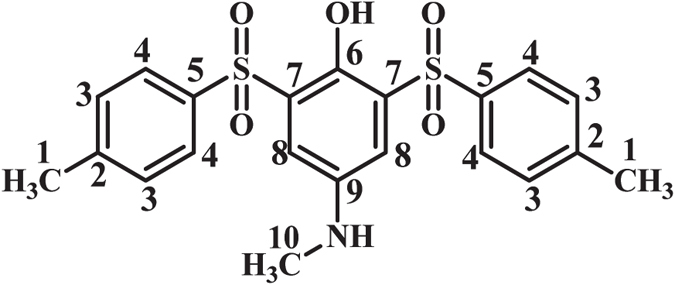


**4-(Methylamino)-2,6-bis(phenylsulfonyl)phenol (BSP2):** Isolated yield 76%; mp = 200–202 °C (dec.). ^1^H NMR (400 MHz, CDCl_3_): *δ* 3.22 (s, 3H, methyl), 7.33 (s, 2H, aromatic), 7.51 (t, *J* = 7.8 Hz, 4H, aromatic), 7.62 (t, *J* = 7.4 Hz, 2H, aromatic), 7.95 (d, *J* = 8 Hz, 4H, aromatic), 1.03 (s, 1H, OH); ^13^C NMR (100 MHz, CDCl_3_): *δ* 31.0 (C-9), 118.0 (C-7), 127.8 (C-3), 128.3 (C-4), 129.2 (C-2), 133.9 (C-1), 140.5 (C-6), 142.8 (C-5), 144.7 (C-8); FT-IR (KBr): 3427 (medium, N-H), (3344, medium, OH) 3066 (weak C-H) 1619 (weak N-H), 1501 (medium C=C), 1311, 1293 (strong S=O), 1144, 1082 (strong, S=O), 811, 726, 609, 577, 542 cm^−1^; MS (EI, 70 eV): m/z (relative intensity): 403 (M^.^, 100), 77 (87), 168 (67), 51 (50), 125 (32), 262(6).
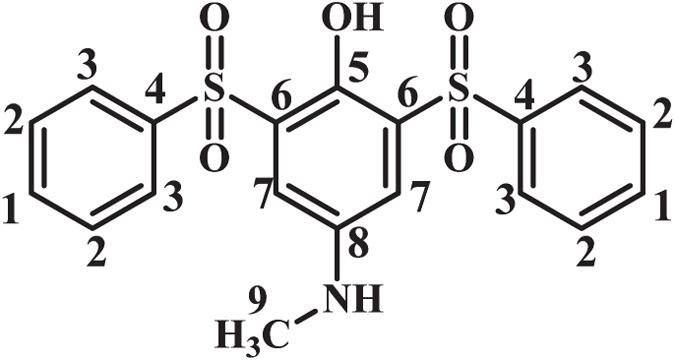


**2,6-Bis((4-chlorophenyl)sulfonyl)-4-(methylamino)phenol (BSP3):** Isolated yield 54%; mp = 166–168 °C (dec.). ^1^H NMR (400 MHz, CDCl_3_): *δ* 2.89 (s, 3H, methyl), 7.41 (s, 2H, aromatic), 7.52 (d, *J* = 8.8 Hz, 4H, aromatic), 7.90 (d, *J* = 8.4 Hz, 4H, aromatic); ^13^C NMR (100 MHz, CDCl_3_): *δ* 31.1 (C-9), 118.2 (C-7), 128.2 (C-6), 129.3 (C-3), 129.6 (C-2), 138.8 (C-1), 140.8 (C-4), 142.7 (C-5), 144.6 (C-8); FT-IR (KBr): 3410 (medium, N-H), 3307 (medium, OH), 3091 (weak C-H), 1619 (weak N-H), 1573 (medium C=C), 1318, 1299 (strong S=O), 1144, 1091 (strong, S=O), 1022, 806, 752, 618, 581 cm^−1^; MS (EI, 70 eV): m/z (relative intensity): 471 (M^.^, 91), 111 (100), 75 (75), 159 (52), 131(42), 296(8).
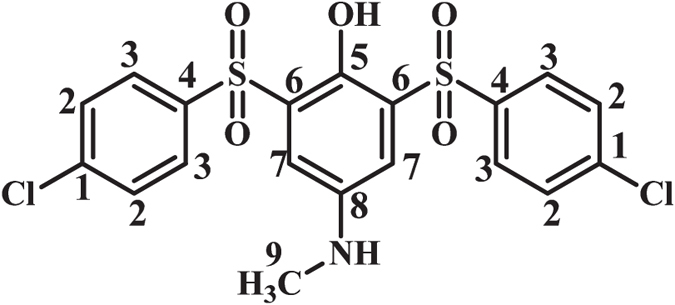


***N*** **-(4-Hydroxy-3,5-bis(methylsulfonyl)phenyl)-** ***N*** **-methylmethanesulfonamide:** Isolated yield 47%; mp = 213–215 °C (dec.). ^1^H NMR 400 MHz, (DMSO-*d*_6_): *δ* 3.03 (s, 3H, methyl), 3.27 (s, 3H, methyl), 3.38 (s, 6H, methyl), 8.02 (s, 2H, aromatic); ^13^C NMR 100 MHz,(DMSO-*d*_6_): *δ* 35.1 (C-6), 37.6 (C-7), 42.8 (C-1), 130.8 (C-3), 132.3 (C-4), 132.7 (C-5), 152.8 (C-2); FT-IR (KBr): 3442 (medium, OH), 3026 (weak C-H), 1573 (medium C=C), 1314, 1341(strong S=O), 1149, 1126 (strong, S=O), 996, 812, 526, 497 cm^−1^; MS (EI, 70 eV): m/z (relative intensity): 357 (M^.^, 25), 278 (100), 79 (95), 199 (75), 120(52), 90(45).
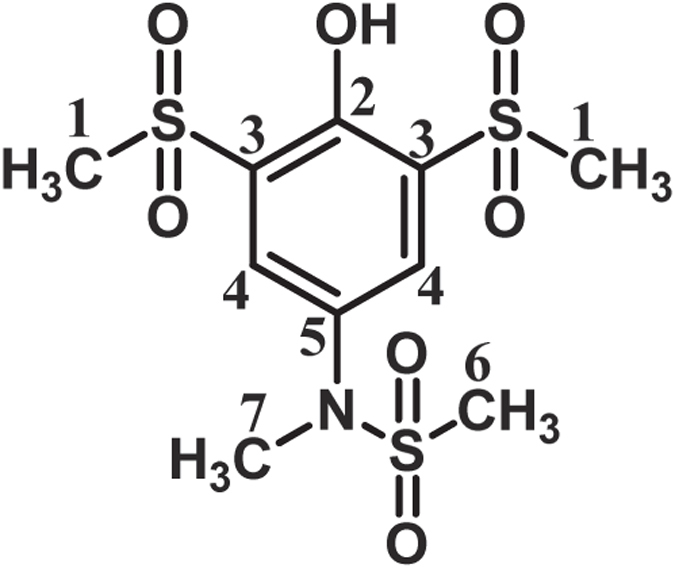


**Antibacterial Susceptibility Assay:** The samples were tested to determine the antibacterial susceptibility by Kirby-bauer disk diffusion method. *Escherichia coli* (*E. coli*) ATCC 35218 (gram negative) and *Staphylococcus aureus* (*S.aureus*) ATCC 6538 (gram positive) were used as test organisms. First, the colony of the bacterias of this study were cultured in the sterile Nutrient Broth and after 18–24 hours incubation at 37 °C microbial suspension was prepared from each bacteria balanced with turbusion 0.5 McFarland standard to 1.5 × 10^8^ cfu.ml^−1^ with distillated water and was cultured on Muller Hinton agar (MHA) (Merck, KGaA) culture condition in the form of lawn culture. To solve the present samples, dimethyl sulfoxide 10% in distillated water was used. Then 5 mg from each sample was poured on sterile blank discs, and after setting discs in aseptic conditions on cultured Muller Hinton, bacterias were incubated 24 hours at 37 °C in the presence of studied derivatives. Afterwards, the diameter of the inhabitation zone surrounding of the discs were measured by special ruler. Also, to study the lack of influence of the solution containing 10% dimethyl sulfoxide on the bacterias of the study, 20 microlitre of the solution was poured on the discs and set on cultured Muller Hinton agar and incubated.

### Digital Simulation

Digital simulation was performed using the DigiElch SB simulation software version 2.0^37^. The simulation was carried out assuming semi-infinite one-dimensional diffusion and planar electrode geometry. The experimental parameters entered for digital simulation consisted of the following: (i) the transfer coefficient (α) was assumed to be 0.5. (ii) The formal potentials were obtained experimentally as the midpoint potential between the anodic and cathodic peaks (*E*_mid_). (iii) Analytical concentration of **MAP**, *E*_start_, *E*_switch_, *E*_end_ and temperature = 25 °C. All these parameters were kept constant throughout the fitting of the digitally simulated voltammogram to the experimental data. Electrochemical oxidation of **MAP** was examined by digital simulation in the pH range 1–6. The simulation was performed based on the proposed *EC* and *ECEC* mechanisms (for simplifying, the proton transfers processes are not included). In these mechanisms, *E* is electrochemical oxidation of **MAP** to **MAP**_**OX**_, and *C* is the addition reaction that happens after the electrooxidation process. In these mechanisms, the peak current ratio (*I*_pC_/*I*_pA1_) is a criterion for the chemical reaction rate between **MAP**_**OX**_ and sulfinic ion nucleophiles.

## Electronic supplementary material

Supplementary information
