# Supplementary material for: A green strategy for the synthesis of sulfone derivatives of p-methylaminophenol: Kinetic evaluation and antibacterial susceptibility
Source: Sci Rep. 2017 Jun 30;7:4436. doi: 10.1038/s41598-017-04581-0 (PMC5493611; doi:10.1038/s41598-017-04581-0)

Supplementary Information

A green strategy for the synthesis of sulfone derivatives of *p*-methylaminophenol. Kinetic evaluation and antibacterial Susceptibility

Davood Nematollahi,* Sadegh Khazalpour, Mina Ranjbar & Shima Momeni

Faculty of Chemistry, Bu-Ali-Sina University, Hamedan 65174, Iran.

nemat@basu.ac.ir.

Table of Contents:

1.	Antibacterial susceptibility assay	Page IV
2.	Digital simulation	Page VIII
3.	FT-IR spectrum of MSP1	Page XV
4.	^{1}H NMR spectrum of MSP1	Page XVI
5.	^{13}C NMR spectrum of MSP1	Page XVII
6.	MS spectrum of MSP1	Page XVIII
7.	FT-IR spectrum of MSP2	Page XIX
8.	^{1}H NMR spectrum of MSP2	Page XX
9.	^{13}C NMR spectrum of MSP2	Page XXI
10.	MS spectrum of MSP2	Page XXII
11.	FT-IR spectrum of MSP3	Page XXIII
12.	^{1}H NMR spectrum of MSP3	Page XXIV
13.	^{13}C NMR spectrum of MSP3	Page XXV
14.	MS spectrum of MSP3	Page XXVI
15.	FT-IR spectrum of MSP4	Page XXVII
16.	^{1}H NMR spectrum of MSP4	Page XXVIII
17.	^{13}C NMR spectrum of MSP4	Page XXIX
18.	MS spectrum of MSP4	Page XXX
19.	FT-IR spectrum of BSP1	Page XXXI
20	^{1}H NMR spectrum of BSP1	Page XXXII
21.	^{13}C NMR spectrum of BSP1	Page XXXIII
22.	MS spectrum of BSP1	Page XXXIV
23.	FT-IR spectrum of BSP2	Page XXXV
24.	^{1}H NMR spectrum of BSP2	Page XXXVI
25.	^{13}C NMR spectrum of BSP2	Page XXXVII
26.	MS spectrum of BSP2	Page XXXVIII

27. **FT-IR spectrum of BSP3** **Page XXXIX**

28. **^{1}H NMR spectrum of BSP3** **Page XL**

29. **^{13}C NMR spectrum of BSP3** **Page XLI**

30. **MS spectrum of BSP3** **Page XLII**

31. **FT-IR spectrum of TSP** **Page XLIII**

32. **^{1}H NMR spectrum of TSP** **Page XLIV**

33. **^{13}C NMR spectrum of TSP** **Page XLV**

34. **MS spectrum of TSP** **Page XLVI**

Antibacterial susceptibility assay

Table 1. Antibacterial activity products **MSP1-4.**

Strain	**MSP1**	**MSP2**	**MSP3**	**MSP4**
Staphylococcus aureus	20 mm	22 mm	13 mm	30 mm
Escherichia coli	7 mm	8 mm	7 mm	13 mm

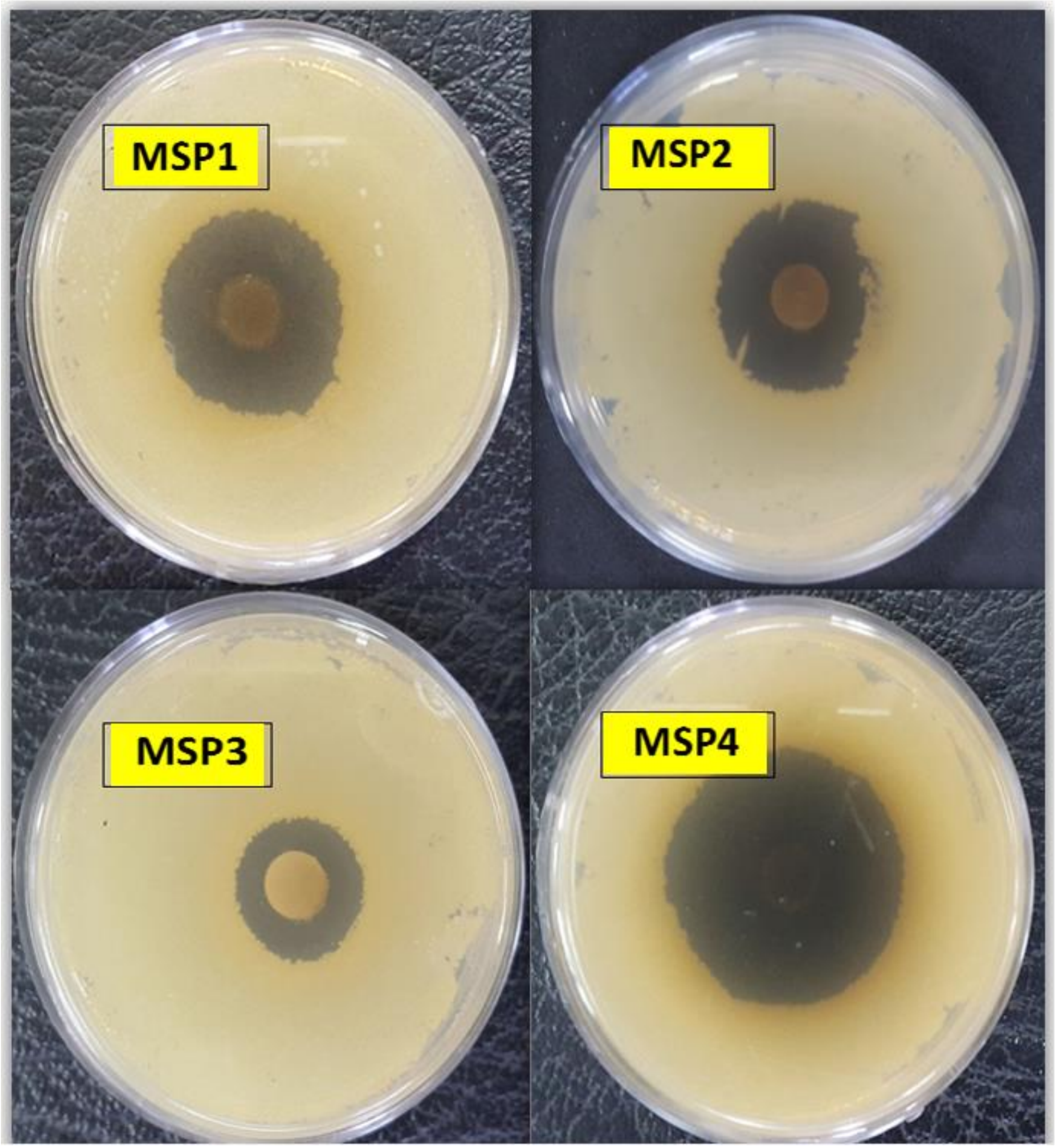

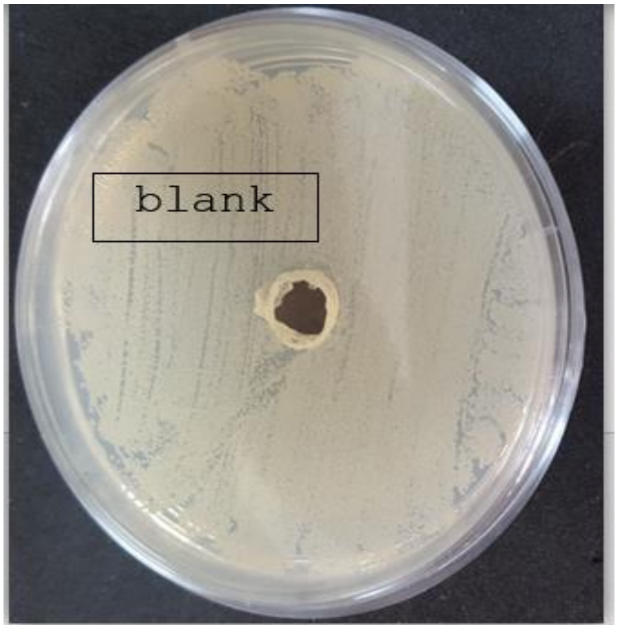

Figure S1. Inhibition zone diameters (mm) obtained of *Staphylococcus aureus* in disc diffusion test for **MSP1-4** (5 mg) and solvent.

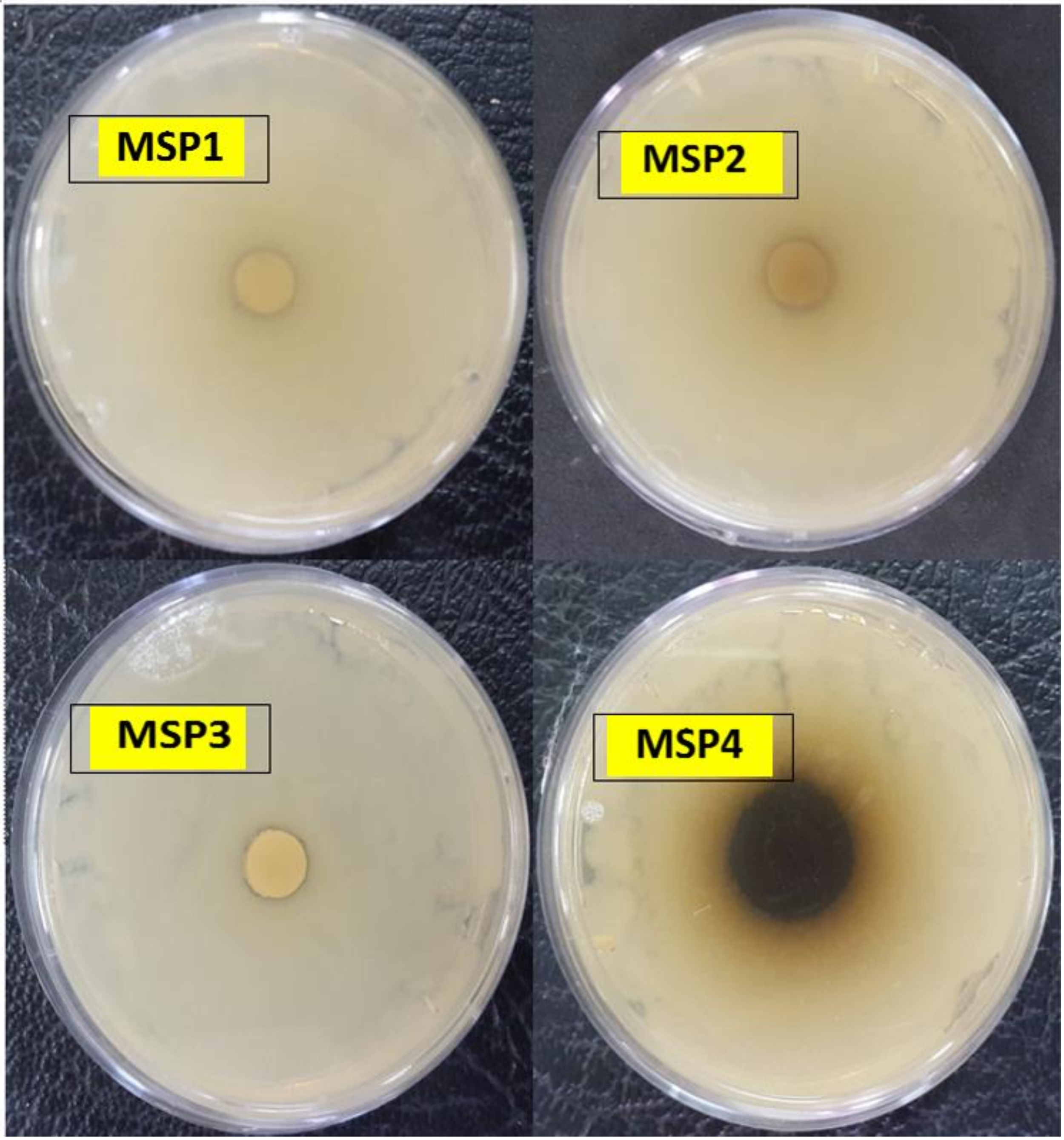
MSP1
MSP2
MSP3
MSP4

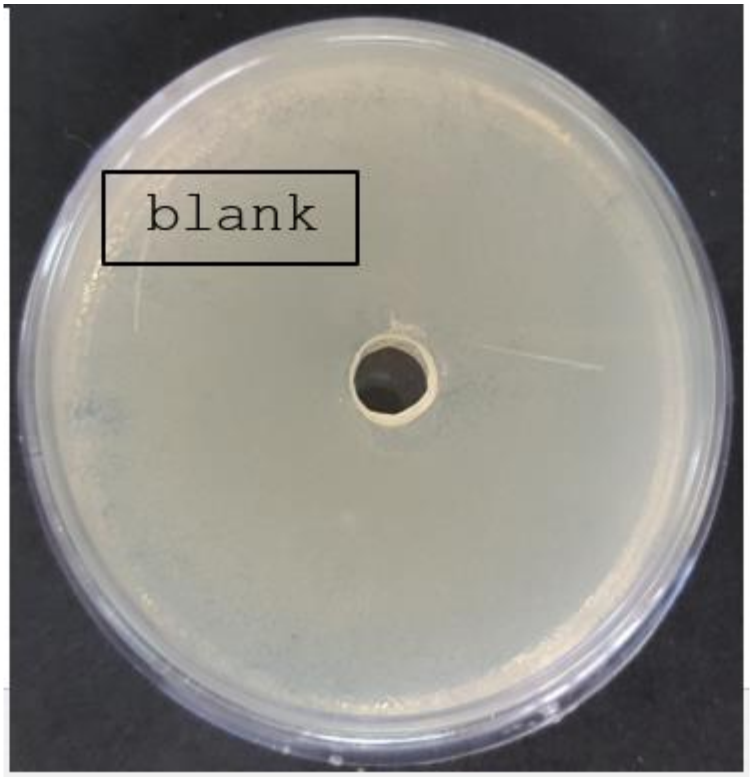

Figure S2. Inhibition zone diameters (mm) obtained of *Escherichia coli* in disc diffusion test for **MSP1-4** (5 mg) and solvent.

Digital simulation

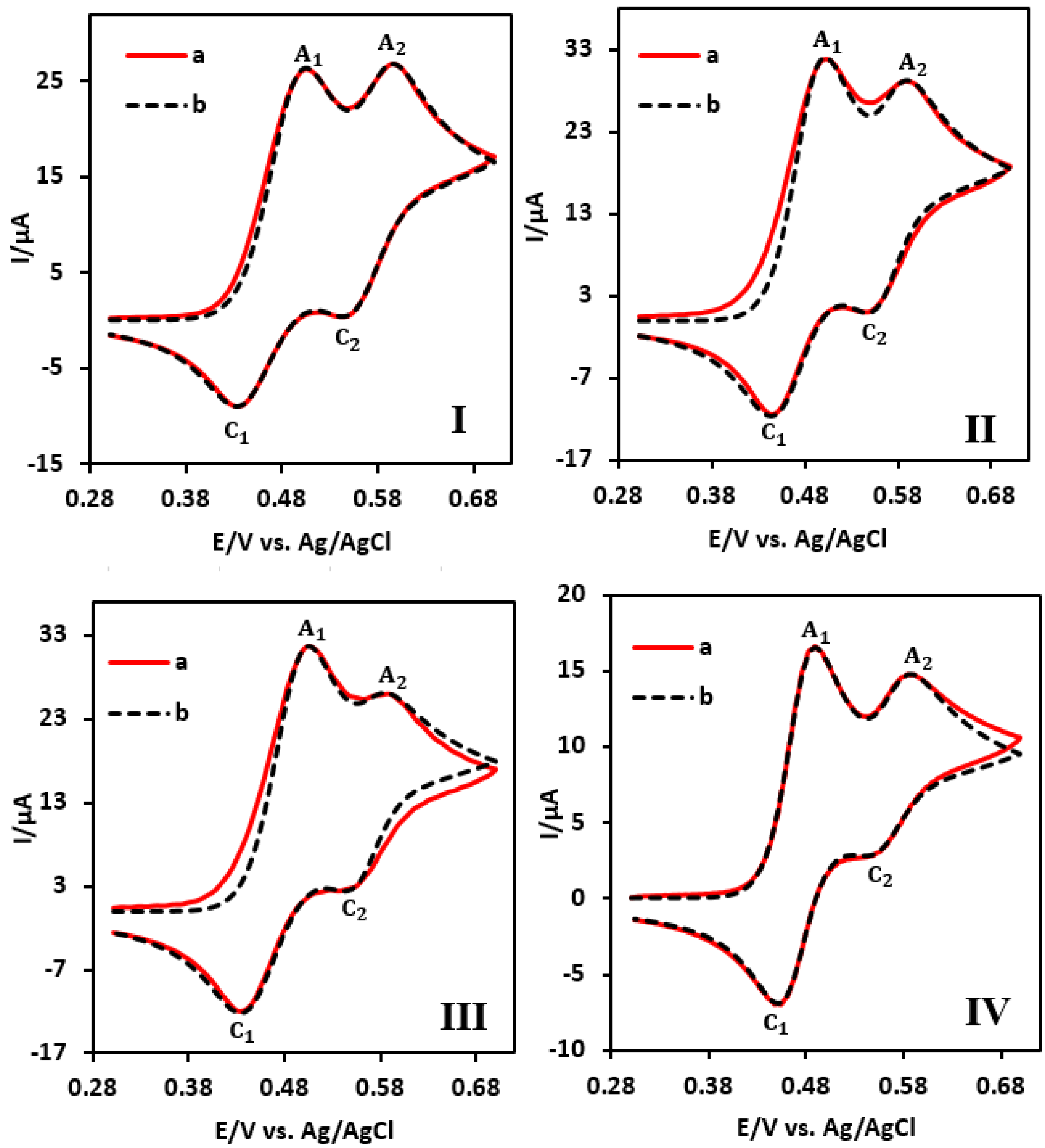

Figure S3. Experimental (a) and simulated (b) cyclic voltammograms of **MAP** (1 mM) in the presence of (I) *p*-toluenesulfinic acid (0.5 mM), (II) benzensulfinic acid (0.5 mM), (III) *p*-chlorosulfinic acid (0.5 mM) and (IV) methanesulfinic acid (0.5 mM) at glassy carbon electrode in aqueous $HClO_4$ (0.1 M). Scan rate: 80 mV/s. Temperature = 25 ± 1 °C.

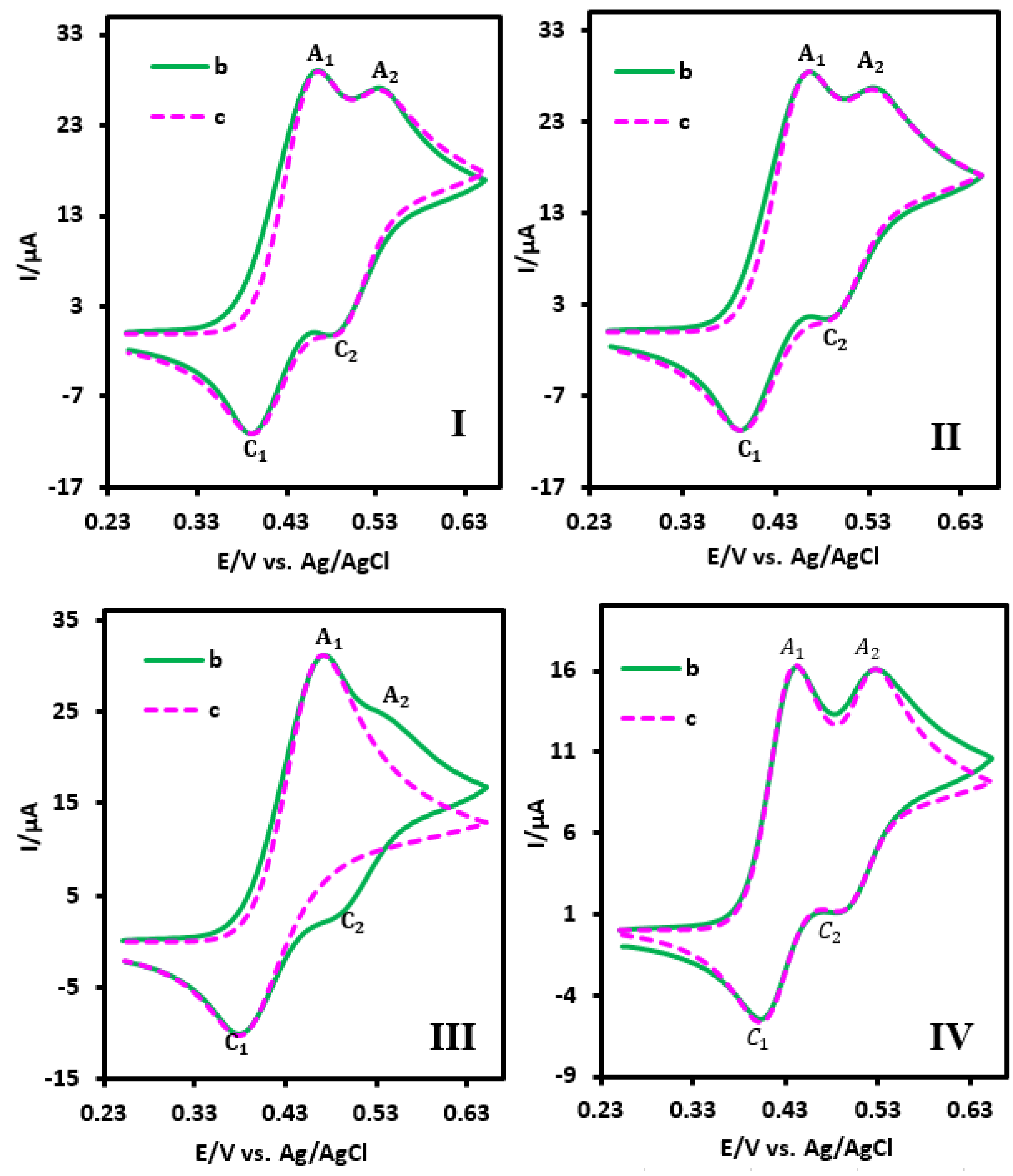

Figure S4. Experimental (b) and simulated (c) cyclic voltammograms of **MAP** (1 mM) in the presence of (I) *p*-toluenesulfinic acid (0.5 mM), (II) benzensulfinic acid (0.5 mM), (III) *p*-chlorosulfinic acid (0.5 mM) and (IV) methanesulfinic acid (0.5 mM) at glassy carbon electrode in aqueous phosphate buffer (pH = 1.8, *c* = 0.2 M). Scan rate: 70 mV/s. Temperature = 25 ± 1 ^{o}C.

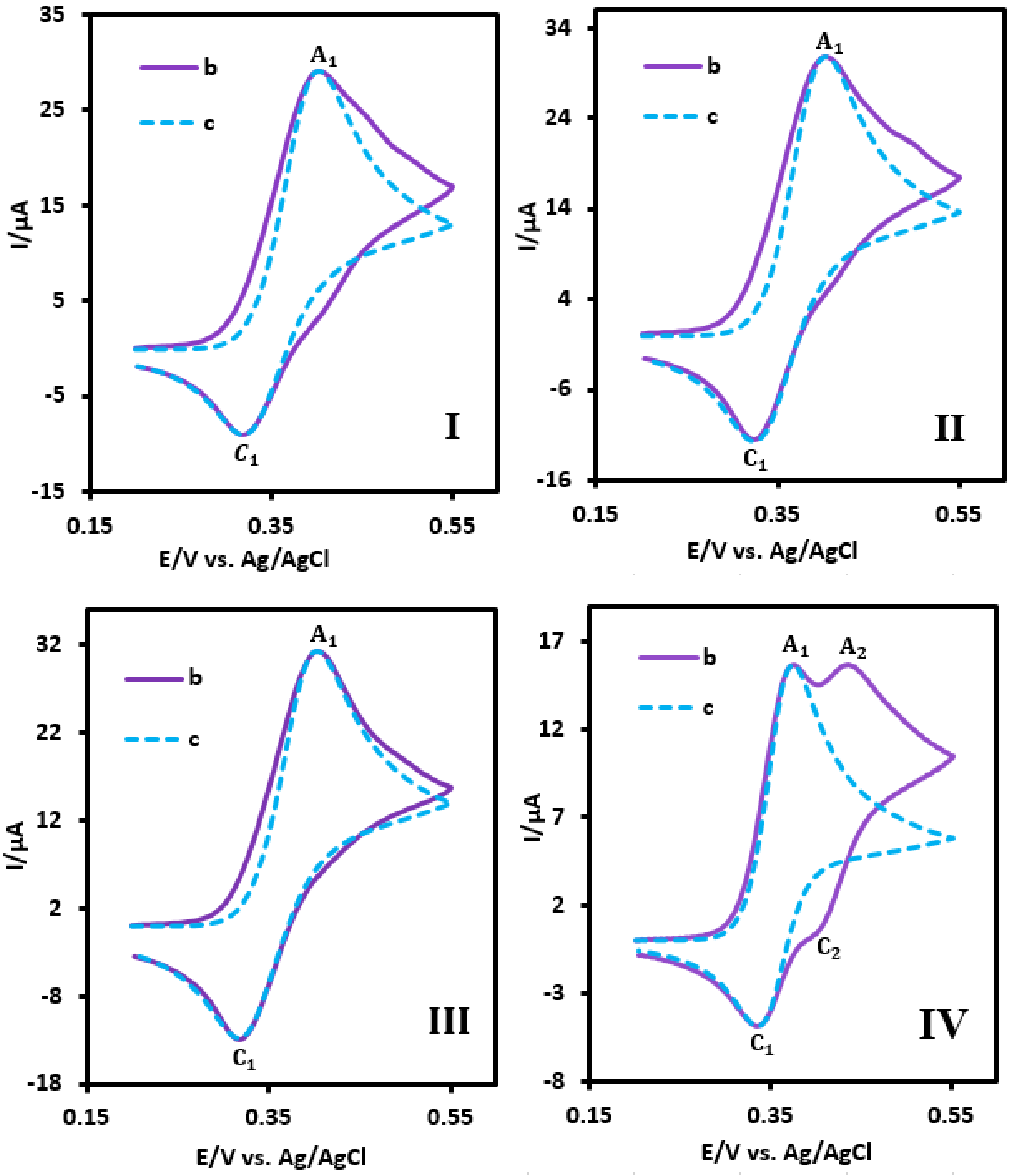

Figure S5. Experimental (b) and simulated (c) cyclic voltammograms of **MAP** (1 mM) in the presence of (I) *p*-toluenesulfinic acid (0.5 mM), (II) benzensulfinic acid (0.5 mM), (III) *p*-chlorosulfinic acid (0.5 mM) and (IV) methanesulfinic acid (0.5 mM) at glassy carbon electrode in aqueous phosphate buffer (pH = 3.0, *c* = 0.2 M). Scan rate: 70 mV/s. Temperature = 25 ± 1 ^{o}C.

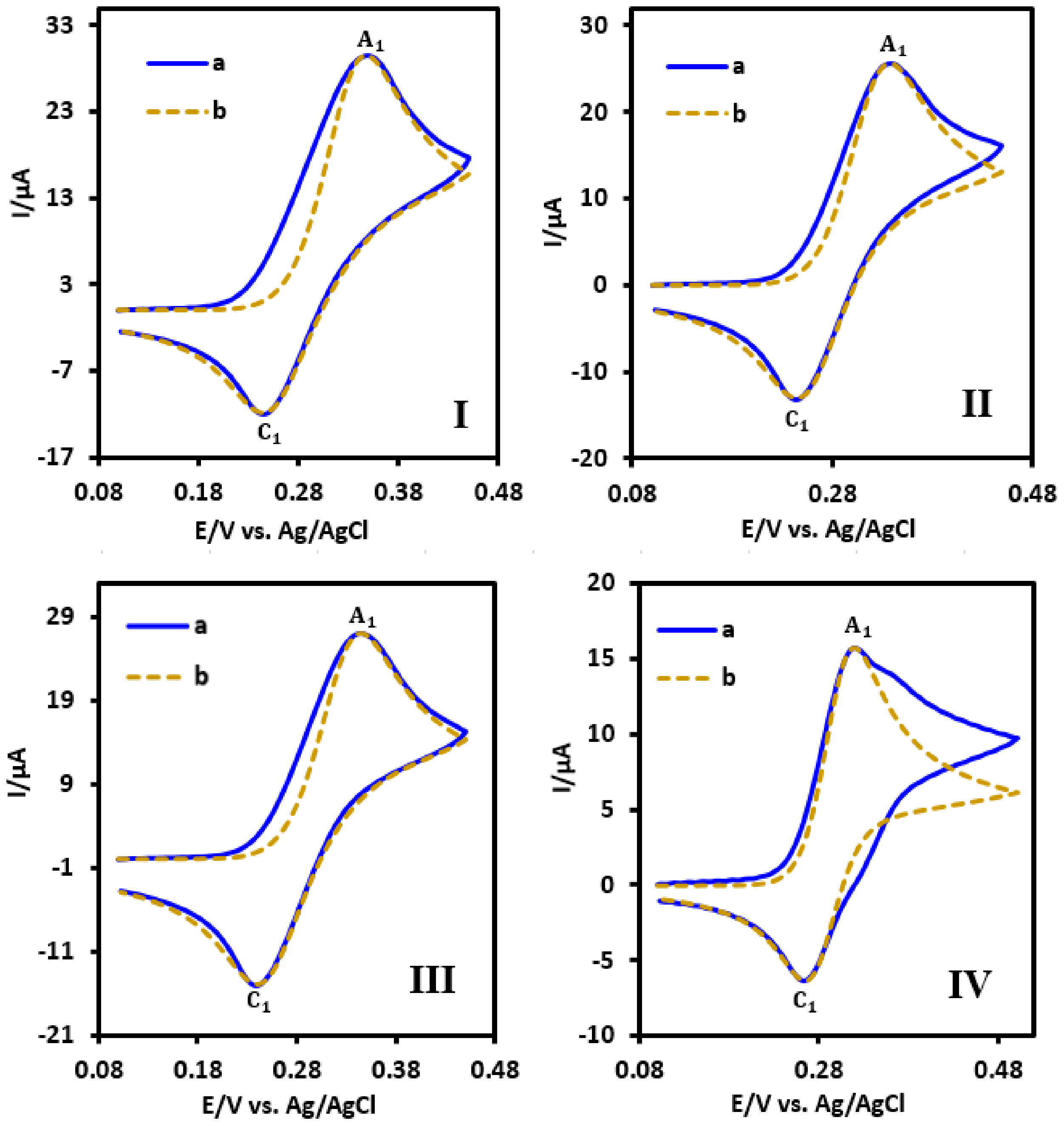

Figure S6. Experimental (a) and simulated (b) cyclic voltammograms of **MAP** (1 mM) in the presence of (I) *p*-toluenesulfinic acid (0.5 mM), (II) benzensulfinic acid (0.5 mM), (III) *p*-chlorosulfinic acid (0.5 mM) and (IV) methanesulfinic acid (0.5 mM) at glassy carbon electrode in aqueous acetate buffer (pH = 4.0, *c* = 0.2 M). Scan rate: 60 mV/s. Temperature = 25 ± 1 °C.

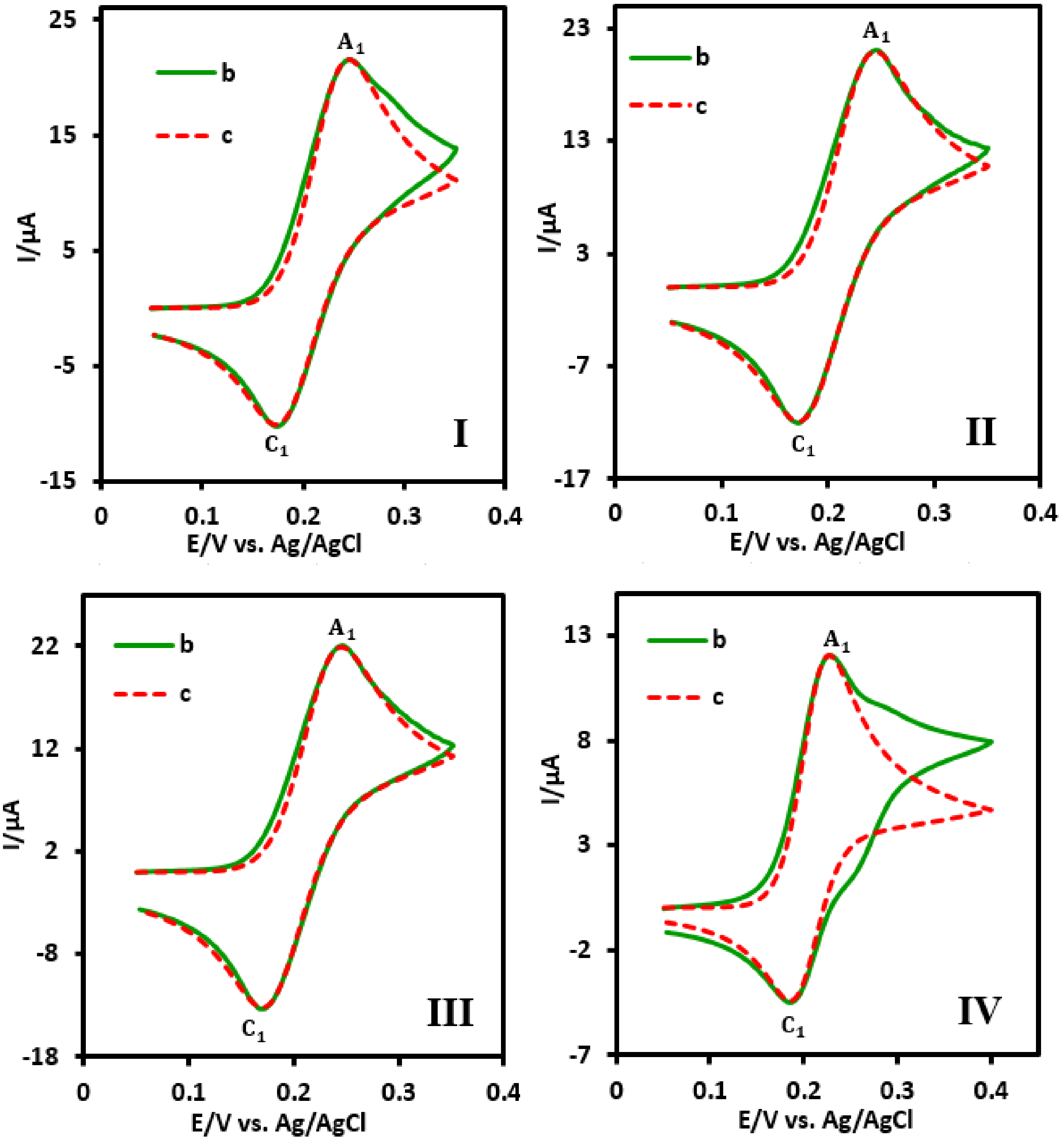

Figure S7. Experimental (a) and simulated (b) cyclic voltammograms of **MAP** (1 mM) in the presence of (I) *p*-toluenesulfinic acid (0.5 mM), (II) benzensulfinic acid (0.5 mM), (III) *p*-chlorosulfinic acid (0.5 mM) and (IV) methanesulfinic acid (0.5 mM) at glassy carbon electrode in aqueous acetate buffer (pH = 5.0, *c* = 0.2 M). Scan rate: 40 mV/s. Temperature = 25 ± 1 °C.

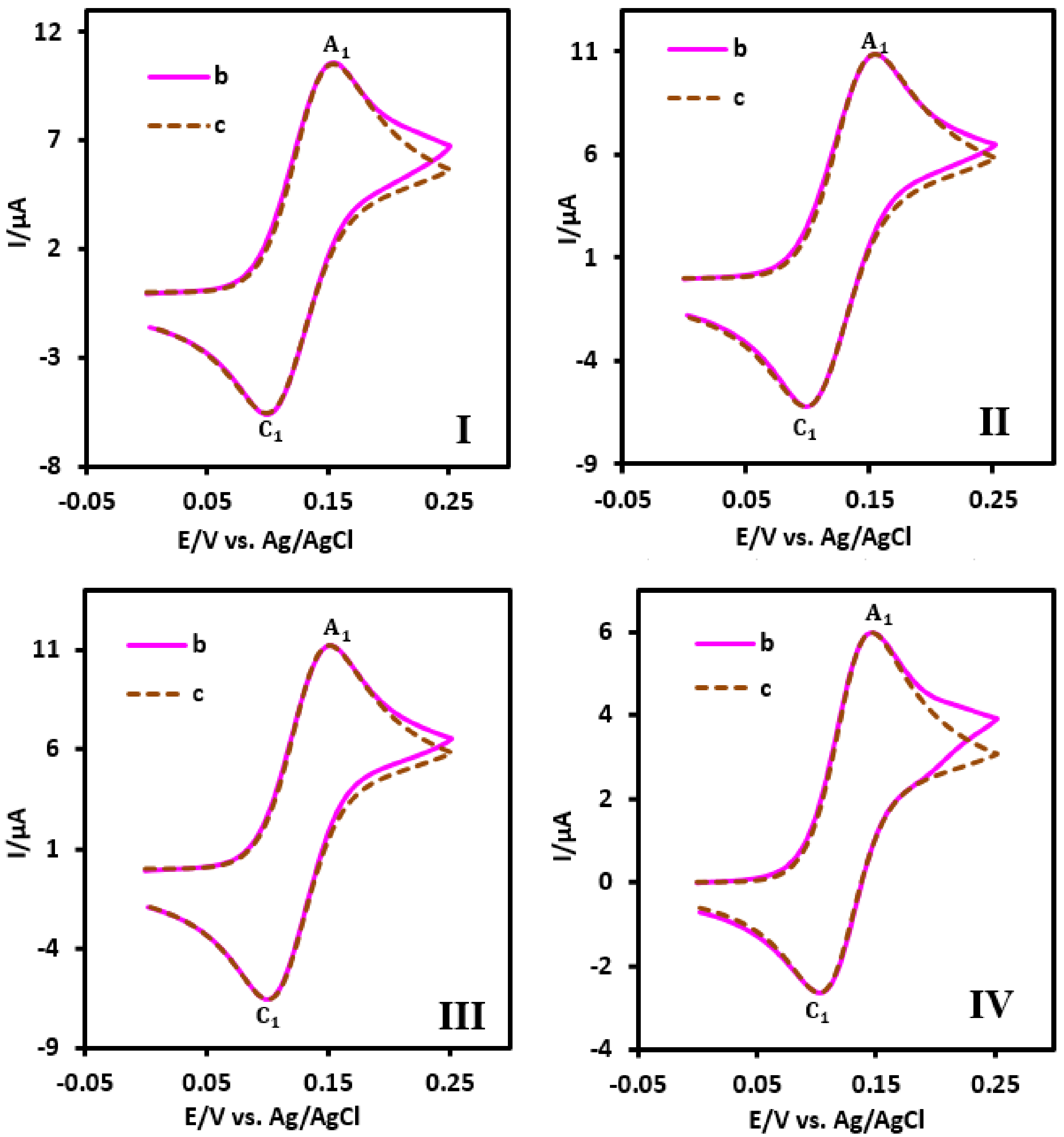

Figure S8. Experimental (a) and simulated (b) cyclic voltammograms of **MAP** (1 mM) in the presence of (I) *p*-toluenesulfinic acid (0.5 mM), (II) benzensulfinic acid (0.5 mM), (III) *p*-chlorosulfinic acid (0.5 mM) and (IV) methanesulfinic acid (0.5 mM) at glassy carbon electrode in aqueous phosphate buffer (pH = 6.0, *c* = 0.2 M). Scan rate: 10 mV/s. Temperature = 25 ± 1 ^{o}C.

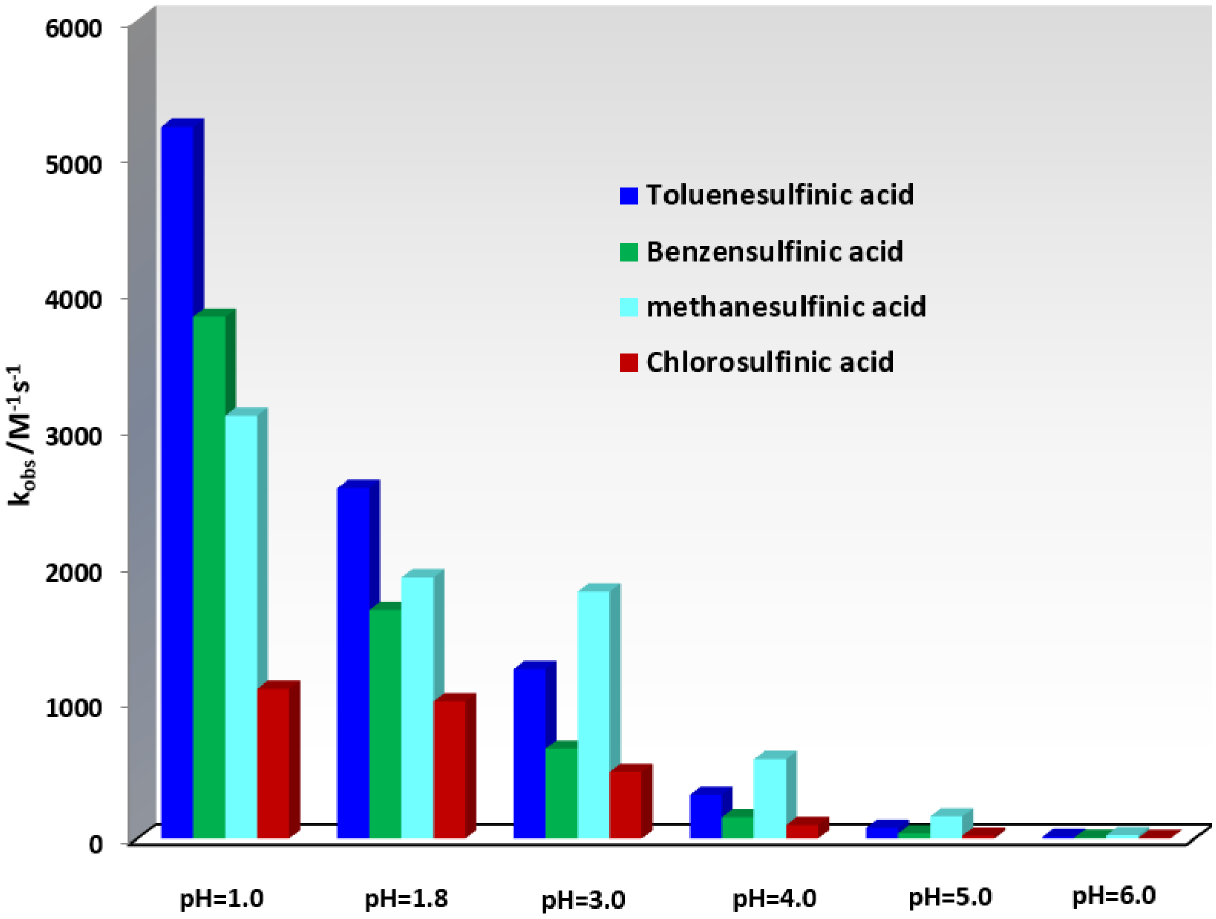

Figure S9. Observed homogeneous rate constants (k_{obs}) of the reaction of **MQI** with the sulfone nucleophiles at different pH values.

IR spectrum of MSP1

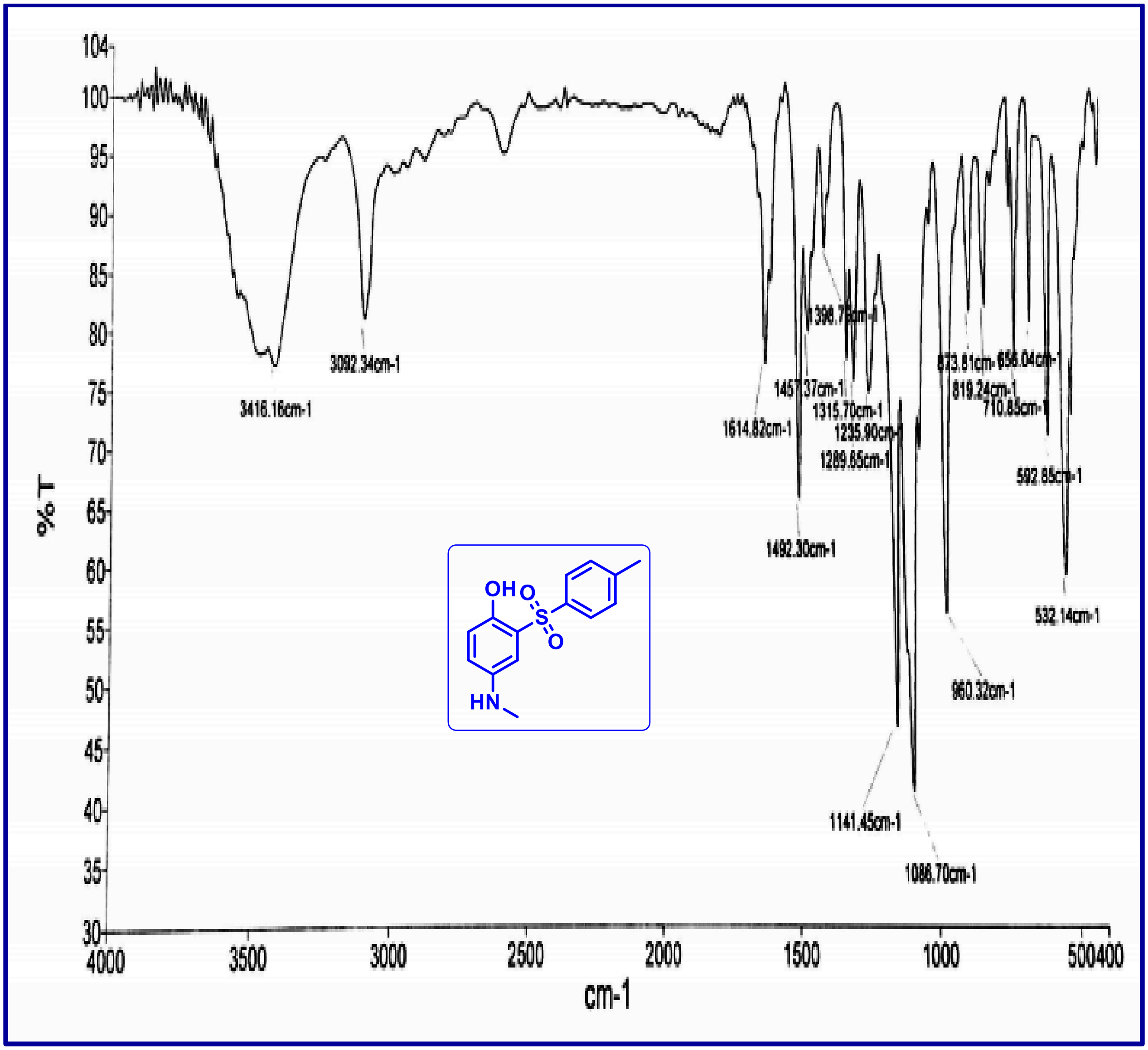

^{1}H NMR spectrum of MSP1

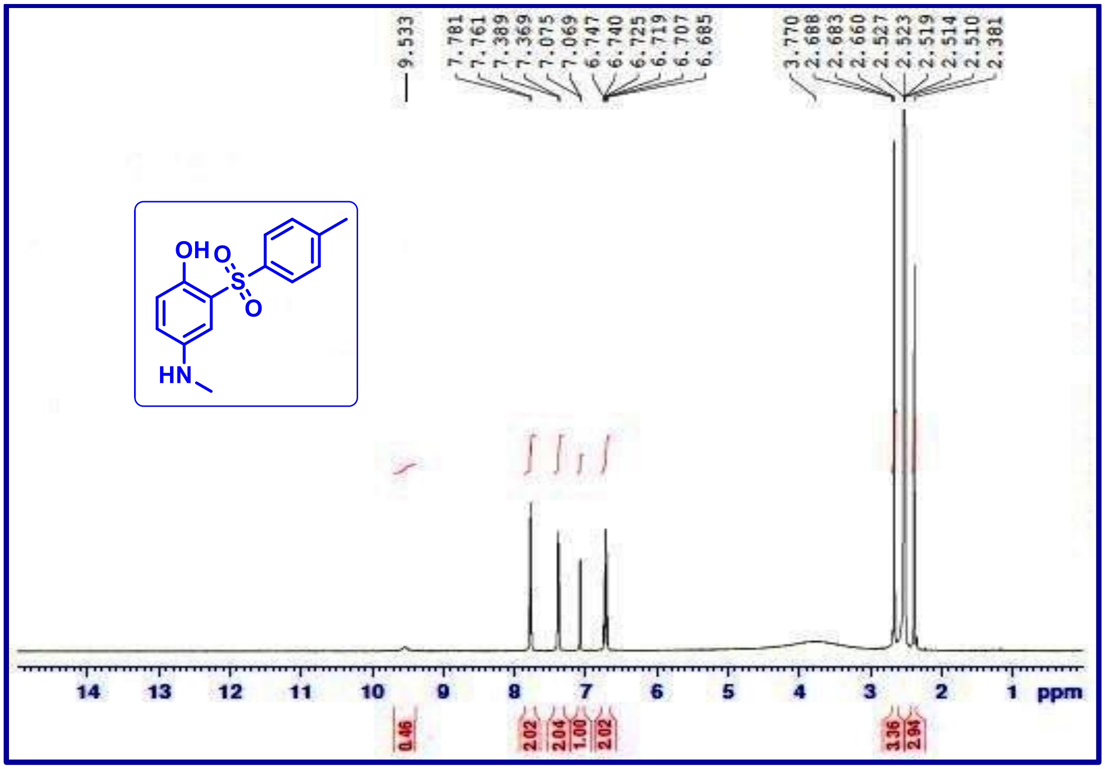

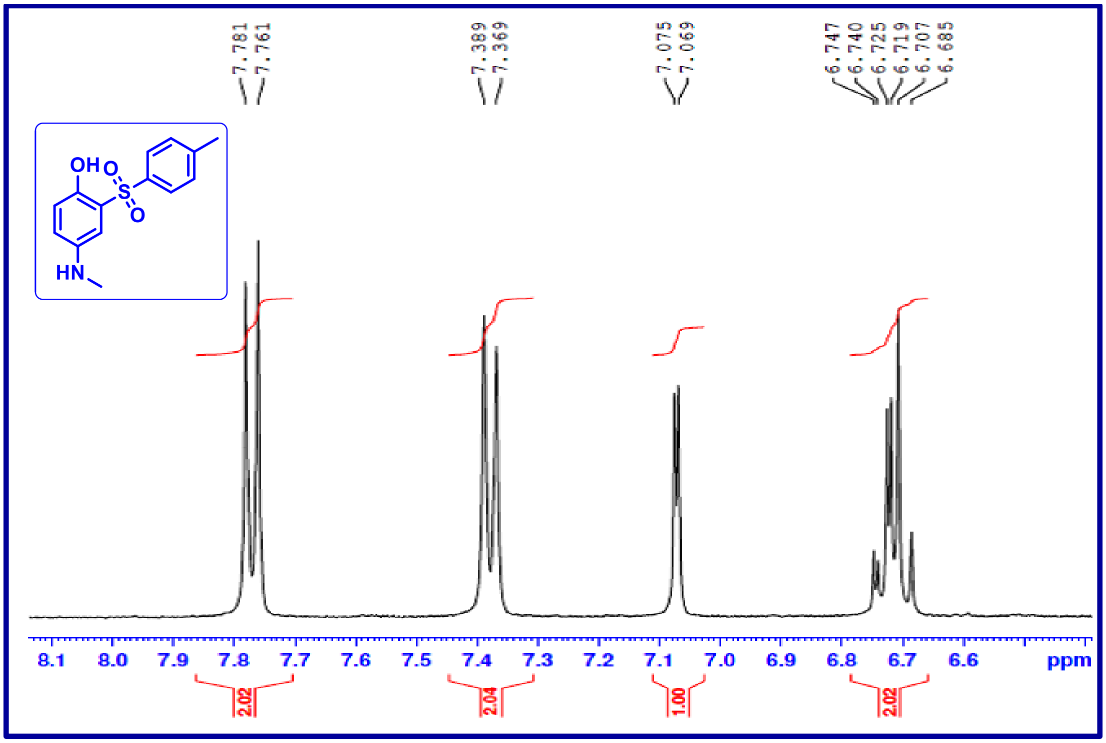

^{13}C NMR spectrum of MSP1

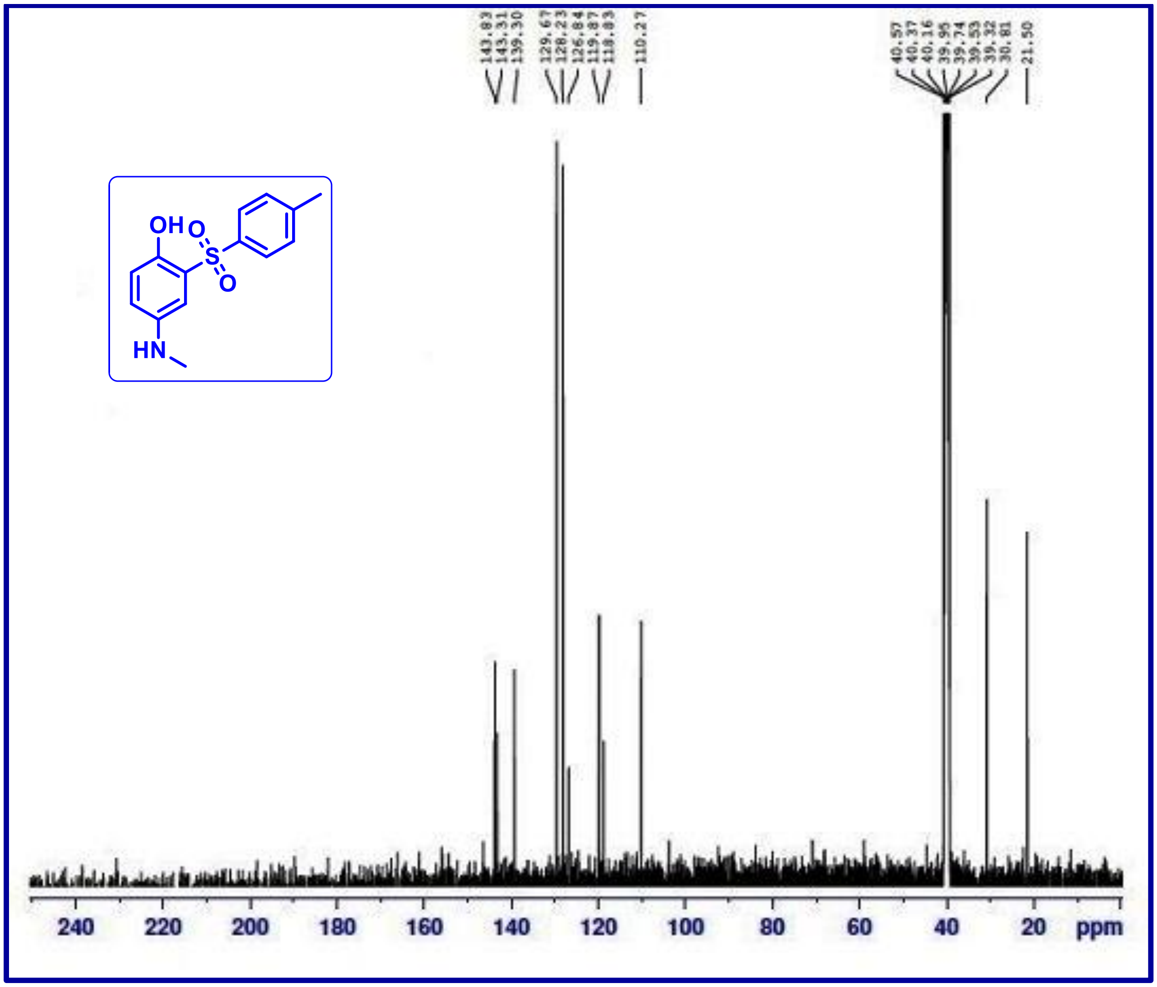

Mass spectrum of MSP1

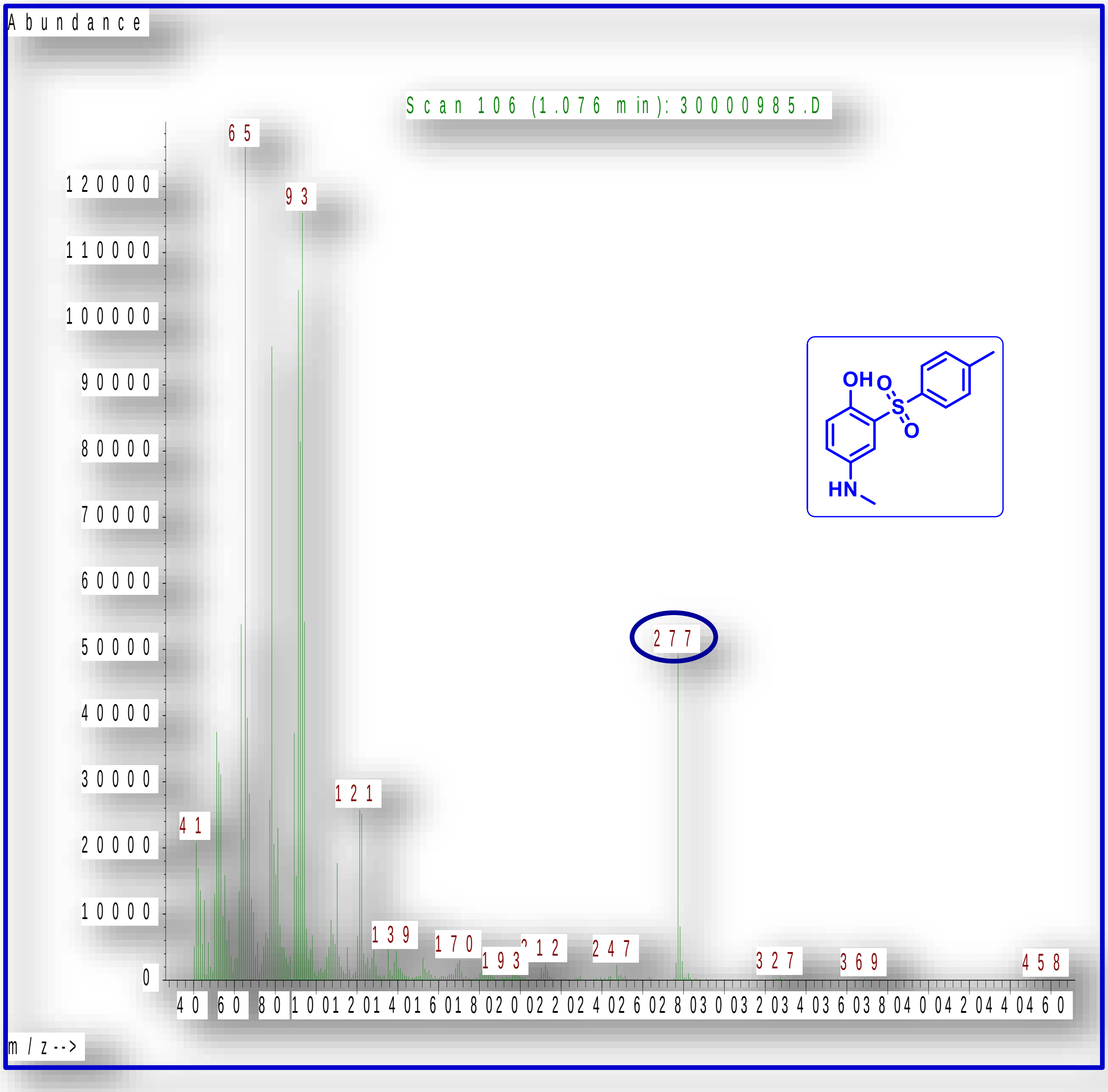

IR spectrum of MSP2

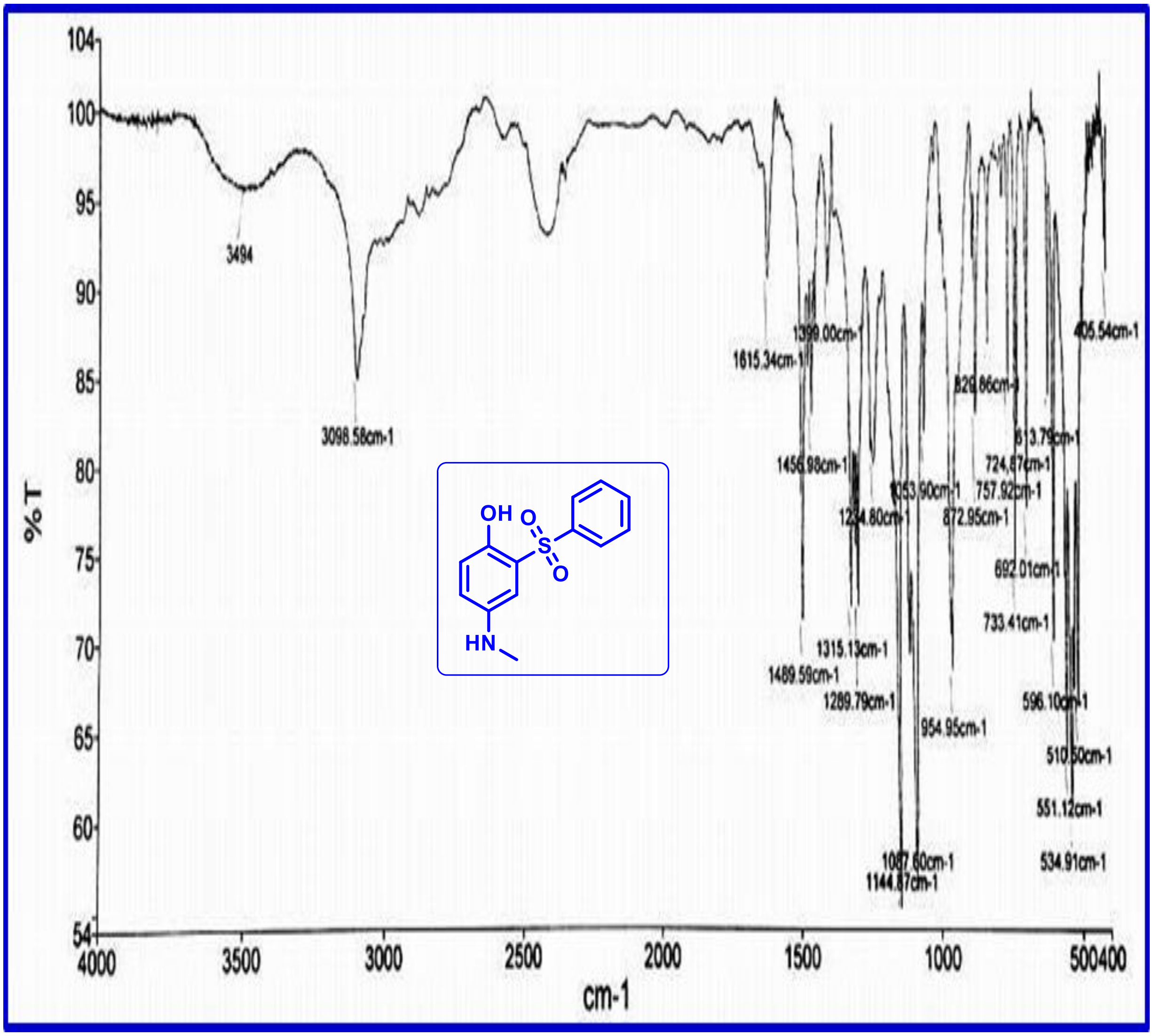

^{1}H NMR spectrum of MSP2

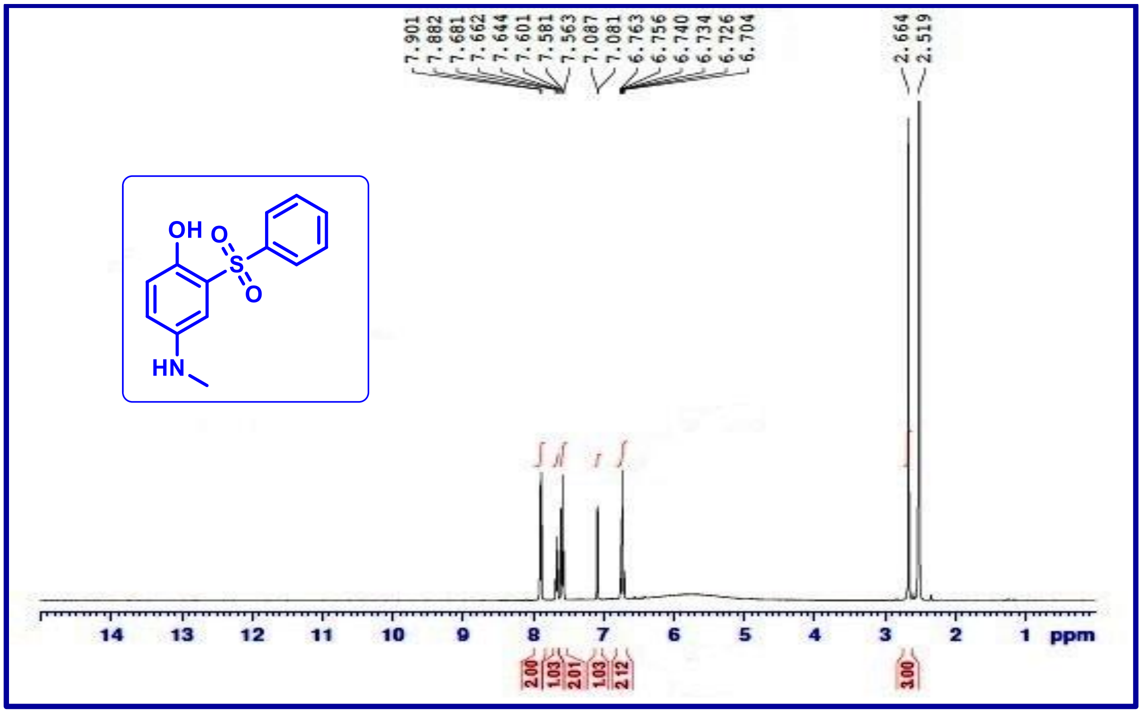

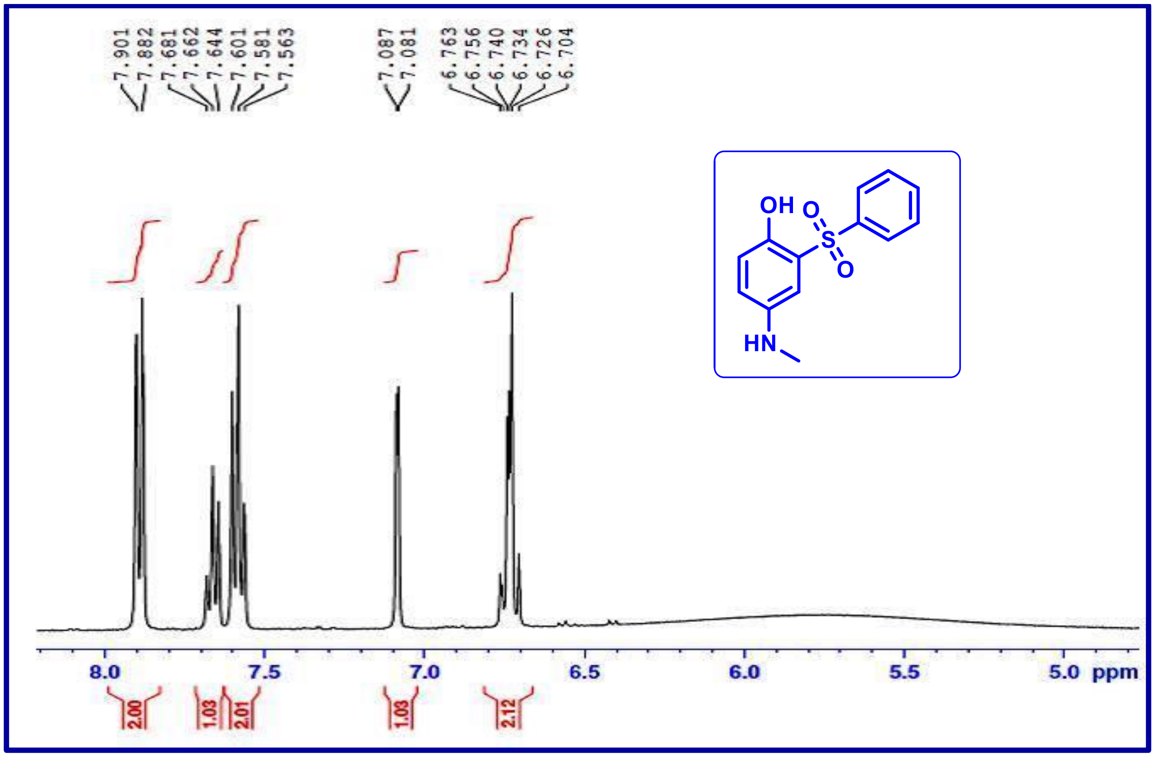

^{13}C NMR spectrum of MSP2

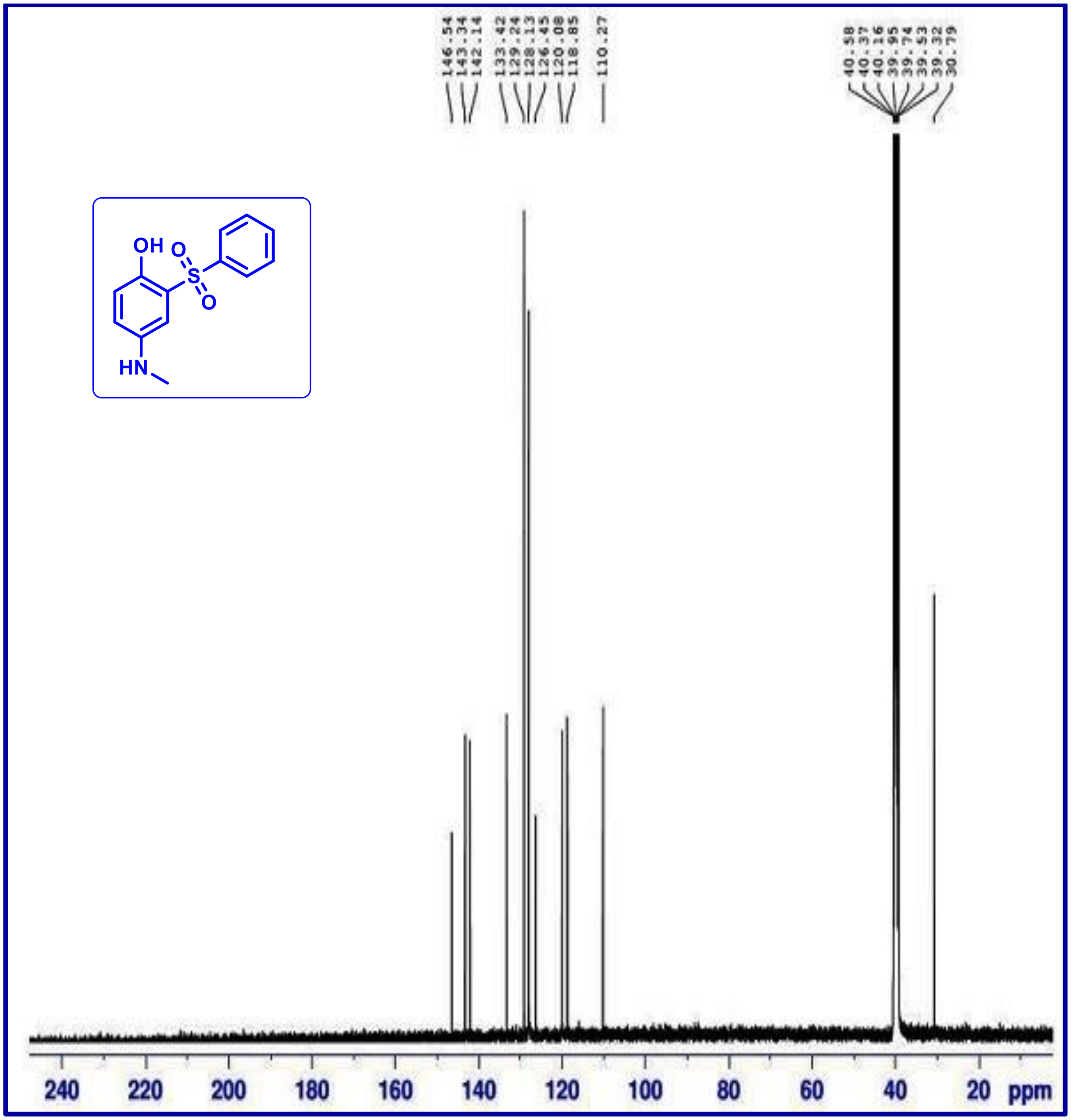

Mass spectrum of MSP2

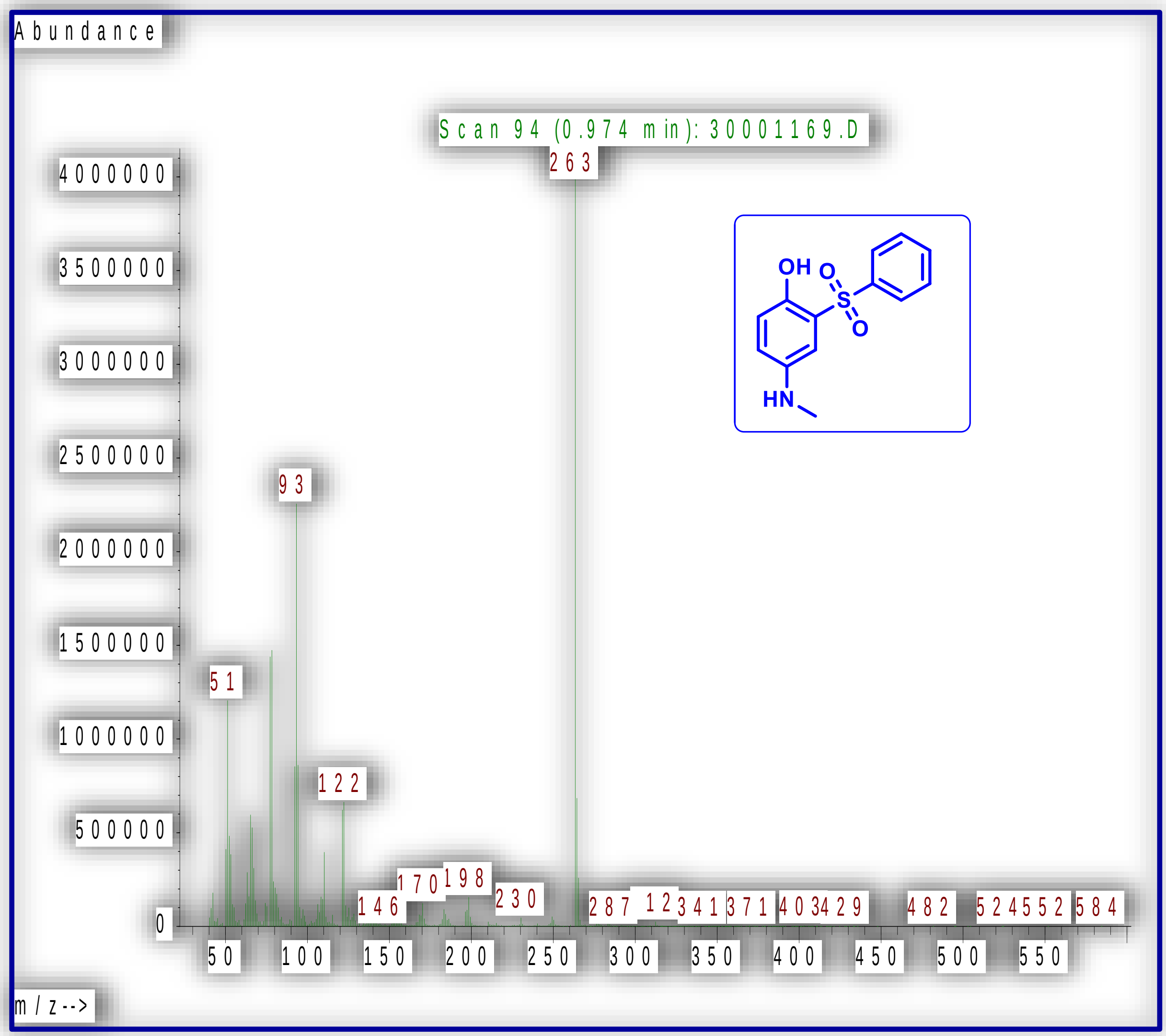

IR spectrum of MSP3

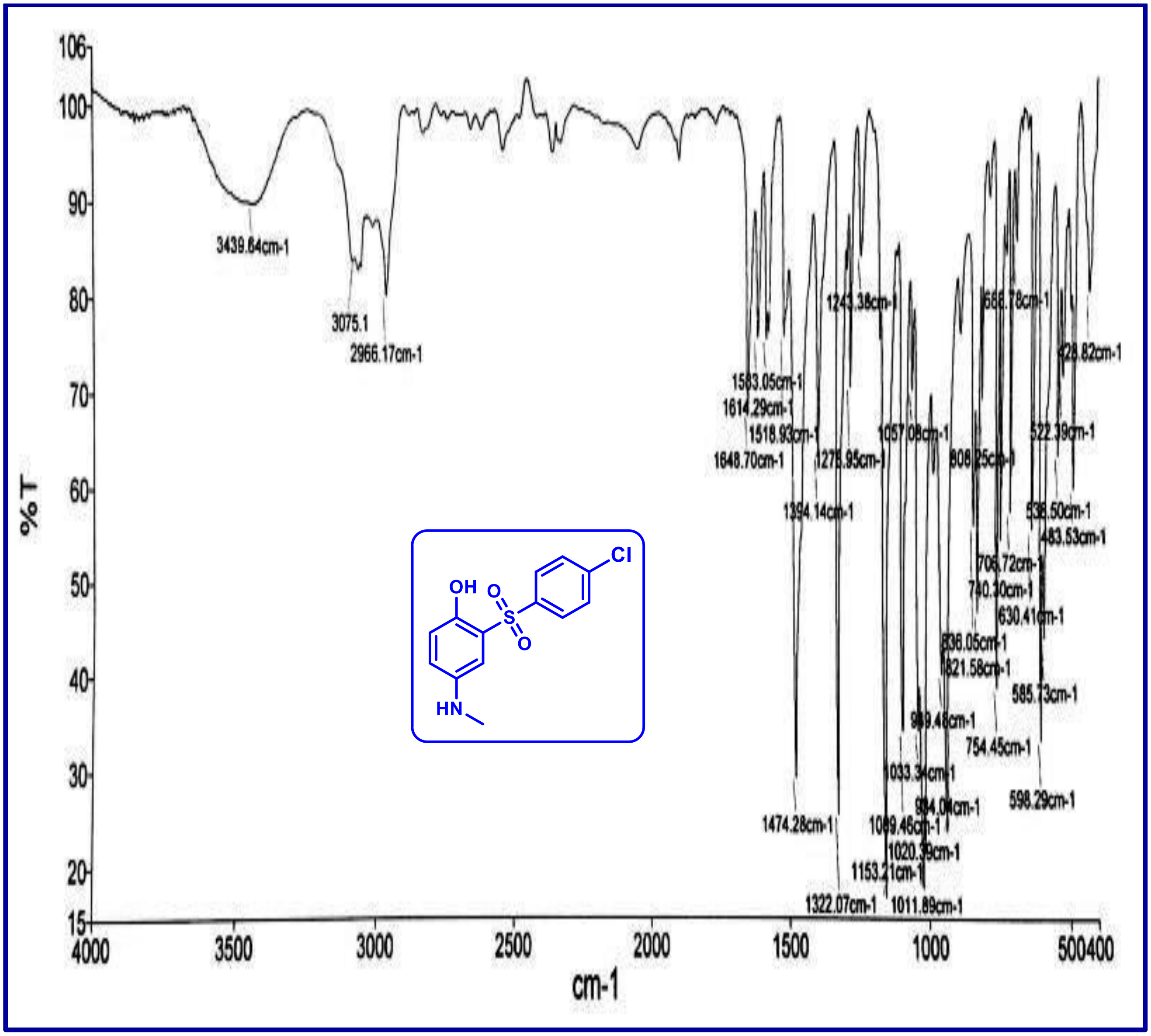

^{1}H NMR spectrum of MSP3

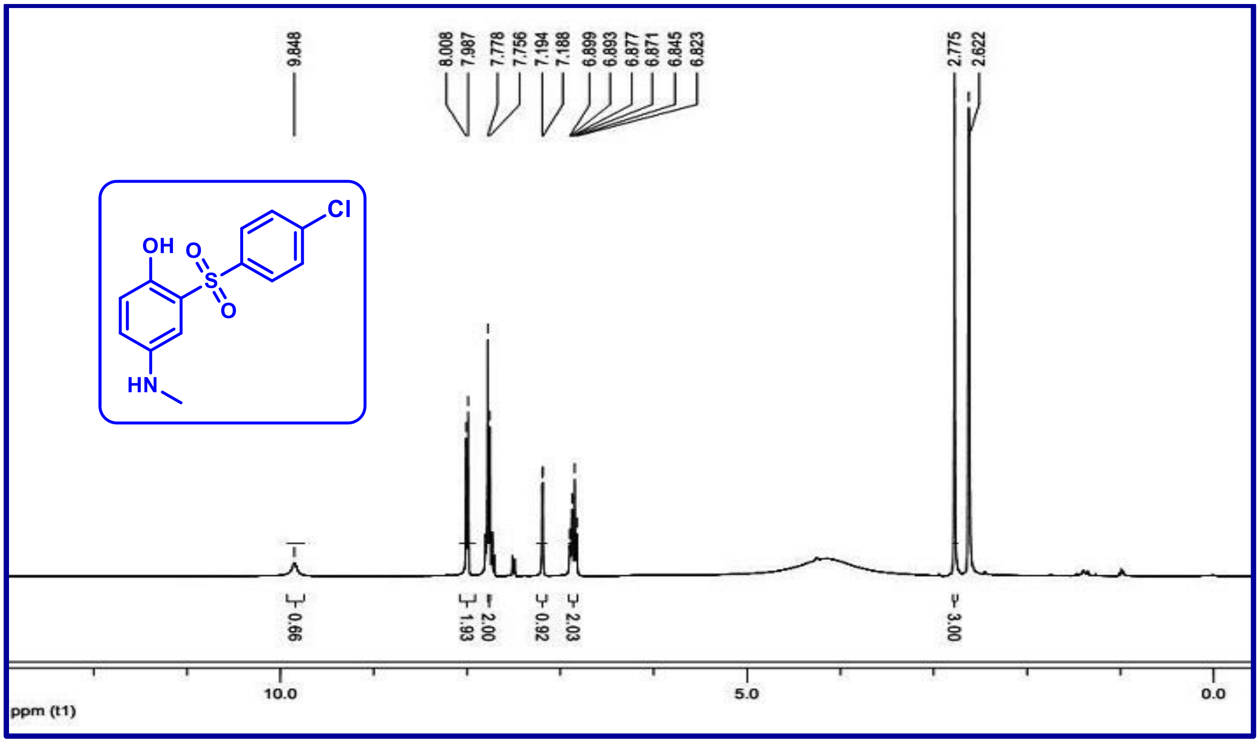

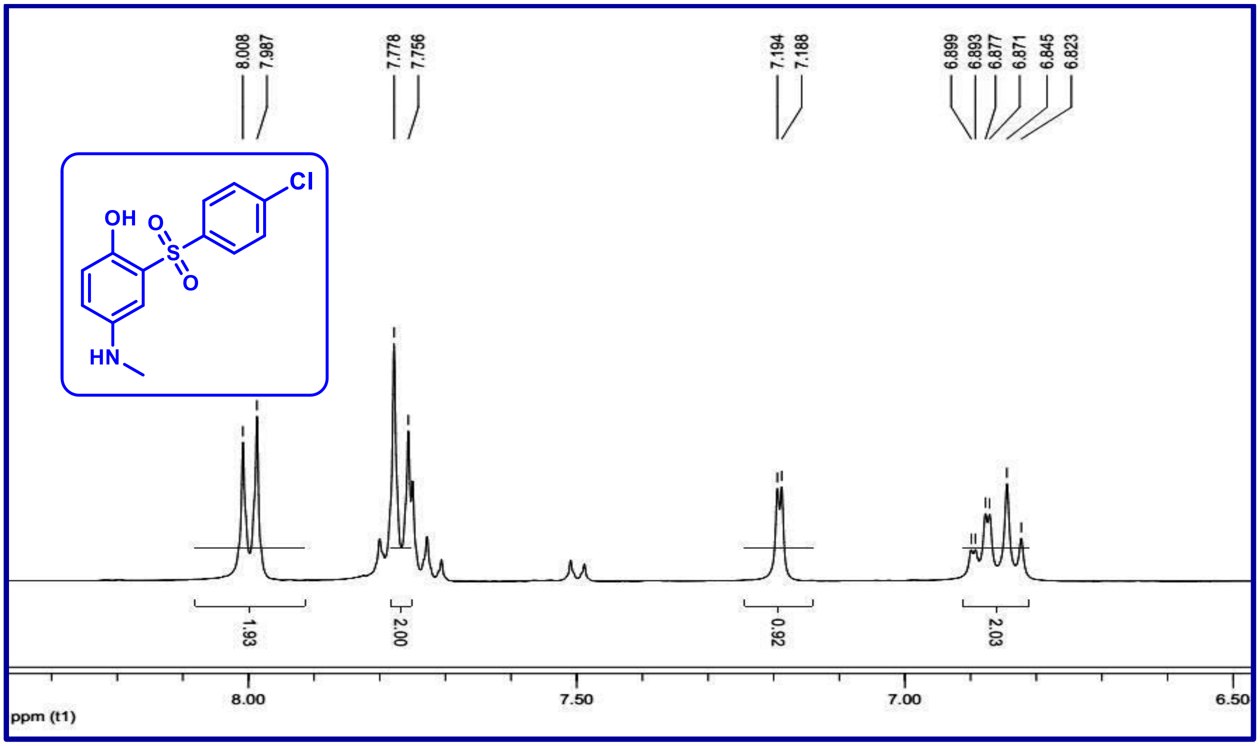

^{13}CNMR spectrum of MSP3

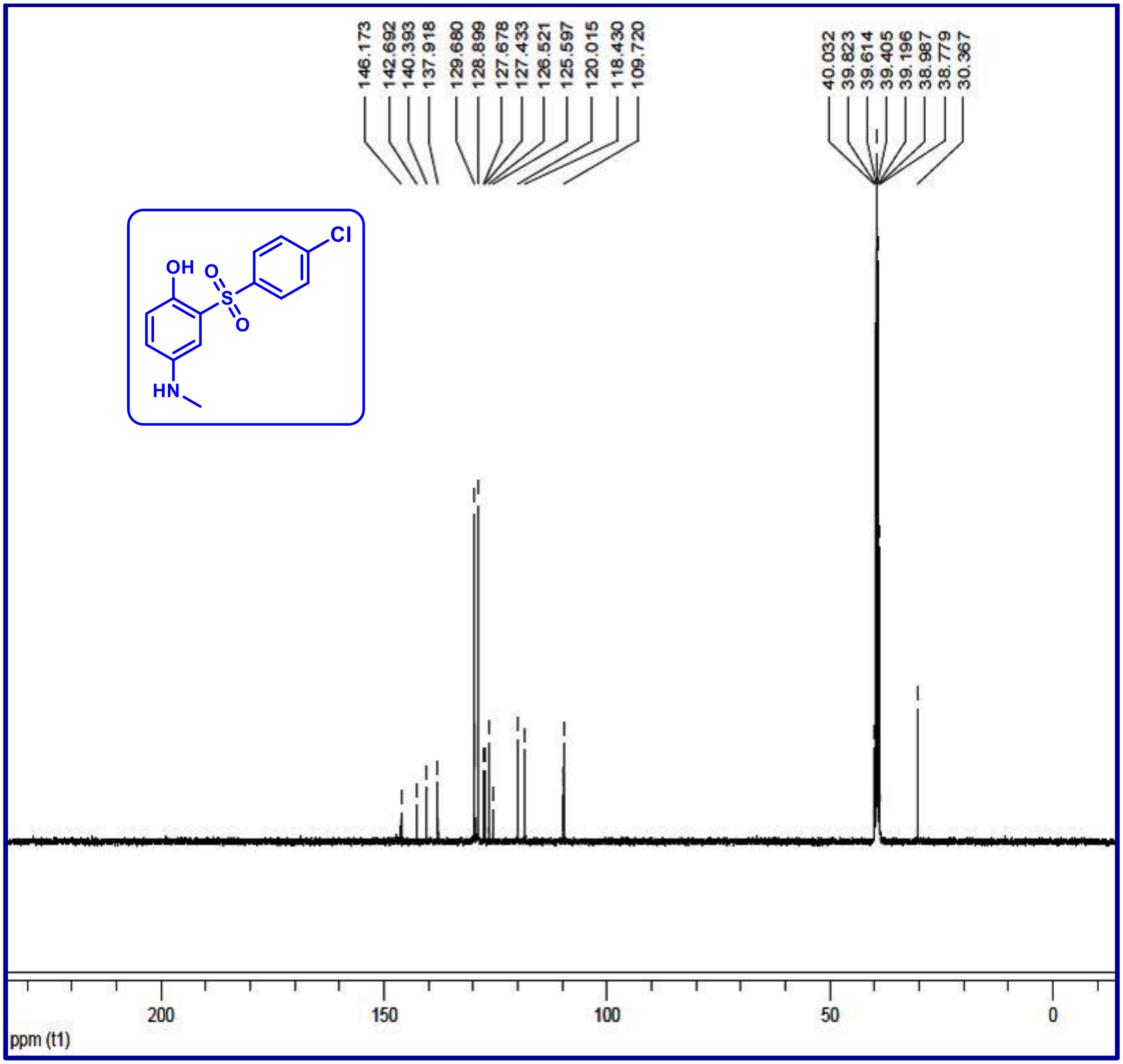

Mass spectrum of MSP3

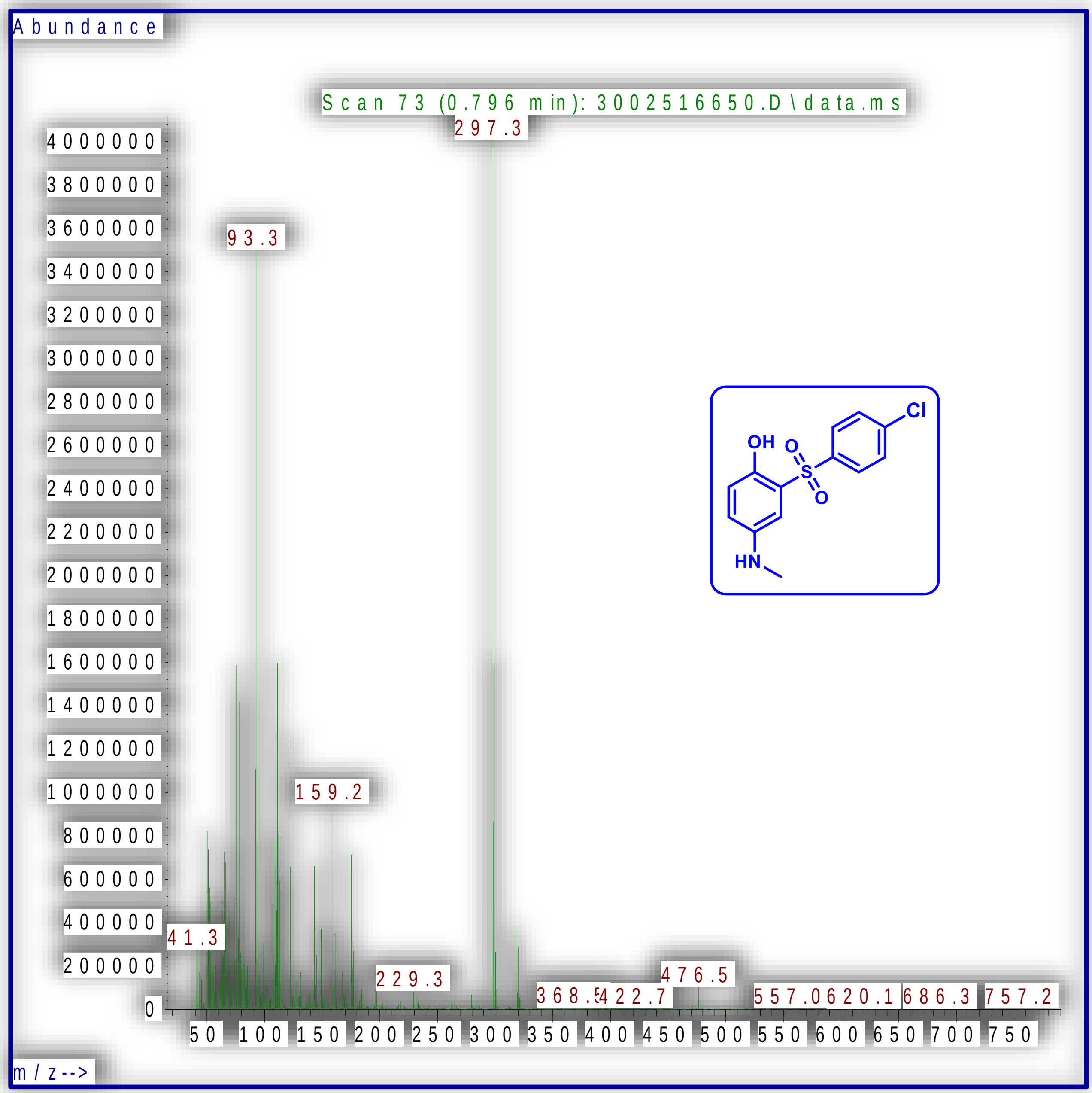

IR spectrum of MSP4

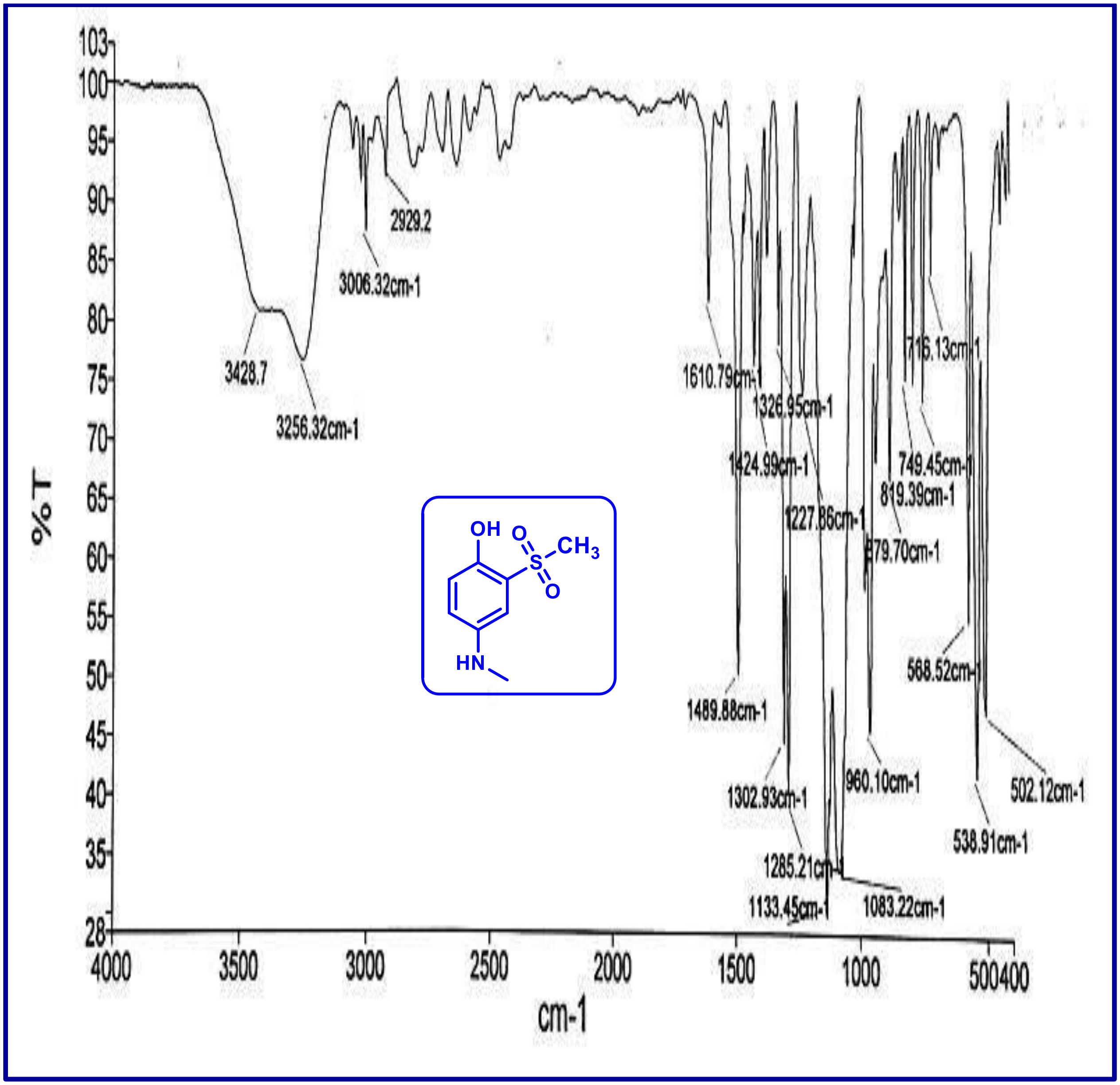

^{1}H NMR spectrum of MSP4

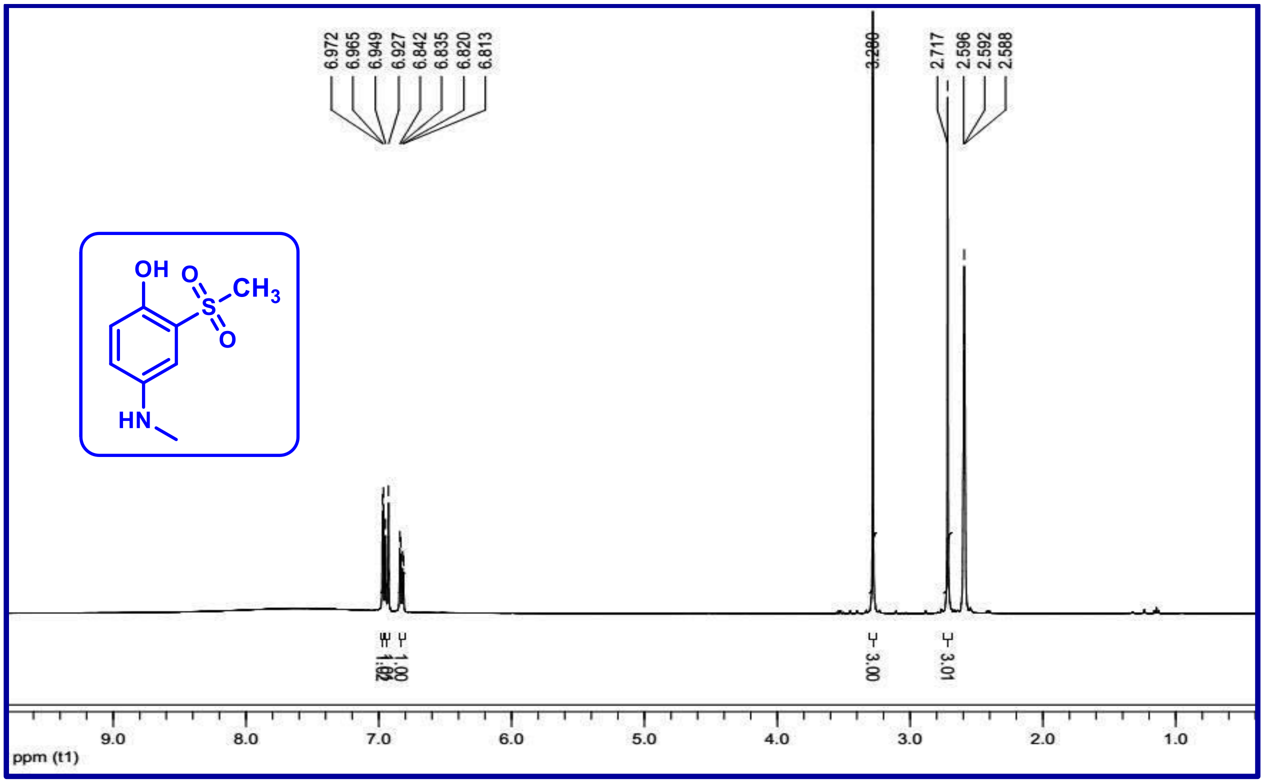

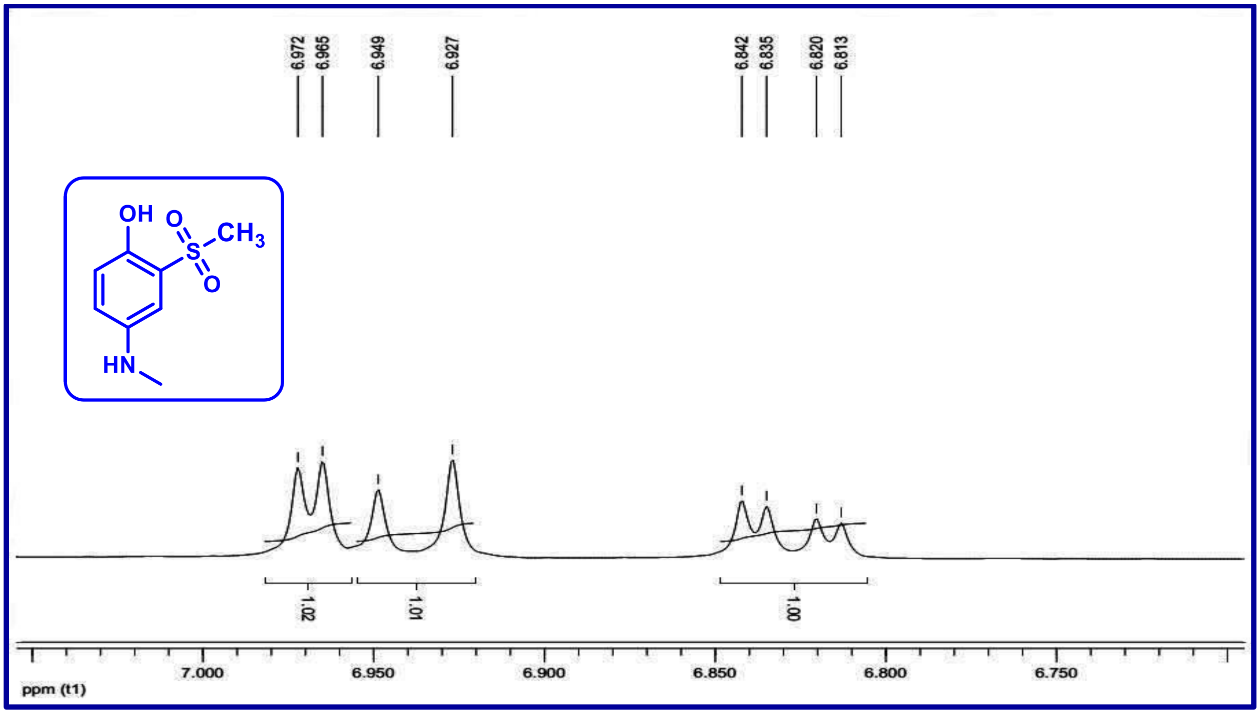

^{13}C NMR spectrum of MSP4

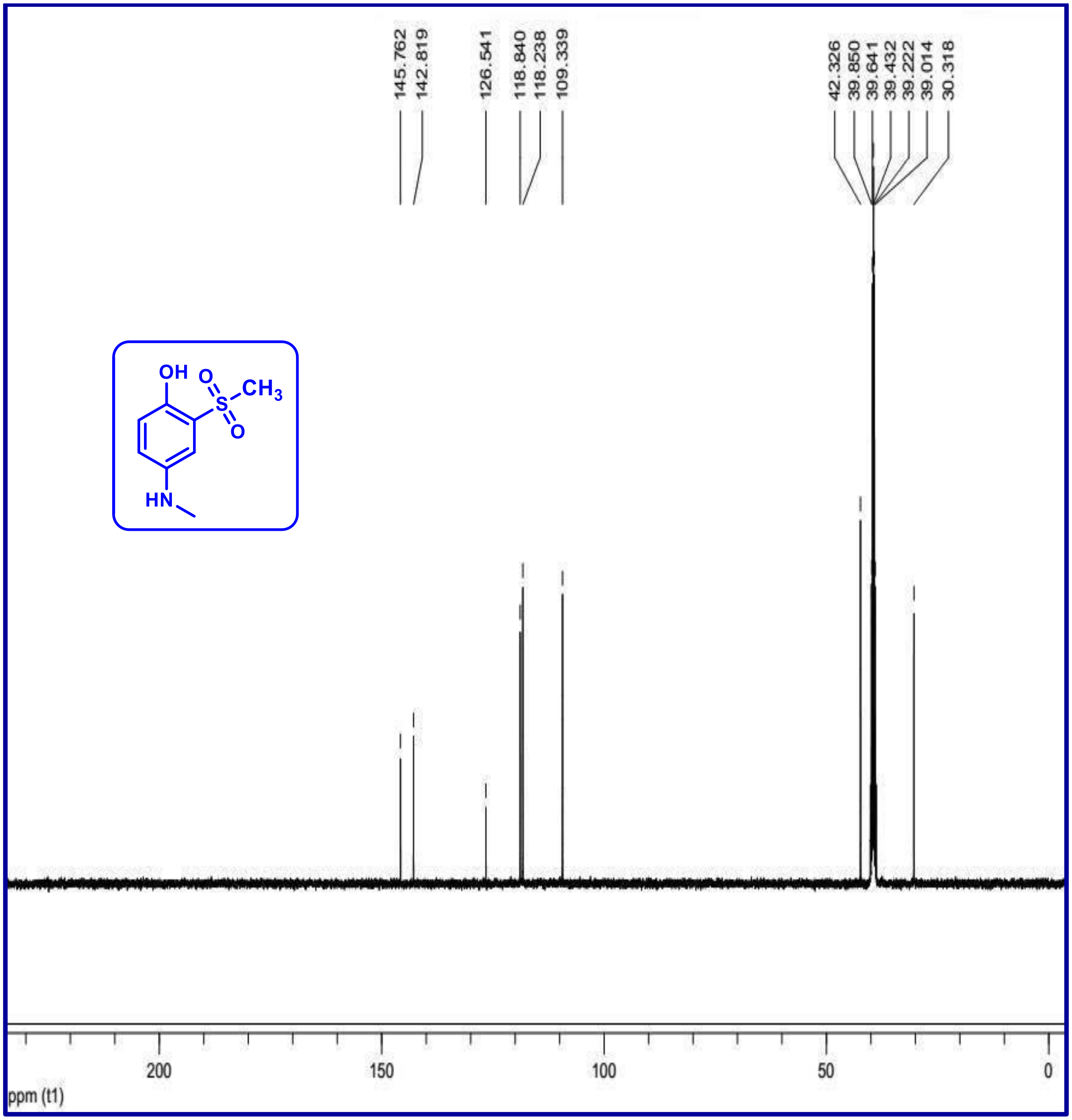

Mass spectrum of MSP4

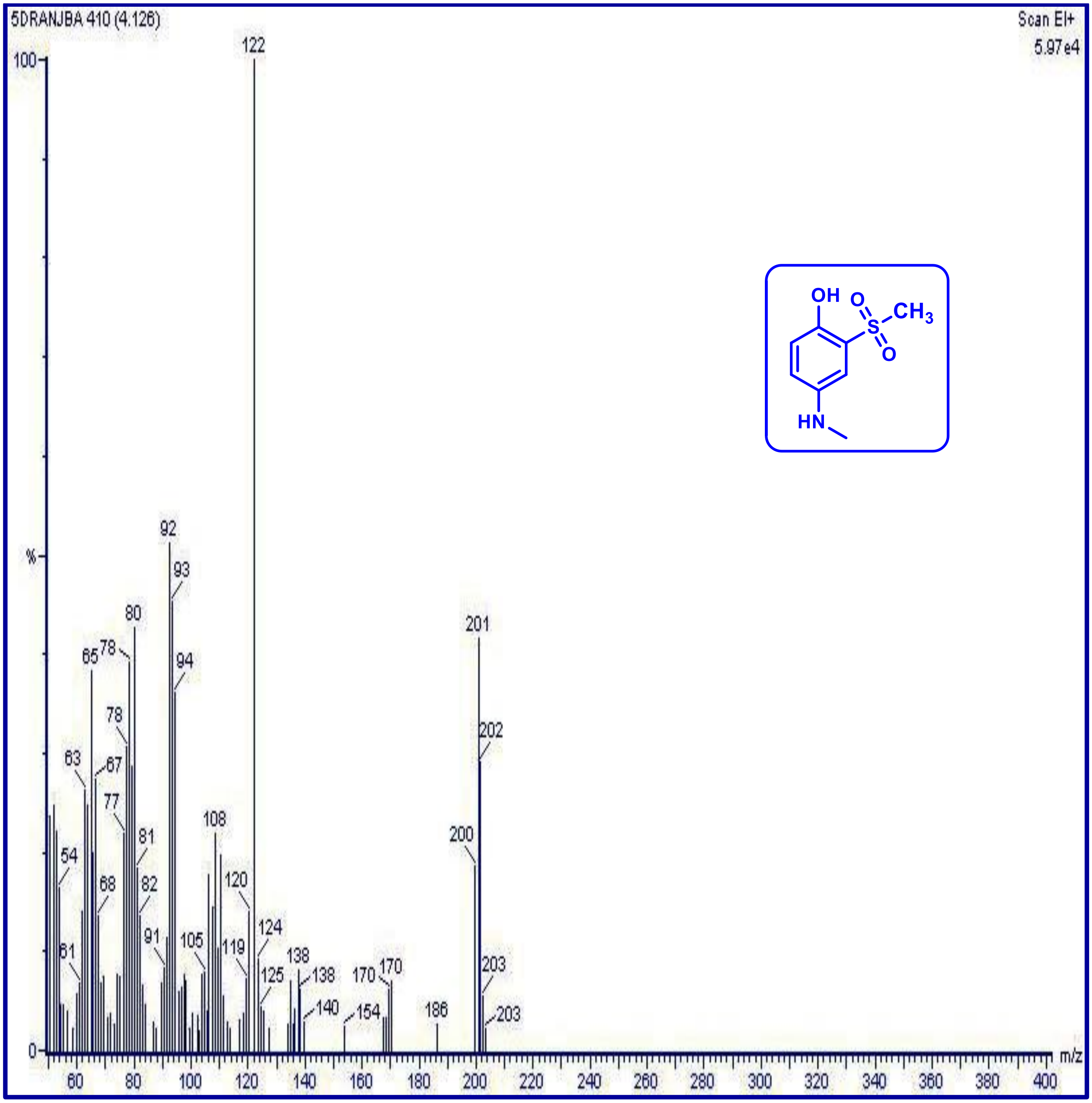

IR spectrum of BSP1

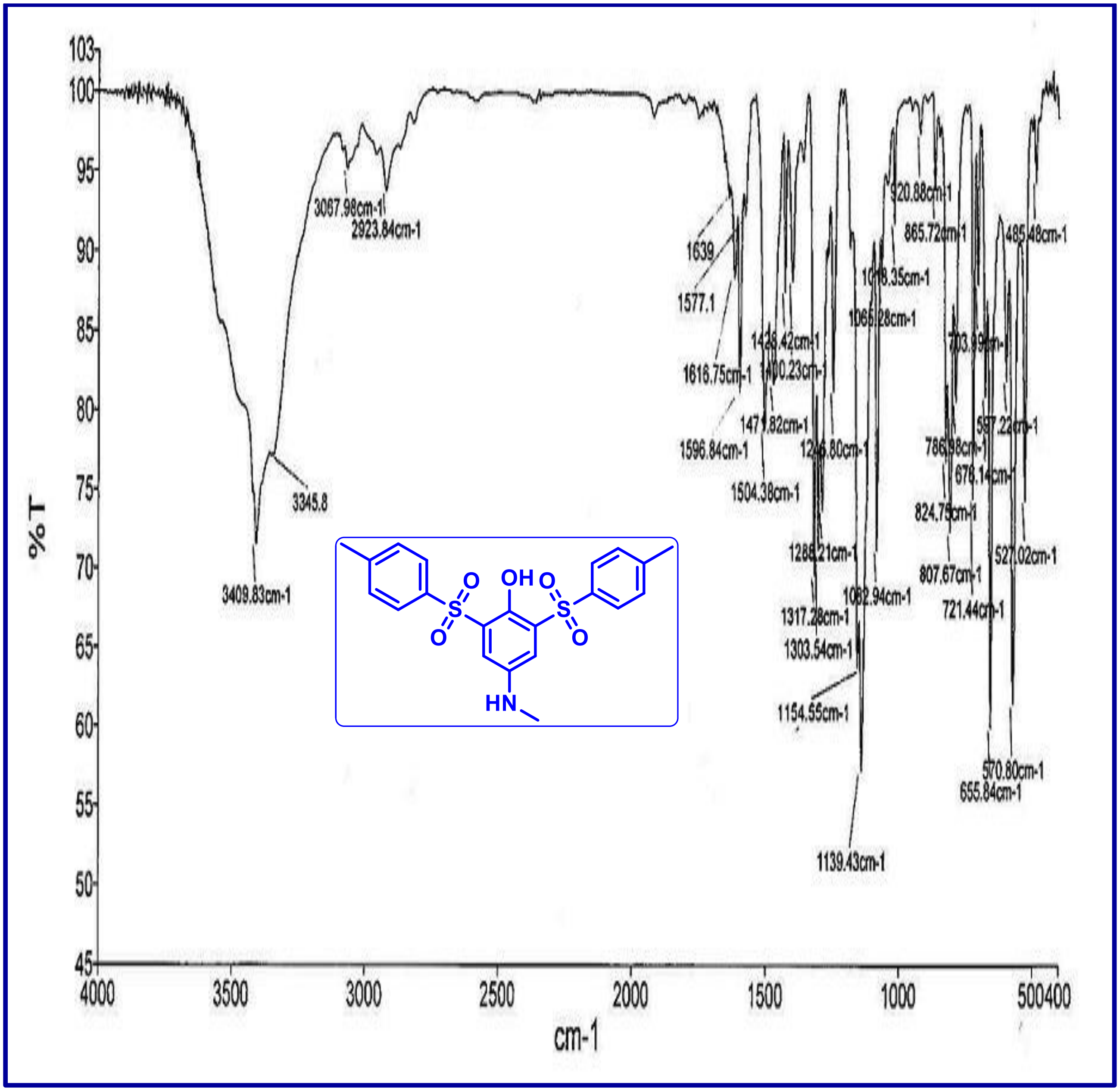

^{1}HNMR spectrum of BSP1

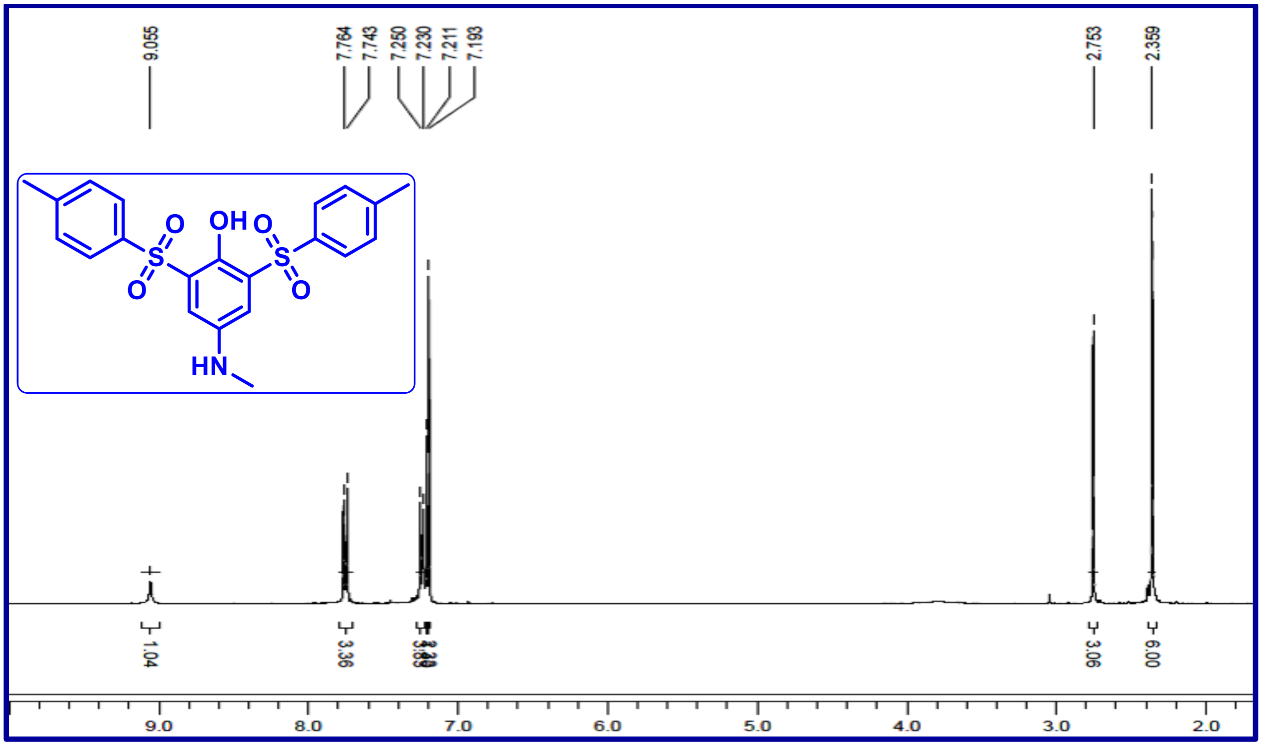

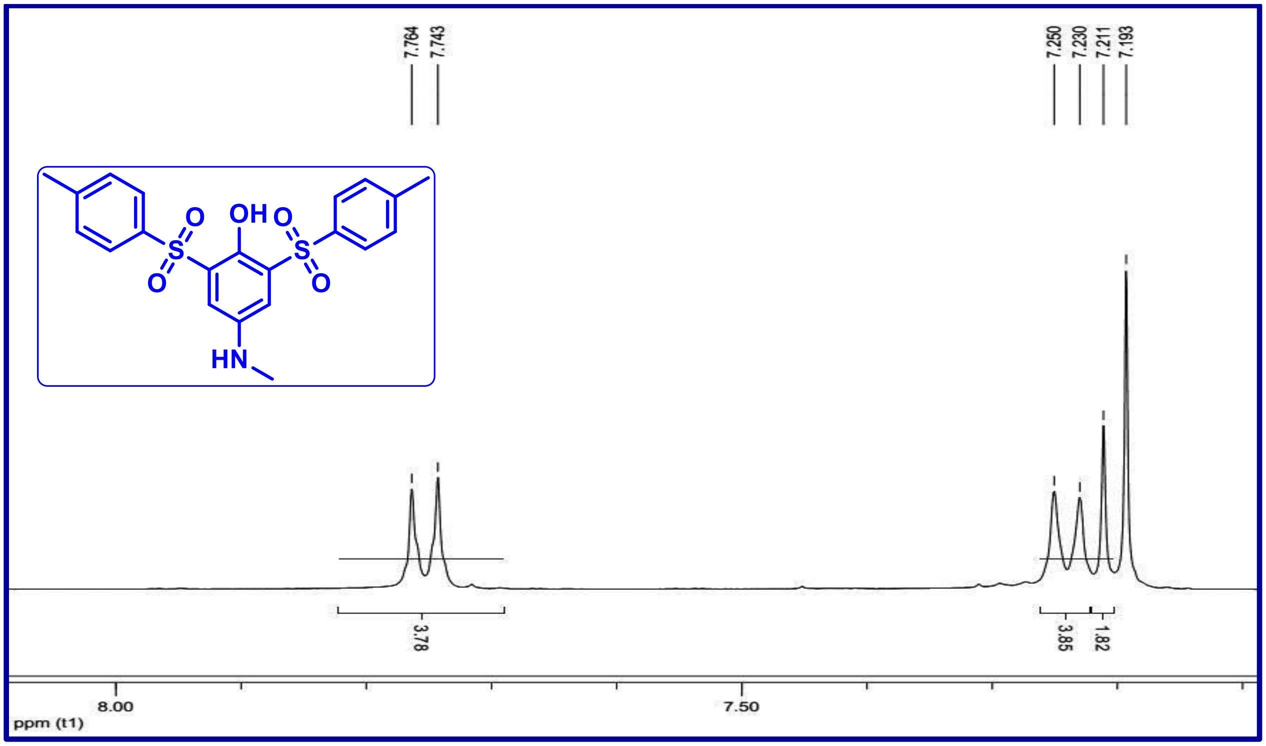

^{13}C NMR spectrum of BSP1

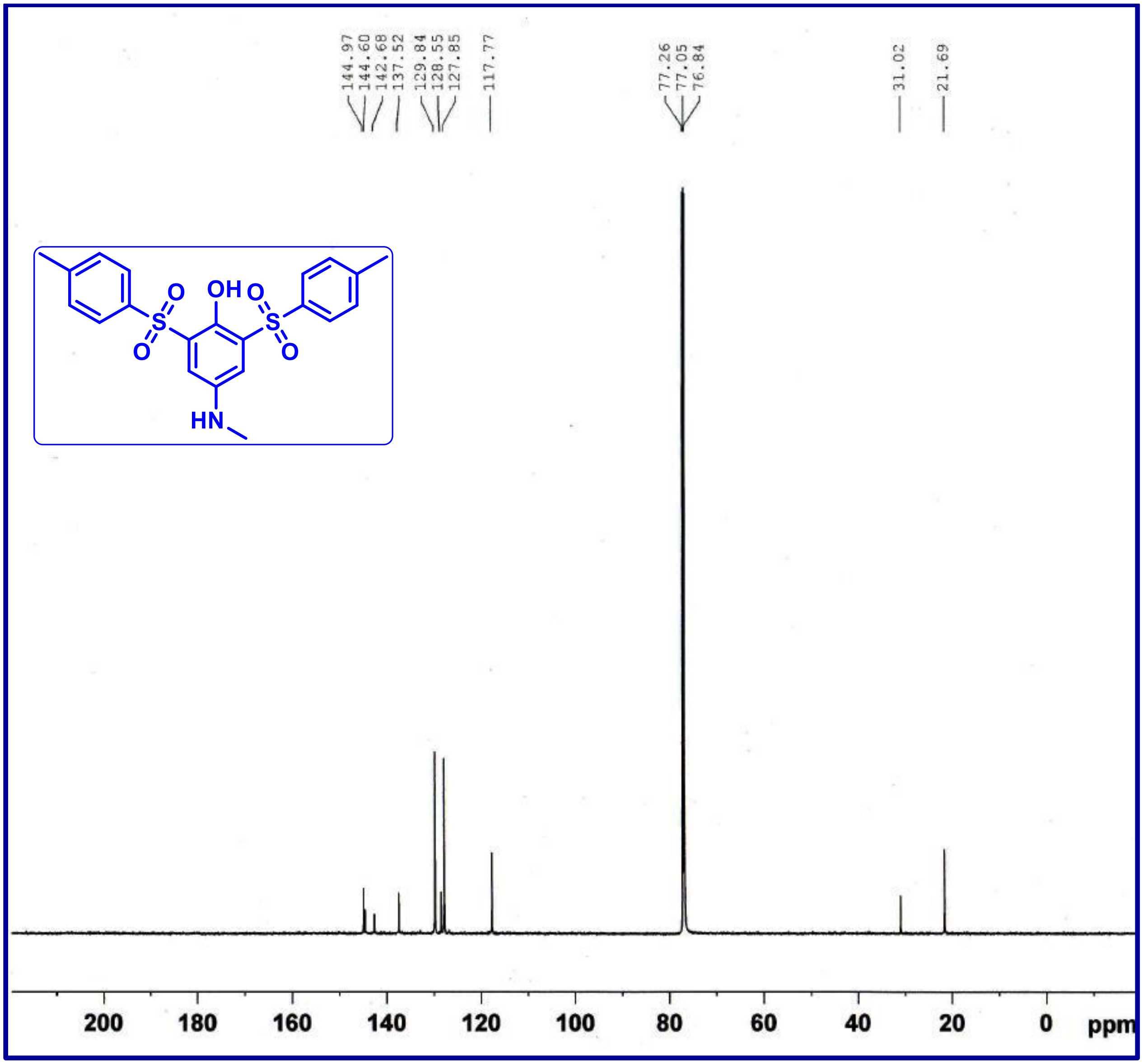

Mass spectrum of BSP1

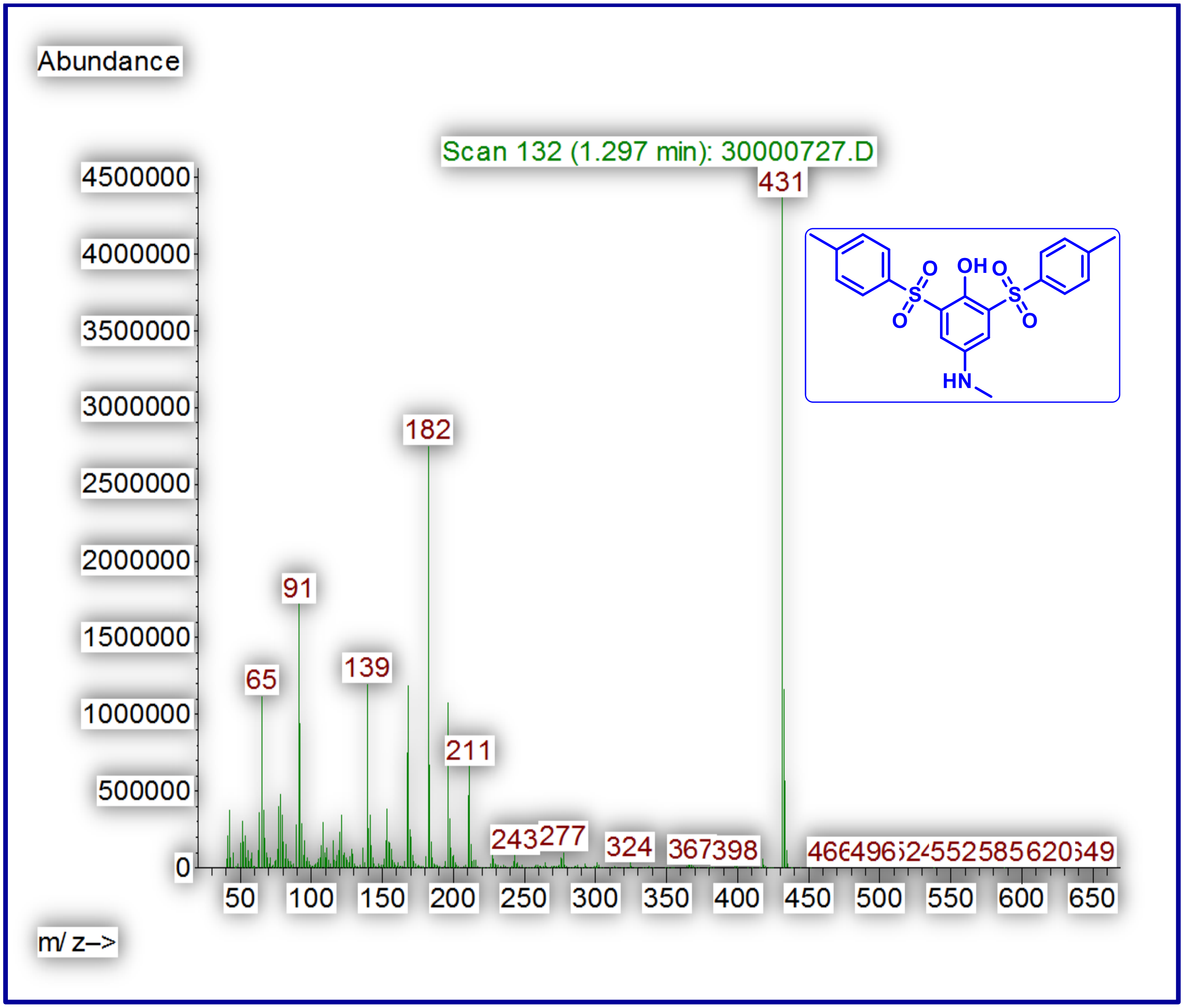

IR spectrum of BSP2

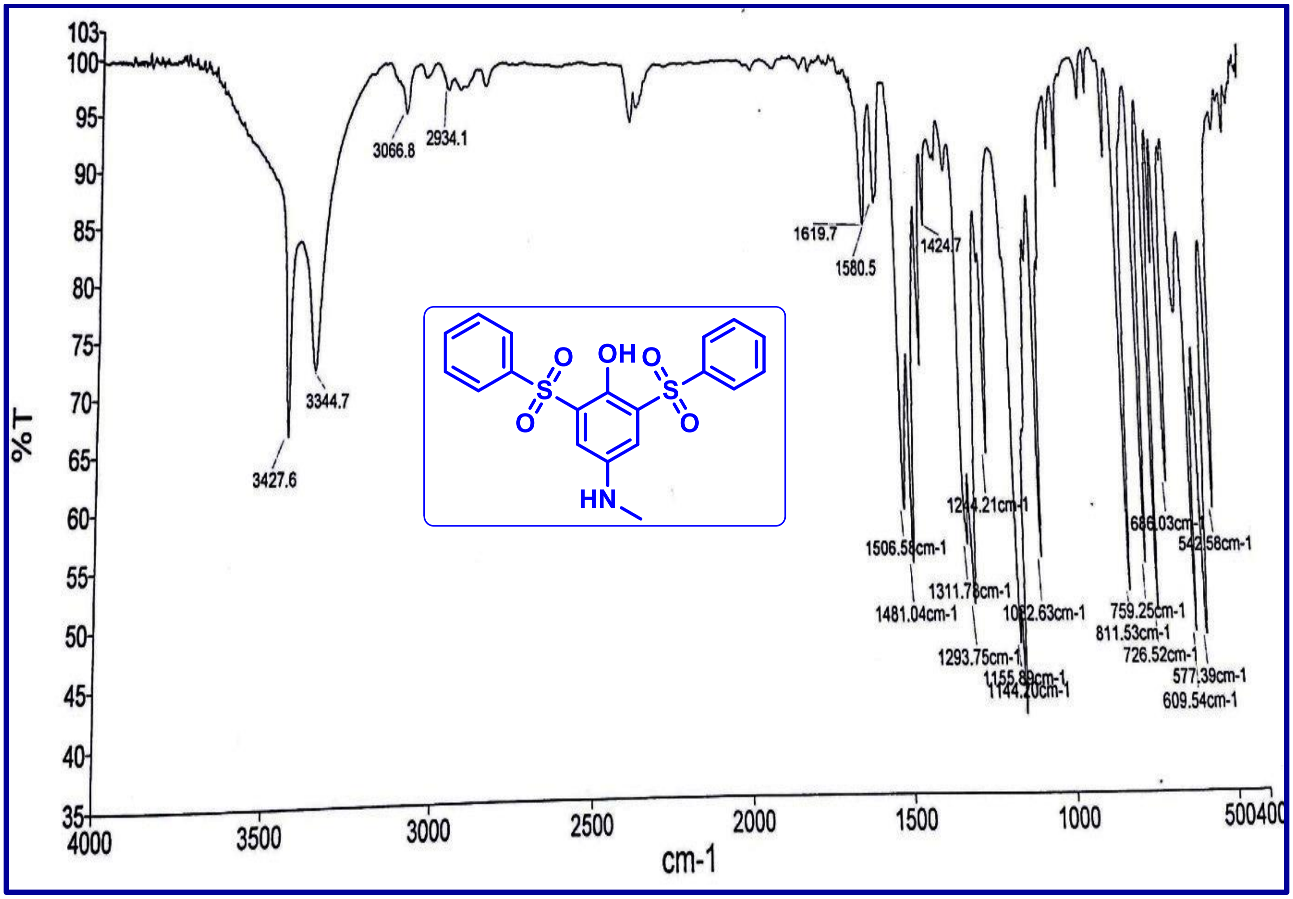

^{1}H NMR spectrum of BSP2

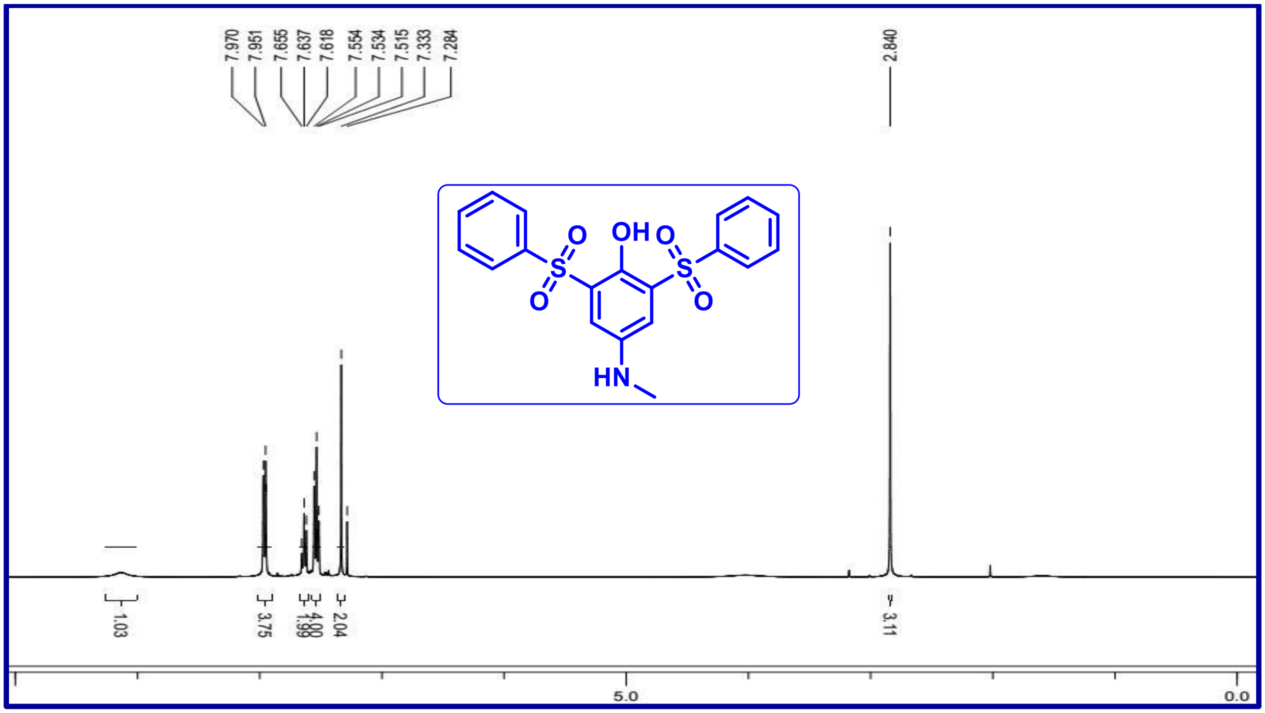

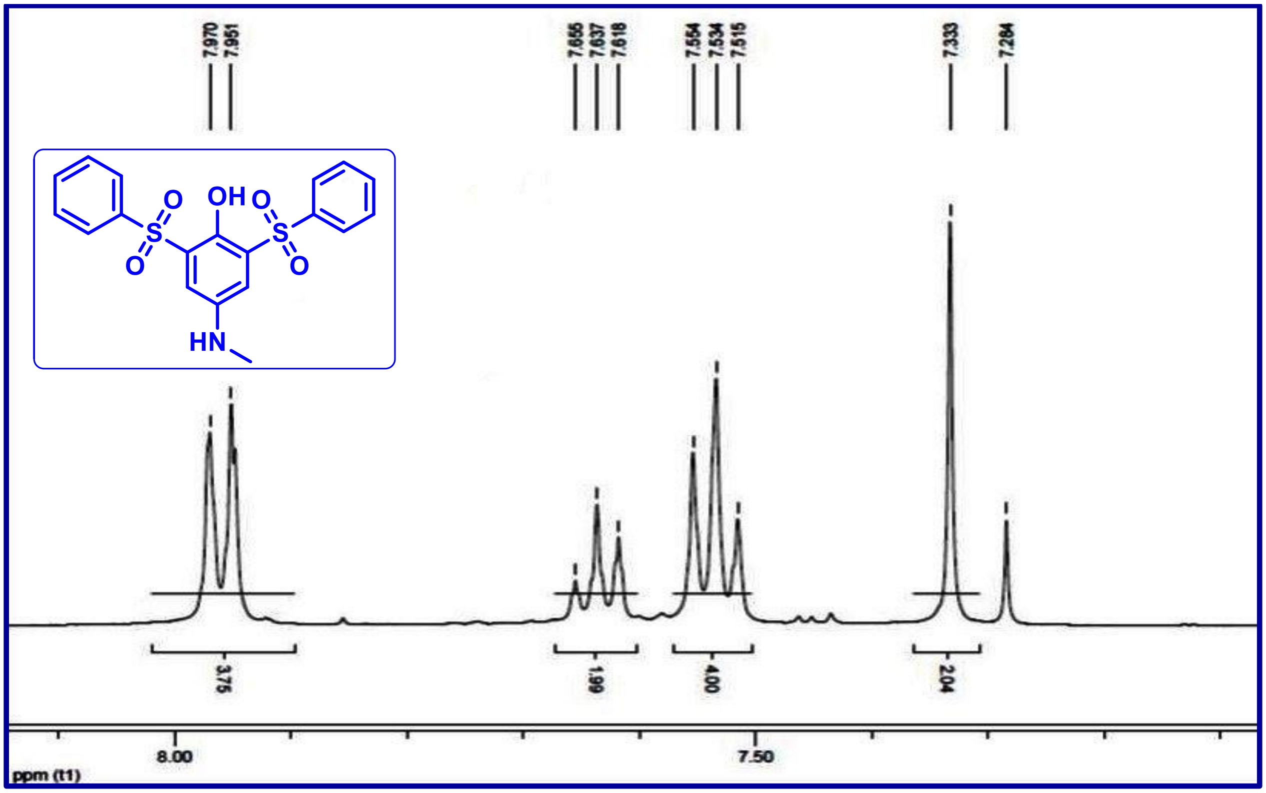

^{13}C NMR spectrum of BSP2

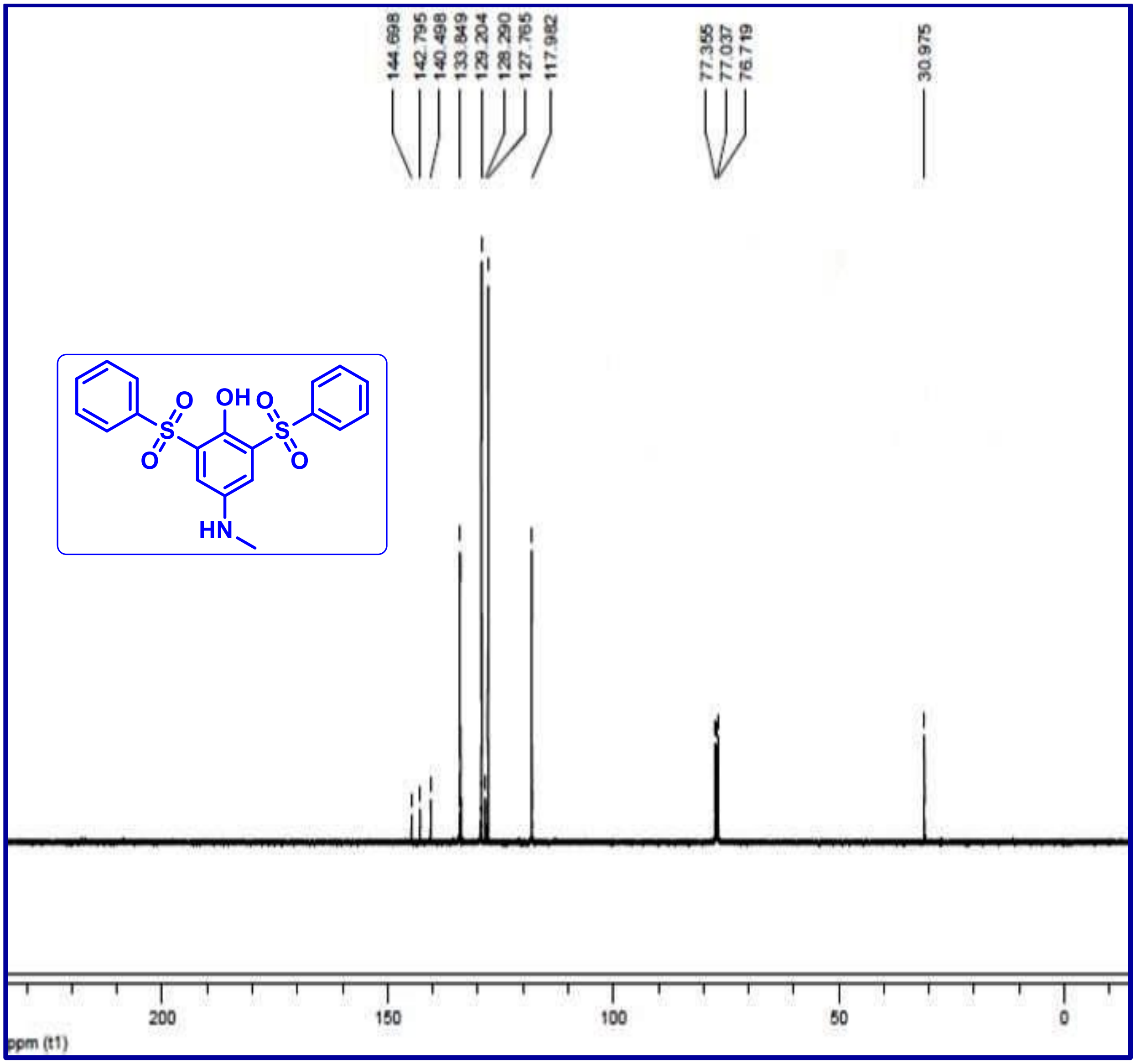

Mass spectrum of BSP2

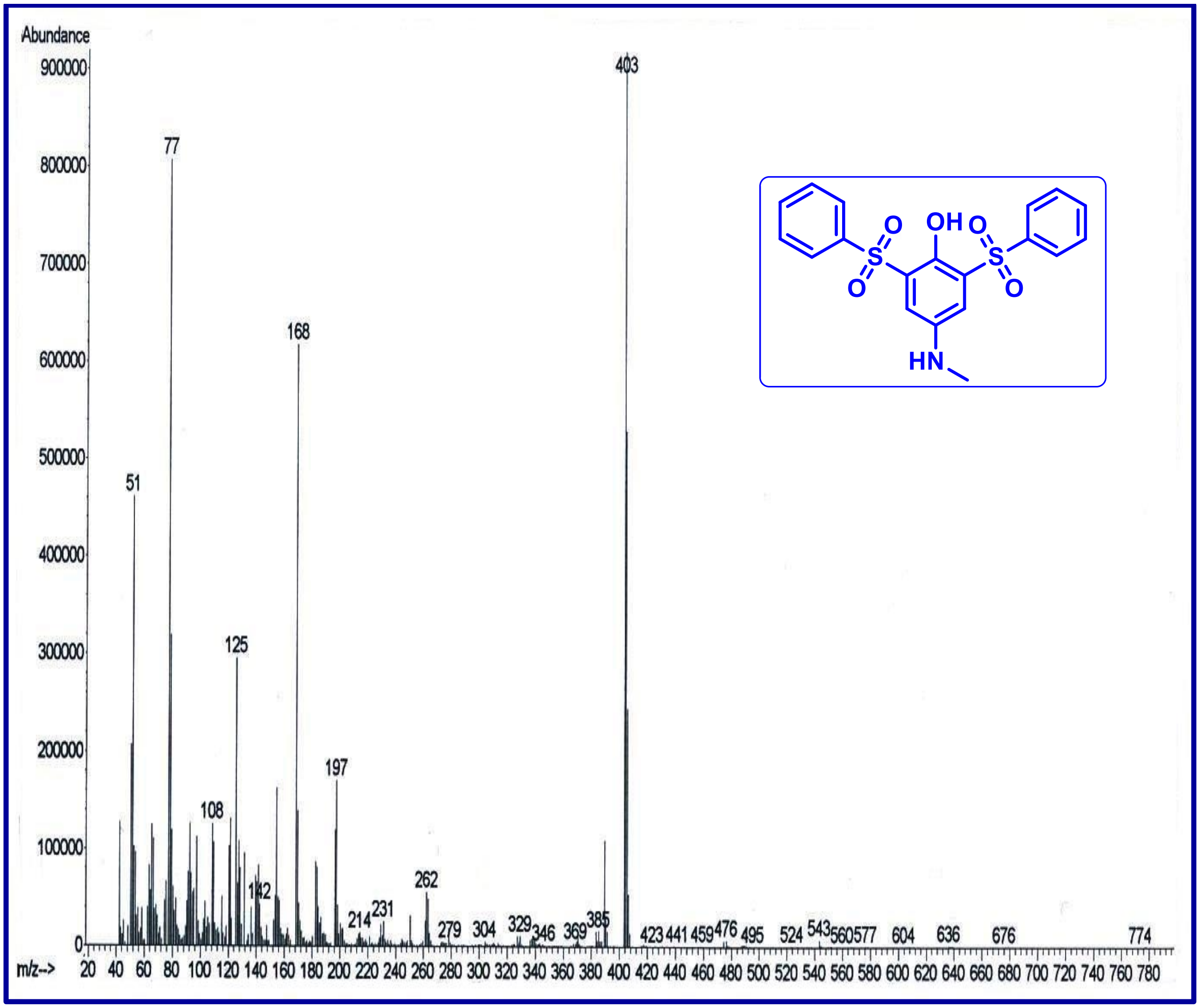

IR spectrum of BSP3

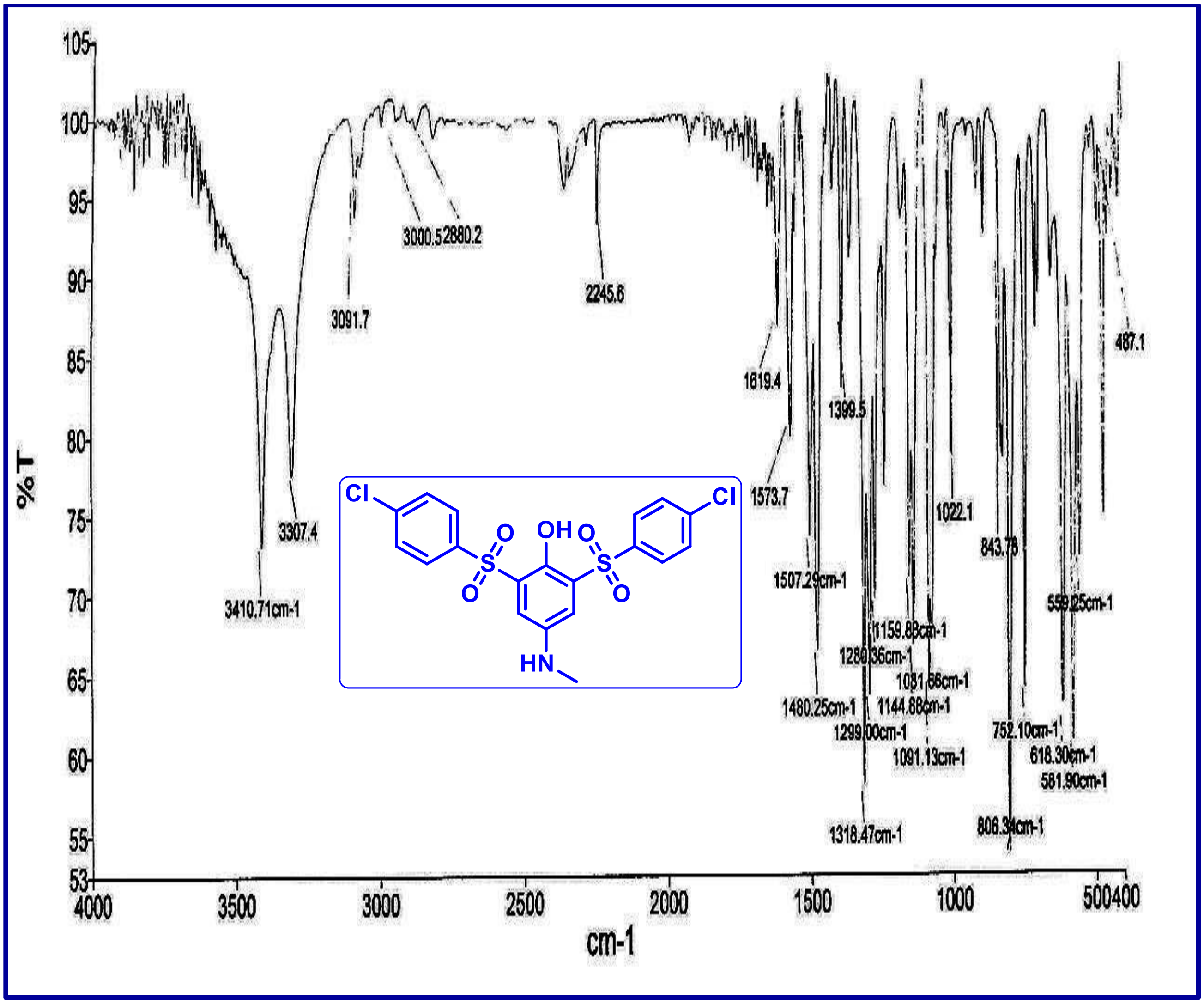

^{1}H NMR spectrum of BSP3

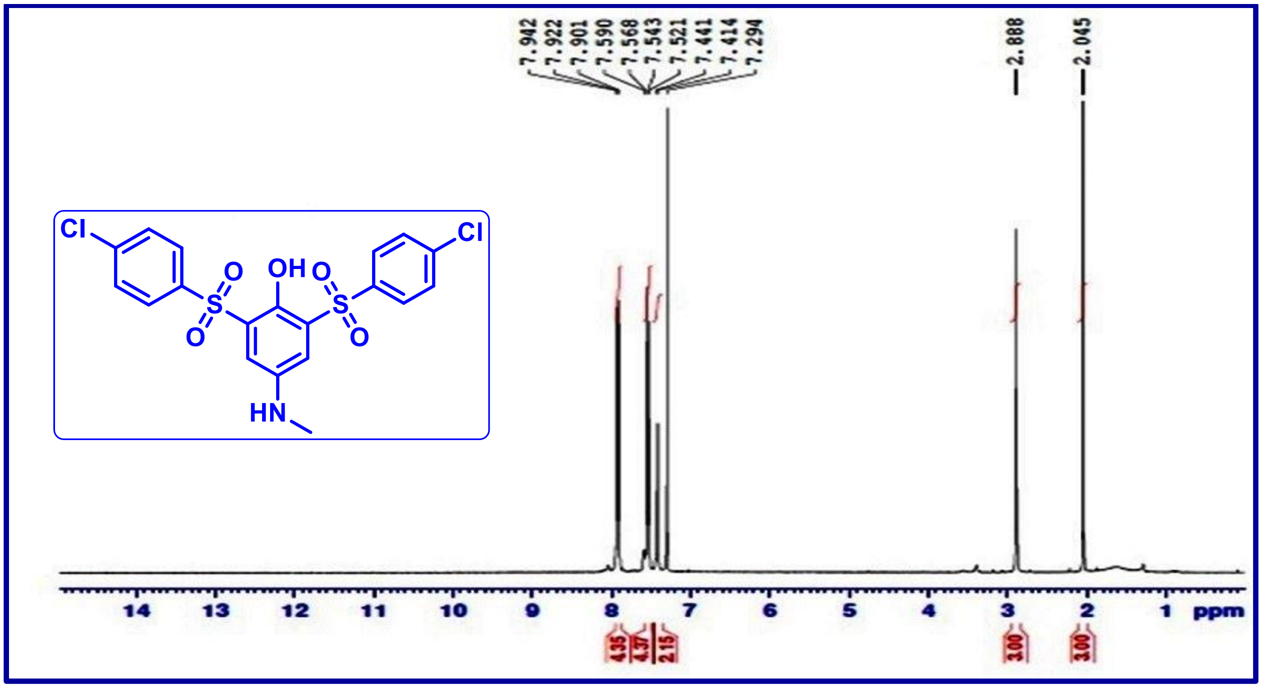

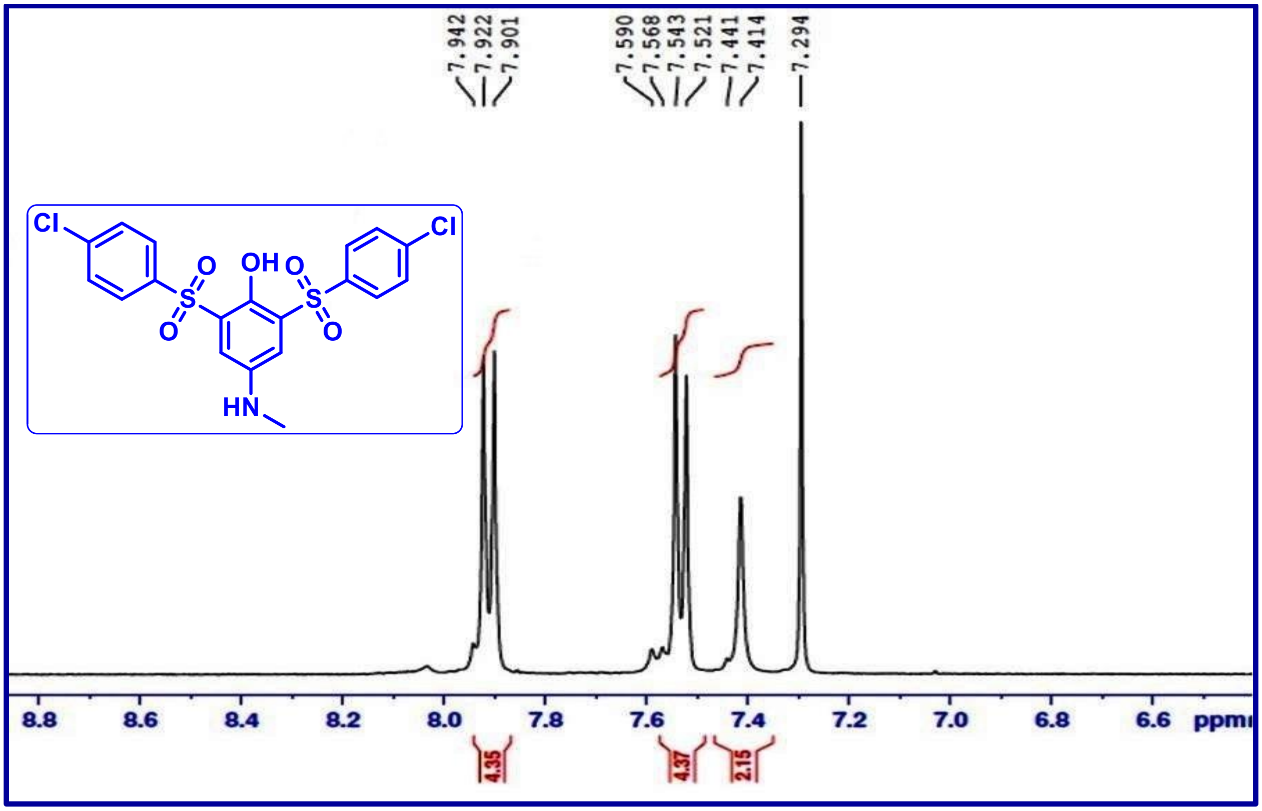

^{13}C NMR spectrum of BSP3

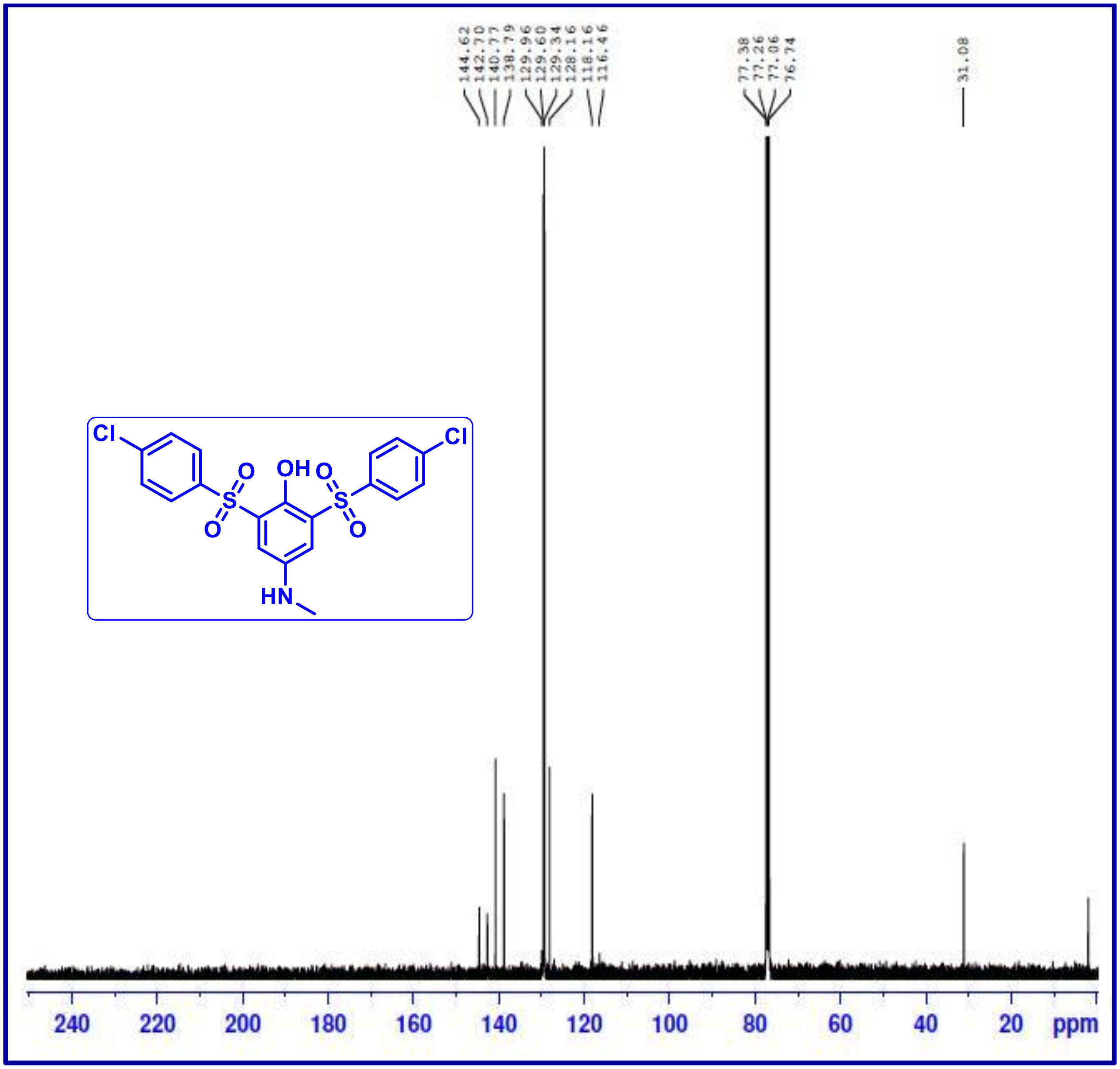

Mass spectrum of BSP3

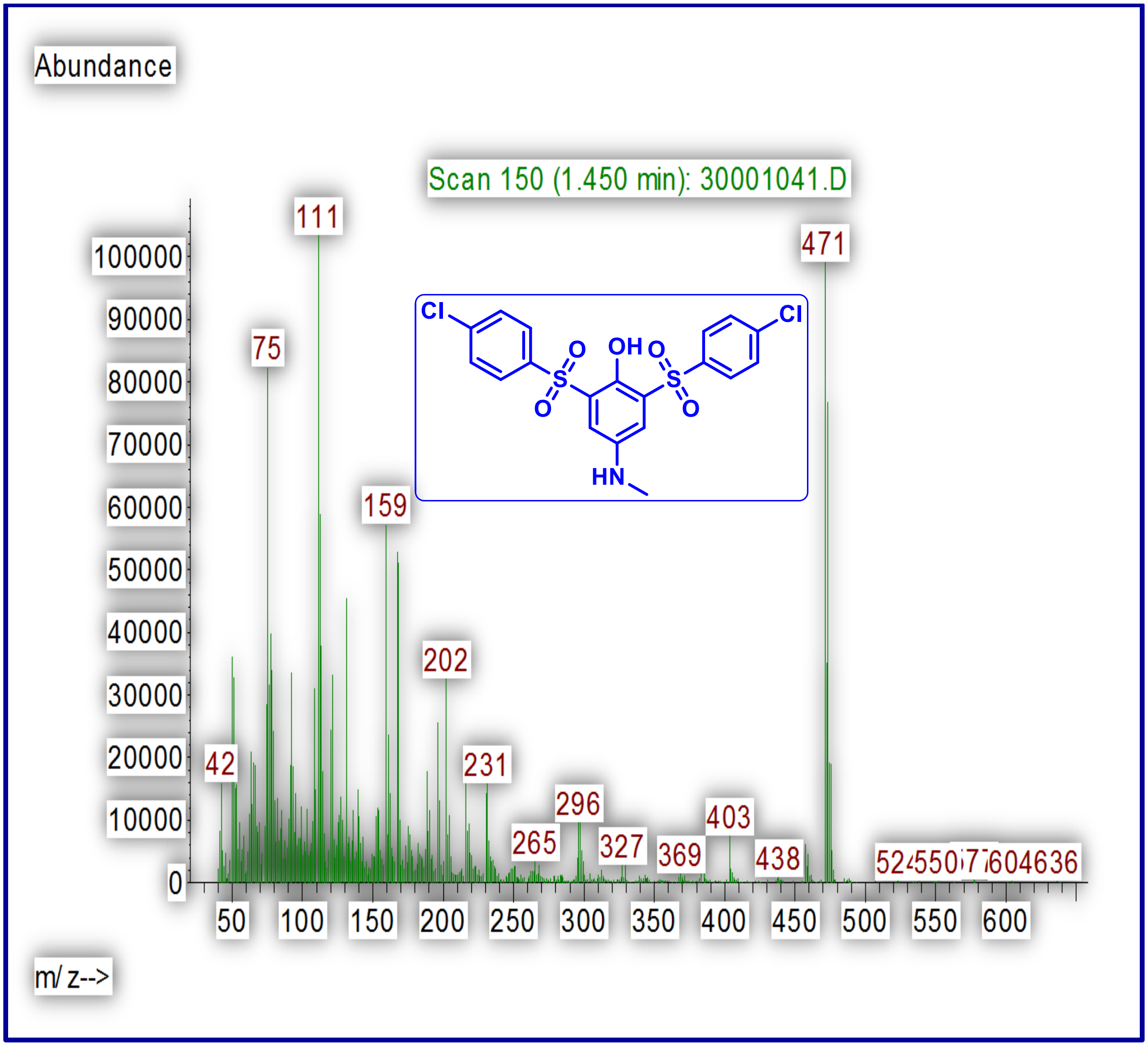

IR spectrum of TSP

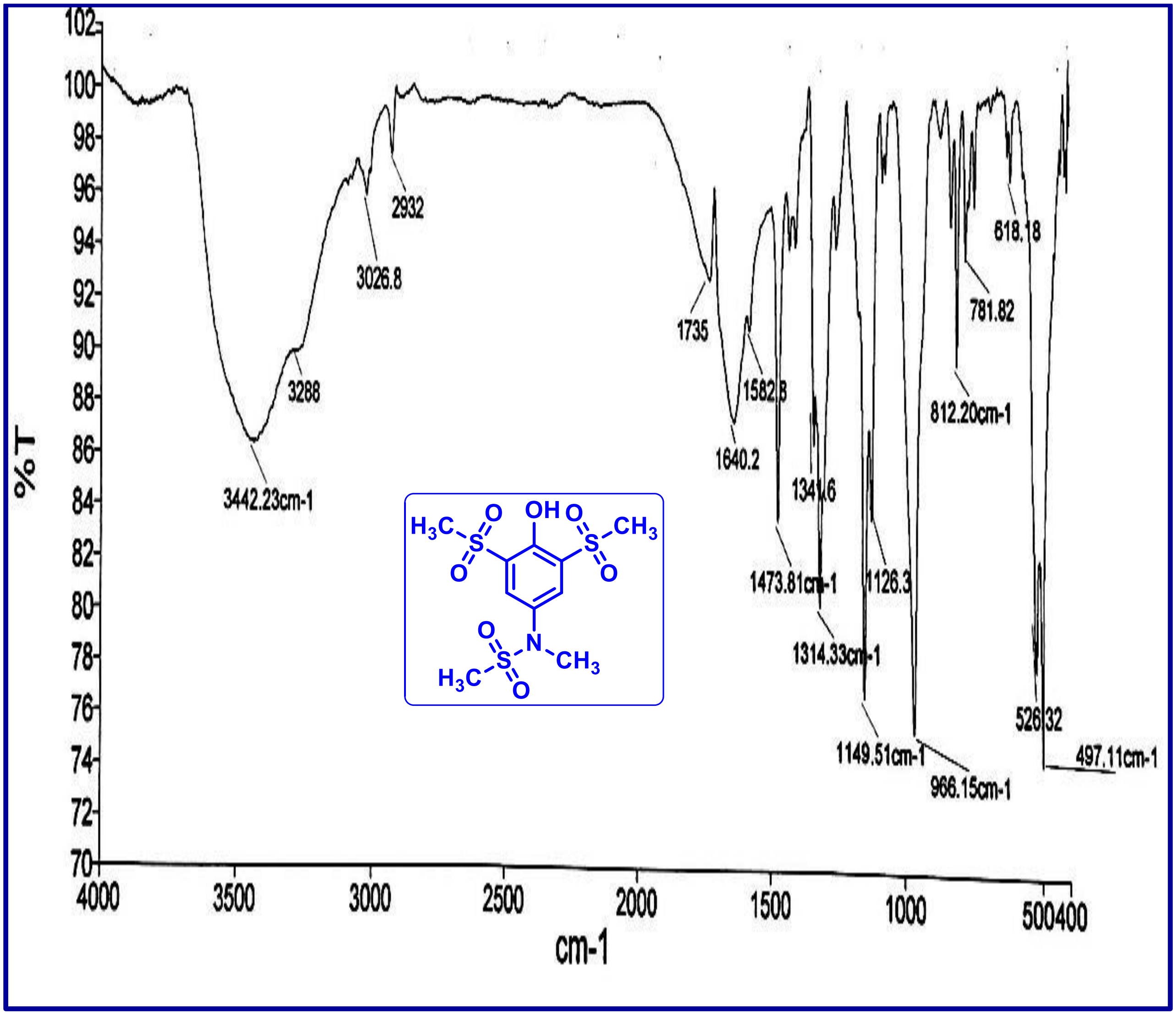

^{1}H NMR spectrum of TSP

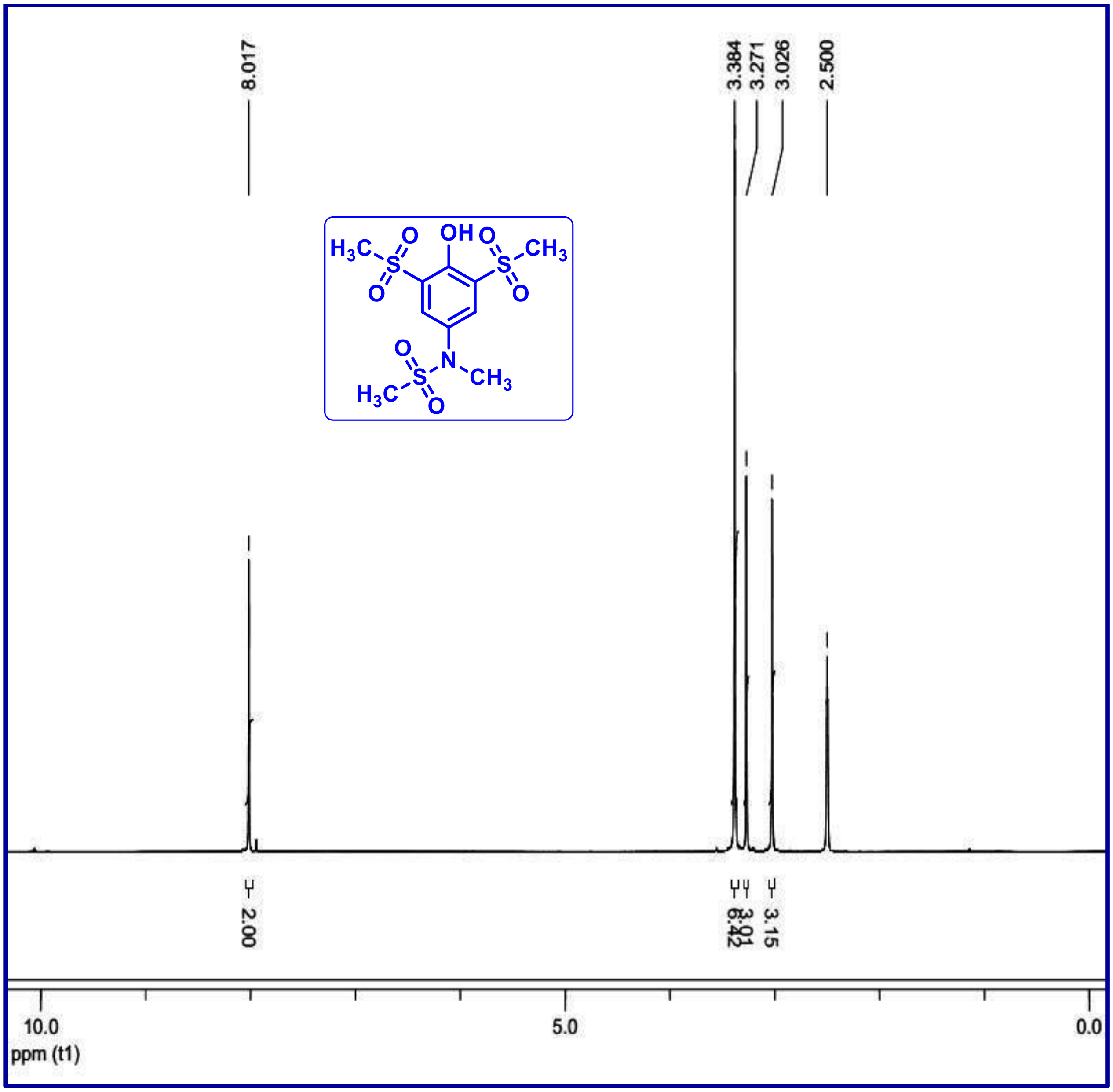

^{13}C NMR spectrum of TSP

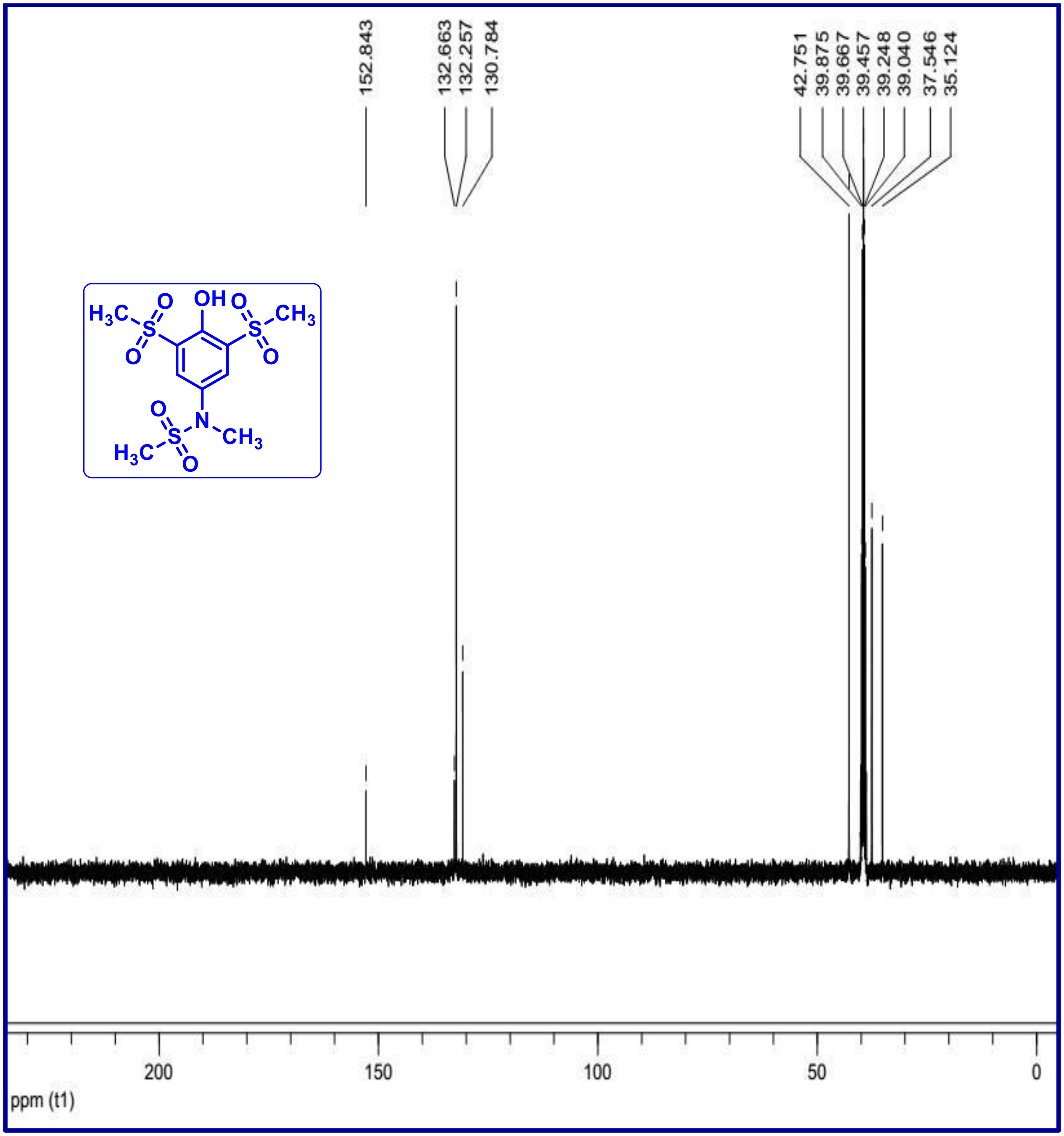

Mass spectrum of TSP

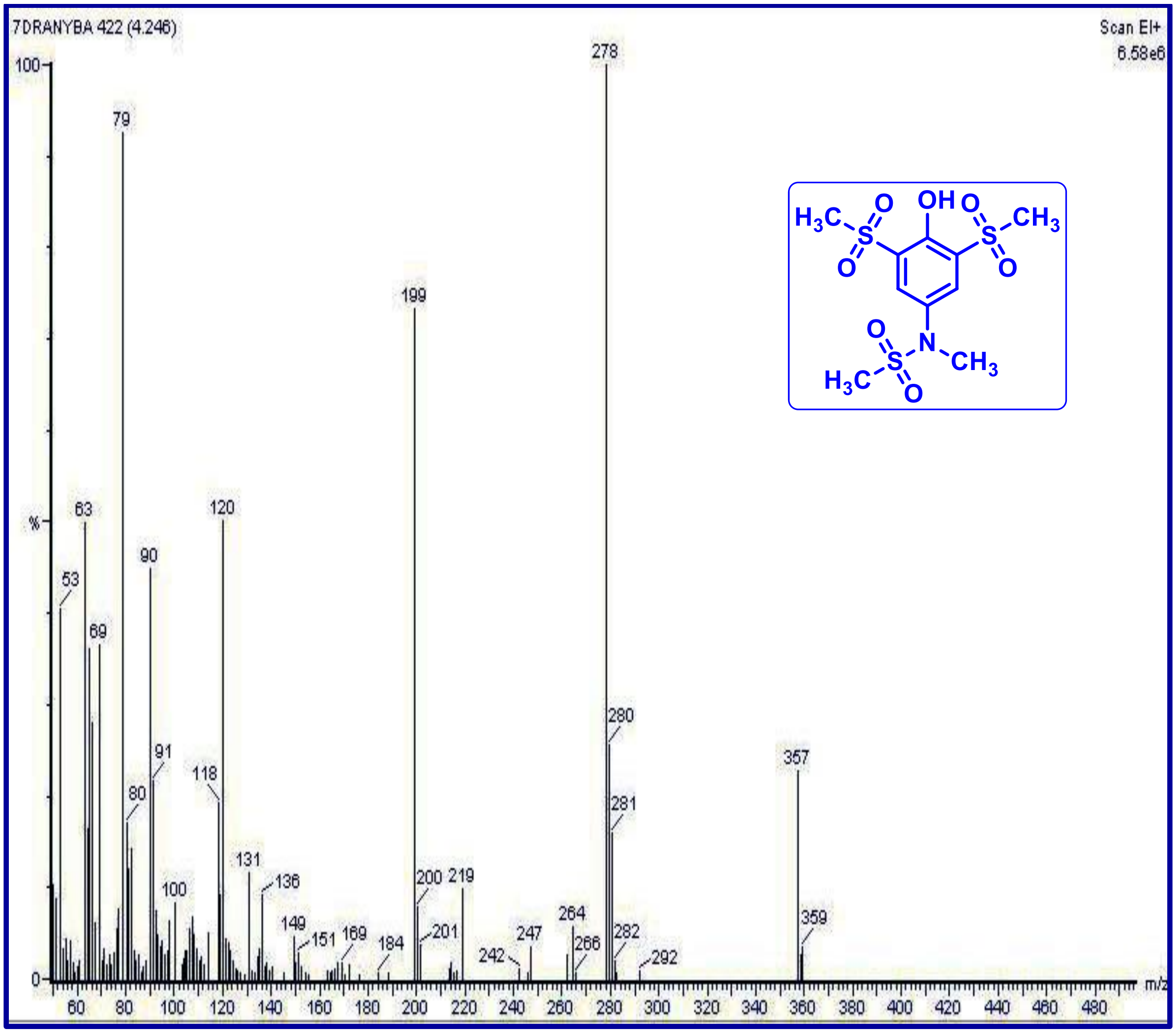

Supplement: Supplementary file 1 — Supplementary information [file 41598_2017_4581_MOESM1_ESM.pdf]
